# Supplementary material for: Native metabolomics identifies the rivulariapeptolide family of protease inhibitors
Source: Nat Commun. 2022 Aug 8;13:4619. doi: 10.1038/s41467-022-32016-6 (PMC9358669; doi:10.1038/s41467-022-32016-6)
Supplement: Supplementary file 1 — Supplementary Information [file 41467_2022_32016_MOESM1_ESM.pdf]

SUPPLEMENTARY INFORMATION

Native Metabolomics Identifies the Rivulariapeptolide Family of Protease Inhibitors

R. Reher et al.

TABLE OF CONTENTS

Content	Page
**Supplementary Methods**	3
**Supplementary figures**	
**Native metabolomics and MS and NMR analyses of complex mixtures**	
Supplementary Figure 1: Optimization of native MS conditions	14
Supplementary Figure 2: Optimization of ammonium acetate buffer concentration	16
Supplementary Figure 3: Selectivity tests and comparison of native metabolomics with direct infusion	17
Supplementary Figure 4: Correlation Networking of metabolite-protein binding with direct infusion	18
Supplementary Figure 5: Mass Spectra from chymotrypsin-rivulariapeptolide complexes from native metabolomics runs	19
Supplementary Figure 6: SMART 2.1 reproducibility test and mixture analysis	20
Supplementary Figure 7: Ion identity network and CANOPUS analysis of *Rivularia* sp fractions	21
**Structure elucidation of rivulariapeptolides and molassamides**	
Supplementary Figure 8: ^{1}H NMR of compound **1** in DMSO-*d*6	22
Supplementary Figure 9: ^{1}H-^{1}H COSY-CLIP of compound **1** in DMSO-*d*6	22
Supplementary Figure 10: ^{1}H-^{13}H HSQC of compound **1** in DMSO-*d*6	23
Supplementary Figure 11: ^{1}H-^{13}C HMBC of compound **1** in DMSO-*d*6	23
Supplementary Figure 12: ^{1}H-^{13}C HSQC-TOCSY of compound **1** in DMSO-*d*6	24
Supplementary Figure 13: ^{1}H-^{1}H NOESY of compound **1** in DMSO-*d*6	24
Supplementary Figure 14: ^{1}H NMR of compound **2** in DMSO-*d*6	*25*
Supplementary Figure 15: ^{13}C NMR of compound **2** in DMSO-*d*6	25
Supplementary Figure 16: ^{1}H-^{1}H COSY of compound **2** in DMSO-*d*6	26
Supplementary Figure 17: ^{1}H-^{13}C HSQC of compound **2** in DMSO-*d*6	26
Supplementary Figure 18: ^{1}H-^{13}C HMBC of compound **2** in DMSO-*d*6	27
Supplementary Figure 19: ^{1}H-^{13}C TOCSY of compound **2** in DMSO-*d*6	27
Supplementary Figure 20: ^{1}H-^{1}H NOESY of compound **2** in DMSO-*d*6	28
Supplementary Figure 21: ^{1}H NMR of compound **3** in DMSO-*d*6	28
Supplementary Figure 22: ^{1}H-^{1}H COSY of compound **3** in DMSO-*d*6	29
Supplementary Figure 23: ^{1}H-^{13}C HSQC of compound **3** in DMSO-*d*6	29
Supplementary Figure 24: ^{1}H-^{13}C HMBC of compound **3** in DMSO-*d*6	30
Supplementary Figure 25: ^{1}H-^{13}C TOCSY of compound **3** in DMSO-*d*6	30
Supplementary Figure 26: ^{1}H-^{1}H NOESY of compound **3** in DMSO-*d*6	31
Supplementary Figure 27: ^{1}H NMR of compound **4** in DMSO-*d*6	31
Supplementary Figure 28: ^{1}H-^{13}C HSQC of compound **4** in DMSO-*d*6	32
Supplementary Figure 29: ^{1}H-^{13}C HMBC of compound **4** in DMSO-*d*6	32
Supplementary Figure 30:^{1}H-^{13}C HSQC-TOCSY of compound **4** in DMSO-*d*6	33
Supplementary Figure 31: ^{1}H-^{1}H ROESY of compound **4** in DMSO-*d*6	33
Supplementary Figure 32: ^{1}H NMR spectrum of compound **5** in DMSO-*d*6	34
Supplementary Figure 33: ^{1}H-^{1}H COSY of compound **5** in DMSO-*d*6	34

Supplementary Figure 34: ^{1}H-^{13}C HSQC of compound **5** in DMSO-*d*6 35
Supplementary Figure 35: ^{1}H-^{13}C HMBC of compound **5** in DMSO-*d*6 35
Supplementary Figure 36: ^{1}H NMR spectrum of compound **6** in DMSO-*d*6 36
Supplementary Figure 37: ^{1}H-^{1}H COSY of compound **6** in DMSO-*d*6 36
Supplementary Figure 38: ^{1}H-^{13}C HSQC of compound **6** in DMSO-*d*6 37
Supplementary Figure 39: ^{1}H-^{13}C HMBC of compound **6** in DMSO-*d*6 37
Supplementary Figure 40: ^{1}H-^{13}C HSQC-TOCSY of compound **6** in DMSO-*d*6 38
Supplementary Figure 41: Mirror MS² plot of molassamide from this study (black) and GNPS library spectrum (green) 38
Supplementary Figure 42: Comparison of 1H spectra of molassamide from this study and molassamide isolated by others 39
Supplementary Figure 43: MS/MS annotations for compounds **1**, **2**, **3** 40
Supplementary Figure 44: Absolute and relative stereochemistry determination for compound **2** 41
Supplementary Figure 45: Docking and structure activity relationship studies of compound **1**-**6** 42
Versatility and specificity of the native metabolomics approach
Supplementary Figure 46: Assessment of accessibility of different protein targets for native metabolomics 43
Supplementary Figure 47: Assessment of native electrospray and in-source dissociation conditions for chymotrypsin and molassamide 44
Supplementary Tables
Supplementary Table 1. NMR table for rivulariapeptolide 1185 (**1**) 45
Supplementary Table 2: NMR table for rivulariapeptolides 1155 (**2**), 1121 (**3**), and 988 (**4**) 46
Supplementary Table 3: NMR table for molassamide (**5**) and molassamide B (***6***) 48
Supplementary Table 4: Mass errors for compounds **1**-**6** 49
Supplementary Table 5: Top 50 chymotrypsin inhibitors among Ahp-yclodepsipeptides 50
Supplementary References 52

Supplementary Methods

Reproducibility of SMART results

Mixture analysis of AHP-cyclodepsipeptide-containing SPE-fraction by acquiring data on two different NMR spectrometers and subsequent SMART 2.1 analysis[1]. The same crude extract was splitted into two equal halves and processed in the same way, but on different days via solid phase extraction into 4 fractions (1-1, 1-2, 1-3, 1-4 & 2-1, 2-2, 2-3, 2-4, respectively). HSQC NMR data for Fraction 1-1 (1 mg) was acquired in a 1.7 mm TCI

MicroCryoProbe (599.10 MHz) to obtain the correlations of the major components of that fraction in about 13 minutes by applying non uniform sampling[2] and Acceleration by Sharing Adjacent Polarization[3] protocols. HSQC NMR data for Fraction 2-1 (1 mg) was acquired on a JEOL ECZ 500 NMR spectrometer equipped with a 3 mm inverse detection probe to obtain the correlations of the major components of that fraction in about 14 hours. The raw data was processed (phase correction, baseline correction, removal of t1 noise) with MestreNova Version 12.0 and the major correlations were manually peak picked. The resulting table was exported and analyzed by SMART 2.1 (https://smart.ucsd.edu/classic).

In silico MS/MS annotation

Comparison of the SMART results revealed that eight of ten predicted structures were identical and five or respectively six out of ten compounds were AHP-cyclodepsipeptides. In depth analysis of the top ten predicted structures with NPClassifier[4] (https://npclassifier.ucsd.edu/) showed that all, but one structure were classified as cyclic depsipeptides and all of the structures were further classified as polyketides, suggesting a NRPS/PKS hybridic pathway for the biosynthesis of the main compound in fraction 1-1 (and 2-1, respectively).

We annotated the Ahp-cyclodepsipeptide candidates as members of the subfamily rivulariapeptolides followed by their molecular weight if CANOPUS[5] detected the substructure element 'delta-lactam' or 'piperidone' with a posterior probability of > 99% in combination with the detection of substructural elements such as 'pyrrolidinecarboxamide', '*N*-acylpyrrolidines', and 'proline and derivatives' with a posterior probability of > 90%, indicative of the characteristic *N*-butyrylated proline residue. All MS/MS of isolated compounds were deposited as public spectra within GNPS[6] along with confirmed activity of the respective compounds as protease inhibitors utilizing GNPS tags.

Relative and absolute configurations of the rivulariapeptolides

The absolute configurations of the amino acids were determined by UHPLC-MS analysis of the acid hydrolysates of 2 (rivulariapeptolide 1155) and its PDC oxidation product (**Supplementary Figure 41a**). Oxidation followed by acid hydrolysis liberated glutamic acid, allowing the assignment of the C-3 position of the Ahp (3-amino-6-hydroxy piperidone) unit. The analysis revealed L-configuration for C-3 as well as for the C-alphas of every further amino acid in the rivulariapeptolides (as it is the case for all previously reported cyanobacterial Ahp-cyclodepsipeptides). The relative stereochemistry of the Ahp moiety was determined to be (3S*, 6R*)-Ahp based on NMR spectroscopic data (**Supplementary Figure 41b**). In the Ahp ring system, NOESY correlations were observed between the diaxially oriented H-3 (δ 3.78 ppm), H-5a (δ 1.71 ppm), and the equatorially oriented H-6 (δ 5.07 ppm, br s ($J < 1$ Hz))[7]. Thus, the hydroxyl group of C-6 had to be axially oriented and this axial orientation of the 6-OH group is responsible for the downfield shift of H-4a (δ 2.42 ppm). Furthermore, the NOESY correlations between H-3 (δ 3.78 ppm) and the equatorial H-4b (δ 1.58 ppm) supported the assigned relative stereochemistry. Together with the results from the PDC oxidation and Marfey's analysis we determined the absolute configuration to be (3S, 6R)-Ahp in line with the stereochemical assignments in other Ahp-cyclodepsipeptides such as tutuilamide A and molassamide, respectively[8,9]. The geometry of the Abu olefinic bond was determined as "Z" based on HMBC and NOE correlations in DMSO-*d6*. A four-bond HMBC correlation between H_3-4 and C-1 of Abu indicates a "w" configuration for bonds between these atoms and, therefore, the Z-geometry for the double bond[10]. NOESY correlations from H_3-4 of Abu (δ 1.51) to H-2 of Thr-1 (δ 4.59) and H_3-4 of Thr-2 (δ 1.11) further support this configuration.

Pyridinium dichromate (PDC) oxidation

Rivulariapeptolide 1155 (Compound 2, 0.5 mg) was dissolved in CH_2Cl_2 (0.5 mL) and mixed with PDC (2.0mg). After stirring at rt for 5h, the reaction was quenched by shaking with 3 x 1 mL portions of H_2O, and the the CH_2Cl_2 phase was dried under N_2. The resulting oxidized material was analyzed by advanced Marfey's method and subsequent UHPLC-MS as described below.

Advanced Marfey's method

Rivulariapeptolide 1155 (Compound 2, 0.3 mg) and the residue of the PDC reaction were hydrolyzed with 6N HCl (0.3 mL) and heated at 110°C for 16 hrs in sealed tubes. The hydrolysates were concentrated to dryness under N_2, and each treated with a solution of 1-fluoro-2-4-dinitrophenyl-5-L-alanine amide (FDAA, 1 mg/mL) in acetone (100 µL) and a solution of 1 M $NaHCO_3$ (0.3 mL) in a sealed vial at 90 °C for 5 min. The reaction mixture was neutralized with 1N HCl (300 µL) and diluted with CH_3CN (0.7 mL). The resulting solution was analyzed by LC-MS via a Vanquish UHPLC System coupled to a Q-Exactive HF Mass Spectrometer (Thermo-Fisher). RP-UHPLC was performed on a Kinetex C18 column with 150 x 2 mm 1.8 um particle size, 100 A pore size. The gradient elution profile started at 5% Acetonitrile (ACN) /95% H_2O (acidified with 0.1% formic acid (FA)) and was ramped to 30% ACN at 8 min following an increase to 99% ACN at 10 min. The column was then washed for three minutes at 99% ACN, and then re-equilibrated over 3 min at 5% CAN. The flow was set to 0.5 mL/min. Electrospray ionization (ESI) parameters were set to 50 arbitrary units (AU) sheath gas flow, auxiliary gas flow was set to 12 AU and sweep gas flow was set to 1 AU. Auxiliary gas temperature was set to 400 °C. The spray voltage was set to 3.5 kV and the inlet capillary was heated to 250 °C. S-lens level was set to 50 V applied. MS scan range was set to 200-2000 *m/z* with a resolution at *m/z* 200 ($R_{m/z\ 200}$) of 120,000 with one micro-scan. The maximum ion injection time was set to 50 ms with automatic gain control (AGC) target of 3E6. Raw files were converted to .mzML format using MSconvert from the proteowizard software package. Extracted ion chromatograms (XICs) of the expected amino acid-FDAA conjugates were generated with the GNPS Dashboard with 5 ppm mass tolerance. Retention times and XICs of the amino acid-FDAA conjugates from the reaction mixtures were compared with the commercially available amino acid standards that were derivatized using identical methodology and allowed us to establish **2**.

Docking and structure activity relationship studies reveal potential for therapeutic optimization (Supplementary Figure 45)

Docking studies were performed to to rationalize the bioactivity of the isolated compounds and to explore the potential for structure modifications that could yield lead structures for therapeutic interventions. The isolated compounds were docked by induced-fit, inside the

binding pocket of alpha-chymotrypsin (PDBID 4Q2K) and all were found to have a similar binding mode (Supplementary Figure 45b, d, e). Crystal structures of Ahp-cyclodepsipeptides in complex with serine proteases by others[8,11] indicate that inhibition is based on a substrate-like binding mode in which distinct amino acid residues occupy the S- and S'-pockets, however, proteolytic cleavage does not occur, because the AHP moiety occupies an important part of the active site pocket. Furthermore, it has been demonstrated that the residue immediately following the Ahp unit should bind in the S1 specificity-determining pocket[12,13]. Structure-activity relationships towards elastase inhibition revealed that the 2-amino-2-butenoic acid (Abu) moiety incorporated within the macrocycle, contributes to potent elastase inhibition[8,14]. This is in accordance with our experimental results where the replacement of the Abu (compound **2**) moiety for Leu (**1**) led to a fivefold decrease in potency towards elastase, but simultaneously to a more than threefold increase in potency towards chymotrypsin. The previously reported importance of a polar functionality in the side chain towards more potent elastase inhibition could not be corroborated by our data[11,14]. Masking, the secondary alcohol of the threonine in the side chain by an esterification with *N*-butyryl proline (**2**) instead of bearing the free polar hydroxyl group (**4**) increased the potency towards elastase. Binding to proteinase K, however, seems to be more favorable with less rigid and bulky side chain substituents as found in compounds **5** and **6**.

Structural analysis

We modeled the rivulariapeptolides peptides bound to α-chymotrypsin, based on the crystal structure (PDB ID 4GVU) of the macrocyclic peptide lyngbyastatin 7, which shares a chemical scaffold with the rivulariapeptolides, bound to elastase, which has 37% sequence identity with alpha-chymotrypsin and a similar three-dimensional structure. A crystal structure of alpha-chymotrypsin (PDB ID 4Q2K) was aligned with the elastase structure, using the Molecular Operating Environment (MOE, version 2019.01) software, transferred the lyngbyastatin 7 molecule to the chymotrypsin structure, and energy-minimized the resulting bimolecular complex. We then used ChemDraw integration with MOE to sketch each of the rivulariapeptidolides and energy-minimized each resulting

complex. Minimization was performed using the Amber10:EHT force-field, no constraints were applied, and the convergence criterion was set to RMS of 0.1 kcal mol^{-1} $Å^2$.

Commercial Proteins

Chymotrypsin (*Bos taurus*), Elastase (*Sus domesticus*) and Bovine Serum Albumin (*Bos taurus*) were purchased from Sigma-Aldrich, St. Louis, Missouri, United States. Proteinase K (*Tritirachium album*) was purchased from Roche, Basel, Switzerland. Transferrin (*Homo sapiens*) was purchased from Behringwerke AG, Marburg, Germany.

Protein Expression and Purification

Cloning and protein purification of TasA

Plasmid pET22b-tasA was constructed by amplifying *tasA* gene from *B. subtilis* NCIB3610. The fragment of DNA containing the open reading frame without signal peptide or codon stop was amplified by PCR and then digested with NdeI and XhoI enzymes and cloned into the plasmid pET22b, cut with the same enzymes. The plasmid pET22b-tasA was transformed in BL21 (DE3) competent *E. coli* cells. Cultures in 100 mL of LB supplemented with 100 µg/mL ampicillin were grown shaking at 37°C until an optical density at 600 nm (OD600) of 0.7 was reached. Isopropyl β-D-1-thiogalactopyranoside (IPTG) was added to a final concentration of 1 mM and the cultures were incubated for additional 24 h at 28°C with shaking in order to induce the formation of inclusion bodies. Next day, cells were harvested by centrifugation (7,000 × g, 10 min, 4 °C) and resuspended in buffer A (50 mM Tris, 150mM NaCl, pH 8), and then centrifuged again. The pellets were kept at −80 °C until purification or processed after 15 min. Cells were resuspended in buffer A, sonicated on ice (3 × 60s, 80% amplitude) and centrifuged (15,000 × g, 10 min, 4 °C). The supernatant was discarded, pellet was resuspended in buffer A supplemented with 2 % Triton X-100 and then incubated at 37 °C for 20 min. Following, cells were centrifuged again (15,000 × g, 10 min, 4 °C) and pellet was washed with buffer A and resuspended in denaturing buffer (50mM Tris, 500 mM NaCl, 6M GuHCl). After that, it was incubated at 60 °C overnight. Lysates were sonicated on ice (3

× 60s, 80% amplitude), centrifuged (110,000 × g, 30 min, 16 °C) and then passed through a 0.45-μm filter prior to affinity chromatography. Protein was purified using an AKTA Start FPLC system (GE Healthcare). The lysate was loaded into a HisTrap HP 5mL column (GE Healthcare) previously equilibrated with binding buffer (50 mM Tris, 500 mM NaCl, 50 mM imidazole, 8M urea, pH 8). Protein was eluted from the column with elution buffer (50mM Tris, 0.5M NaCl, 500mM imidazole, 8M urea, pH 8). Next, the purified protein was loaded into a HiPrep 26/10 desalting column (GE Healthcare), and the buffer was exchanged to buffer D (0.01% acetic acid, 0.02% sodium azide) to avoid recombinant TasA polymerization. The protein was kept at −80 °C until used.

Cloning and protein purification of Chitosanase (Csn)

Plasmid pET22b-csn was constructed by amplifying csn gene from B. subtilis NCIB3610. The fragment of DNA containing the open reading frame without signal peptide or codon stop was amplified by PCR and then digested with NdeI and XhoI enzymes and cloned into the plasmid pET22b, cut with the same enzymes. To express and purify recombinant Csn, BL21 (DE3) competent E. coli cells were transformed with plasmid pET22b-csn. Cultures in 100 mL of LB supplemented with 100 μg/mL ampicillin were grown shaking at 37°C until an OD600 of 0.7 was reached. Isopropyl IPTG was added to a final concentration of 1 mM and the cultures were incubated for additional 2 h at 37°C with shaking. Next, cells were collected via centrifugation (7,000 x g, 10 min, 4 °C) and resuspended in buffer A 50 mM Tris, 150mM NaCl, pH 8) supplemented with 0.5 mg/mL lysozyme, 5 mM phenylmethylsulfonyl fluoride (PMSF), and 10x Cell Lysis Reagent (Sigma), and incubated for 1 h at 37 °C. Next, the cells were disrupted via sonication on ice (4 x 60s, 80% amplitude) and passed through a 0.45-μm filter prior to protein purification via affinity chromatography using an AKTA Start FPLC system (GE Healthcare). The lysate was loaded into a HisTrap HP 5 mL column (GE Healthcare) previously equilibrated with binding buffer (50 mM Tris, 500 mM NaCl, 50 mM imidazole, pH 8). Protein was eluted with elution buffer (50 mM Tris, 500 mM NaCl, 500 mM imidazole, pH 8). Next, the purified protein was loaded into a HiPrep 26/10 desalting column (GE Healthcare), and the buffer was exchanged to 20 mM Tris, 50 mM NaCl at

pH 7. Finally, to obtain highly pure desalted protein via size exclusion chromatography, the protein was loaded into a HiPrep 16/60 Sephacryl S-300 HR column (GE Healthcare).

Cloning and protein purification of DEV3pro

The plasmid used for the expression of DEV3pro encodes the protease consisting of the co-factor NS2B and the protease part NS3 being linked via a flexible glycine linker (G4-S-G4), and the N-terminal glutathione S-transferase-tag (GST-tag) with a PreScission protease (GE Healthcare, Little Chaltfront, United Kingdom) cleavage site.

Competent BL21-Gold (DE3) cells were transformed using a pGEX6P1 vector (Addgene Europe, Teddington, United Kingdom). After an overnight culture [100 mL LB medium, 0.1 mg/mL ampicillin, incubated for approximately 13 h at 37 °C with shaking (300 rpm)], 100 mL were transferred to the main culture (2 L SLB medium, 0.1 mg/mL ampicillin, pH 7.5) and incubated to an OD600 of approximately 2.4 at 37 °C under shaking (120 rpm). Subsequently, the temperature was lowered to 17 °C and expression was induced after 30 min using IPTG (final concentration = 0.25 mg/L). After addition of the IPTG solution, the main culture was incubated for another 8 h under shaking (120 rpm), harvesting of cells was performed using an Avanti J-25 centrifuge (Beckman Coulter, Brea, USA) at 10,000 rpm and 4 °C. In the final step, the cell pellets were resuspended in binding buffer (50 mM Tris-Hcl, 150 mM NaCl, 10 mM DTT, pH 7.3) and stored at -80 °C. Each step was monitored by SDS-page. After thawing, the cell suspension was homogenized, the volume increased to approximately 50 mL with binding buffer, upon which cells were disrupted under high pressure (Emulsiflex C5, Avestin, Mannheim, Germany), the obtained suspension was centrifuged using an Avanti J-25 centrifuge (Beckman Coulter, Brea, USA) at 20,000 rpm for 1 h at 4 °C and the resulting supernatant (lysate) was filtered (sterile filter pore size = 0.45 µm, Merck, Darmstadt, Germany,) and finally loaded onto a GSTPrep FF 16/10 column (20 mL, GE Healthcare, Little Chaltfront, UK) being equilibrated with binding buffer (50 mM Tris-HCl, 150 mM NaCl, 10 mM DTT, pH 7.3).The target protein was eluted from the column (0-100% with a flow rate of 3 mL/min over 10 min) using an elution buffer (50 mM Tris-HCl, 150 mM NaCl, 10 mM reduced glutathione, 10 mM DTT, pH 8.0) and the desired fractions were collected, concentrated (15 mL), and

dialyzed (50 mM Tris-HCl, 150 mM NaCl, 1 mM EDTA, 1 mM DTT, pH 7.5) to remove reduced gluthathione. During dialysis, PreScission protease was added at a ratio of 2 : 1 to cleave off the GST-tag. After dialysis, the protein was further purified via a GSTPrep FF 16/10 column (20 mL, GE Healthcare, Little Chaltfront, UK) equilibrated with dialysis buffer. Fractions containing the target protein were collected, rebuffered to the starting buffer (50 mM NaH2PO4, 3 M NaCl, pH 7.5) of the phenyl-Sepharose column (5 mL HiPrep Phenyl HP, GE Healthcare, Little Chaltfront, United Kingdom), and the protein was eluted (1.5 mL/min, 0-100%) from the column with elution buffer (50 mM NaH2PO4, pH 7.5). Protein containing fractions were combined and dialyzed overnight against the buffer (50 mM Tris-HCl, 150 mM NaCl, pH 7.5) of the size exchange column (16/60 Superdex 75, GE Healthcare, Little Chaltfront, United Kingdom), concentrated to approximately 1-2 mL and loaded on the SEC (elution at a flow rate of 1.5 mL/min). In the final step, the collected fractions were re-buffered into crystallization buffer (20 mM Tris-HCl, 150 mM NaCl, 5% glycerol, pH 8.5), concentrated, and the purified protein flash frozen and stored at -80 °C. All steps were monitored via SDS page.

Cloning and protein purification of Pim1

The pLIC-SGC construct containing Pim-1 with a 6xHis tag, TEV cleavage site and an R250G mutation was kindly provided by E. Meggers, Philipps-Universität Marburg. After the mutation back to wild type, the vector was transformed into BL21 (DE3) gold cells. After an overnight expression in SLB media at 291.15 K, cells were harvested by centrifugation and disrupted with a high-pressure homogenizer (Emulsiflex C5, Avestin). The lysate was centrifuged and the supernatant loaded onto a nickel affinity column (HisTrap FF 5 mL, Cytiva). To cleave the 6xHis tag, the eluted protein was then treated with TEV protease for 48 h at 277.15 K. The protein was loaded onto the nickel column and washed out with binding buffer, while the TEV protease remained on the column. Further purification was achieved using anion-exchange chromatography (MonoQ, Cytiva). Samples were collected and concentrated to a total volume of 1.5 mL and then loaded onto a size-exclusion column (HiLoad 26/600 Superdex 75 pg) for final purification. The fractions containing Pim1 were united and concentrated to 8 mg/ml in a

buffer containing 50 mM Hepes (pH 7.0), 250 mM NaCl, 5% glycerol and 5 mM DTT. The protein was kept at −80 °C until needed.

Cloning and protein purification of CutA

The *cutA1* gene of *E. coli* BW25113 was inserted into the expression vector pASK IBA3plus (IBA lifesciences, Göttingen, Germany). This plasmid was selected to fuse a *Strep*-Tactin affinity tag to the C-terminus of the recombinant CutA protein for subsequent protein purification. Therefore, the vector pASK was linearized and amplified via PCR using primers pASK_linear_fw (5`- AGCGCTTGGAGCCACC-3`) and pASK_linear_rev (5`- CATTTTTTGCCCTCGTTATCTAGATTTTTGTC-3´). The *cutA1* sequence was amplified from genomic DNA of *E. coli* BW25113 using the primers cutA1_fw (5`-GTGAAATGAATAGTTCGACAAAAATCTAGATAACGAGGGCAAAAAATGCTTGATGAAAAAAG-3`) and cutA1_rev (5`-GTCGCACAATGTGCGCCATTTTTCACTTCACAGGTCAAGCTTATTATTTTTCGAACTGCG-3`). These primers are constructed in order to add overlaps, homologous to the linear pASK backbone, to the *cutA1*-fragment. All PCRs have been performed using Q5 high fidelity polymerase (NEB, Frankfurt am Main, Germany). After purification of the PCR products, the quality of the DNA was verified by measurement of the absorbance ratio at 260 nm and 280 nm. To assemble both fragments, the plasmid backbone of pASK-IBA3plus and the *cutA1*-fragment were ligated using Gibson assembly (Gibson, 2011). Afterwards, the plasmid was transformed into One Shot® TOP10 electrocompetent *E. coli* cells (Thermo Fisher Scientific, Waltham, Massachusetts) for plasmid preparation. Positive transformants were selected on LB-agar plates containing 100 µg/mL ampicillin. Following purification of the plasmid, the correct insertion of *cutA1* into pASK was verified by Sanger sequencing (LightRun Tube, Eurofins Genomics, Konstanz, Germany). pASK[*cutA1*] was transformed into electrocompetent *E. coli* Lemo21(DE3) (NEB) for protein expression. For the induction of CutA expression, *E. coli* Lemo pASK[*cutA1*] was inoculated in double concentrated lysogeny broth (LB) containing ampicillin (100 µg/mL) and chloramphenicol (34 µg/mL). The cells were incubated at 37°C with 120 rpm shaking until an OD_{600} of 0.5 was reached. Expression of CutA was induced by addition of anhydrotetracycline in a final concentration of 200 µg/L. The cells were incubated at 20°C,

120 rpm shaking for 12-14 hours. Cells were harvested by centrifugation (4000 xg, 12 min, 4°C). The cell pellets were resuspended in lysis buffer (100 mM Tris/HCL, pH8, 150 mM NaCl, 1mM EDTA, pipet tip of Dnase I, Rnase A, lysozyme and proteinase inhibitors) and disrupted by sonication. To remove cell debris, the crude lysate was centrifuged (39 000 xg, 60 min, 4°C) and sterile filtered (0.22 µM filter) prior to affinity chromatography with an ÄKTApurifier system (Cytiva, Marlborough, Massachusetts). For that purpose, a 5mL StrepTrap HP (Cytiva) column was used. The protein was eluted from the column with elution buffer containing 100 mM Tris/HCL pH 8, 150mM NaCl, 1mM EDTA, 5 mM D-Desthiobiotin. After elution, the protein quality was verified by SDS page. The collected fractions were combined and concentrated using Amicon Ultra Centrifugal Filters with 3 kDa cutoff (Merck-Millipore, Burlington, Massachusetts). The protein was rebuffered in dialysis buffer containing Tris/HCL 20 mM pH 7.8 and 150 mM KCl. The concentration of CutA was determined by Bradford assay. The protein was stored at −80 °C until used.

Cloning and protein purification of FtsZ (*S. aureus)*

SaFtsZ was cloned and purified as detailed previously (https://pubmed.ncbi.nlm.nih.gov/32605984/). Briefly, ORF SAOUHSC_01150 of *S. aureus* NCTC 8325 was cloned into vector pET22bΔpelB (https://pubmed.ncbi.nlm.nih.gov/17085695/) using the primers pETftsZsa_F: tttccatggtagaatttgaacaaggatttaatc and pETftsZsa_R: aaactcgagacgtcttgttcttcttgaacg, thereby generating pETftsZsa. For expression and purification of SaFtsZ protein, *E. coli* BL21(DE3) harboring pETftsZsa was grown in LB broth supplemented with ampicillin (40 µg/ml) to an OD_{600} of 0.6. Then, protein expression was induced by the addition of IPTG to a final concentration of 1 mM. Expression cultures were harvested 4 h after induction followed by protein purification steps under native conditions via IMAC using nickel-nitrilotriacetic acid affinity chromatography using standard procedures. The quantity and quality of SaFtsZ was verified by Bradford assay (using bovine serum albumin as a reference), spectrophotometry (Nanodrop Technologies), SDS-PAGE, and GTPase activity assays[15]. The protein was kept at −80 °C until used.

Supplementary Figures

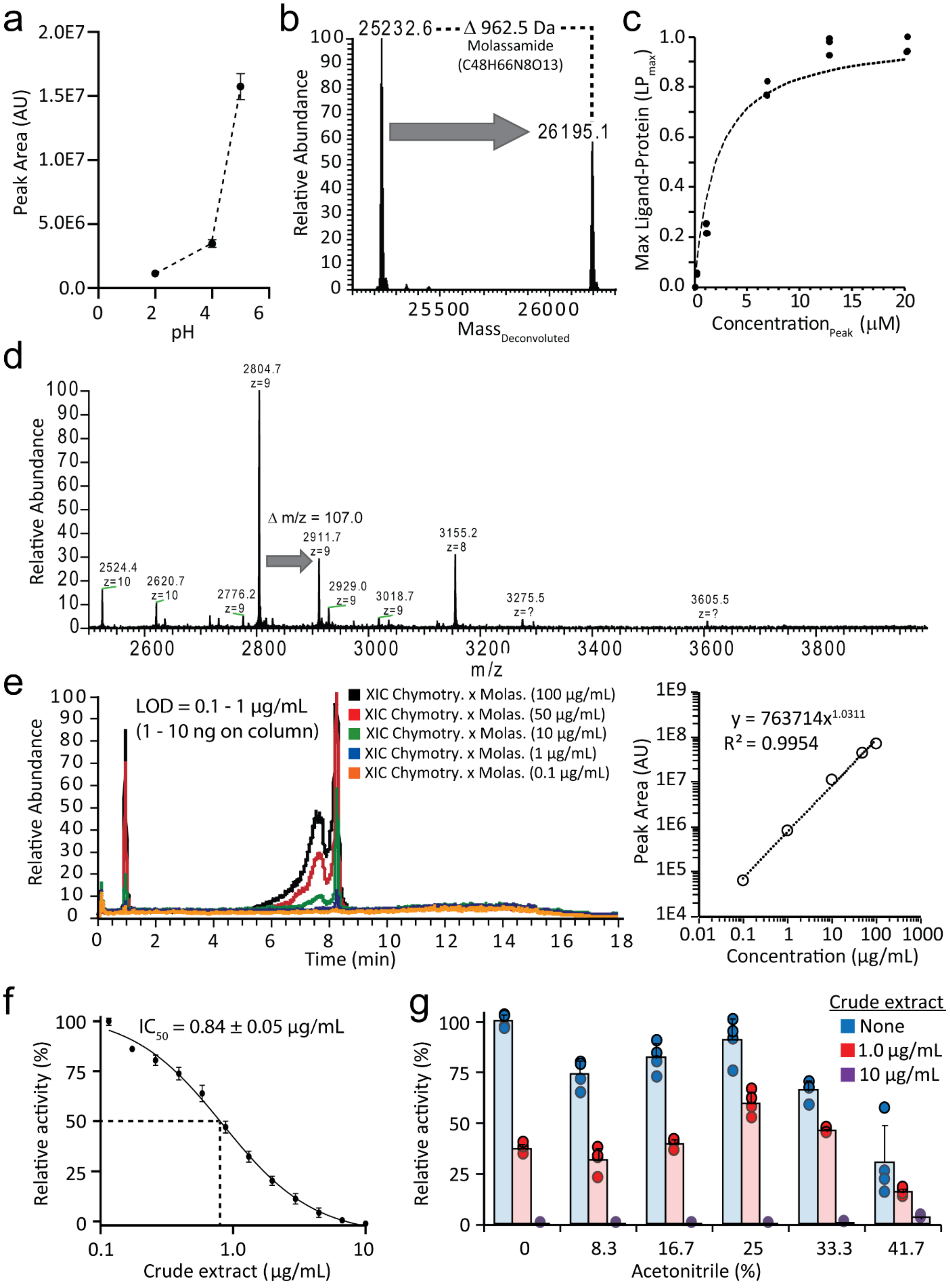

Supplementary Figure **1:** Optimization of native MS conditions a) Integrated peak area of the protein-ligand complex increases in a pH-dependent manner with increasing pH. Data are presented as the mean ± SD, n = 3. (b) Molassamide was screened against chymotrypsin as a proof-of-concept experiment. A Δ*m/z* of 962.5 Da, the difference between unbound chymotrypsin and the chymotrypsin-molassamide complex, corresponds to the *m/z* of molassamide. (c) Concentration-dependent increase in chymotrypsin-molassamide complex, measured by native metabolomics. $\text{Concentration}_{\text{Peak}}$ (µM) refers to the concentration of the ligand. (d) Mass spectrum from chymotrypsin-molassamide complex the from native metabolomics run. (e) The limit of detection (LOD) for the molassamide-chymotrypsin interaction was determined by generating a calibration curve for molassamide and injecting molassamide stock solutions of 0.1 – 100 µg/mL and chymotrypsin. The extracted ion chromatograms (XICs) for the chymotrypsin-molassamide complex are shown. (f) Concentration-dependent inhibition of chymotrypsin activity by 10 µg/mL of cyanobacterial crude extract in 10 mM ammonium acetate pH 4.5. Data are presented as the mean ± SD, n = 3. (g) Relative chymotrypsin activity of cyanobacterial crude extract (1 µg/mL and 10 µg/mL) in native MS conditions (10 mM ammonium acetate pH 4.5 plus increasing concentrations of acetonitrile) compared with no addition of crude extract. Data are presented as the mean ± SD, n = 4. Source data are provided as a Source Data file.

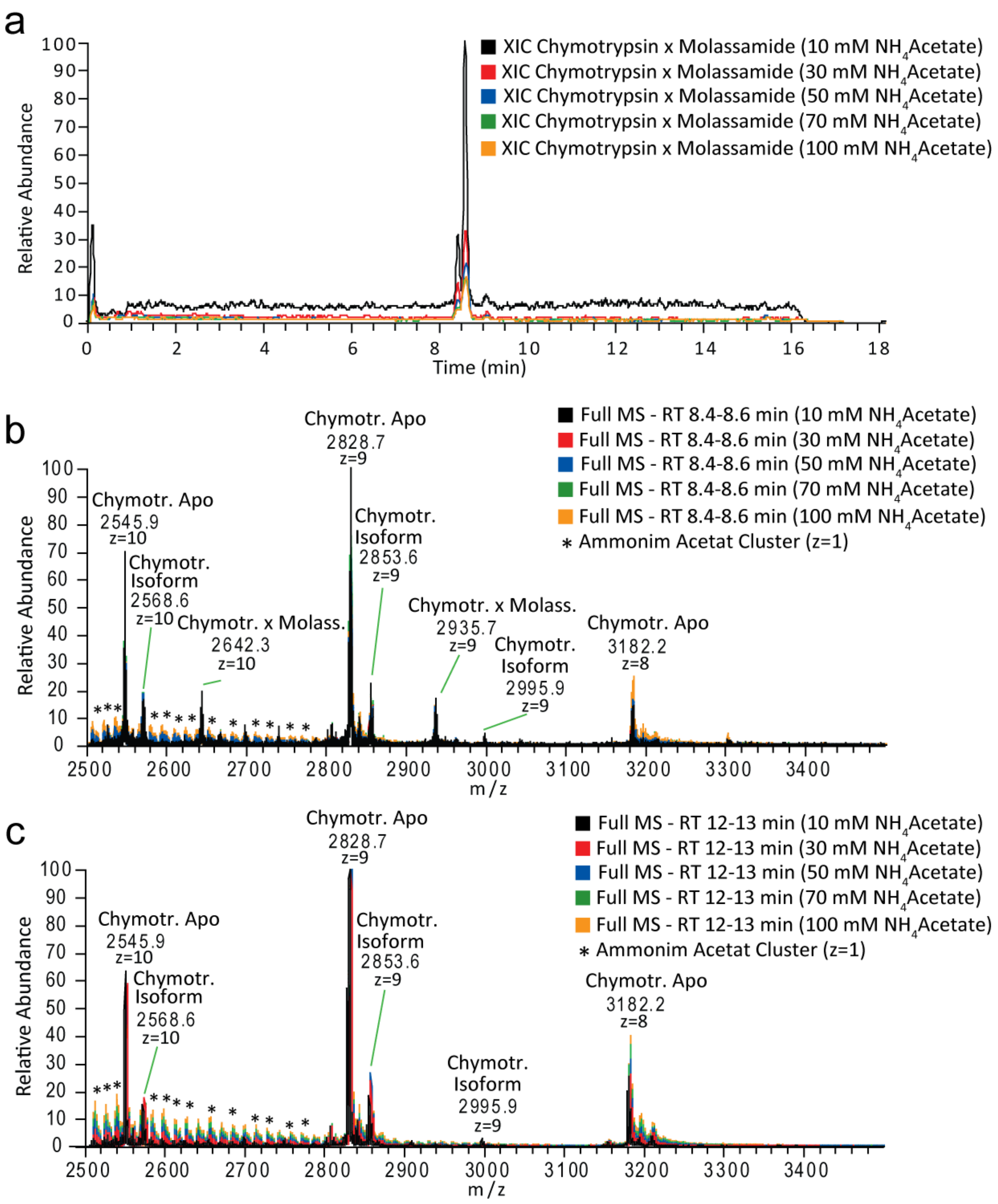

Supplementary Figure 2: Optimization of NH_4Acetate buffer concentration. Native LC-MS analysis with molassamide and chymotrypsin were performed under different make-up buffer concentrations (10-100 mM final) to determine optimal binding/ MS sensitivity. a) shows the extracted ion chromatograms of the chymotrypsin x molassamide complex. b) shows the average MS spectrum (8.4-8.6 min) of the chymotrypsin x molassamide complex. c) displays a reference MS spectrum of chymotrypsin at the max. ACN (40%) concentration at the end of the gradient.

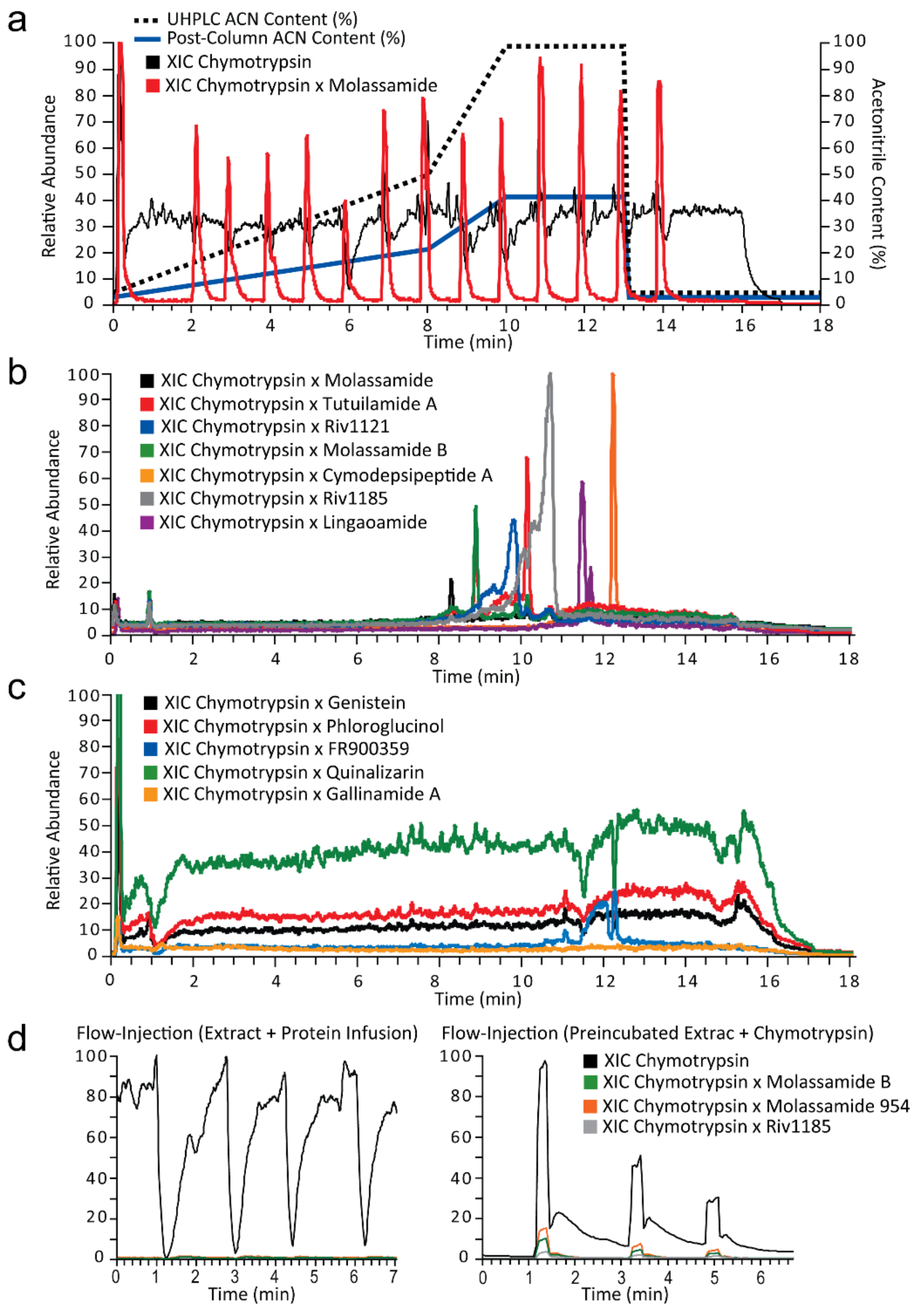

Supplementary Figure 3: Selectivity tests and comparison of native metabolomics with direct infusion. (a) To test the variability of the changing acetonitrile concentration during the LC separation, we removed the UHPLC column and performed a series of flow injections over the full gradient. The XIC of the molassamide bound chymotrypsin reveals similar signal responses throughout the gradient (5-99% ACN on column) (b) Native MS run of positive controls versus alpha-chymotrypsin. Binding of all positive controls to chymotrypsin can be detected in the expected mass range by extracted ion chromatograms (XICs). (c) Native MS run of negative controls genistein, phloroglucinol, FR900359, quinalizarin, gallinamide A versus alpha-chymotrypsin. The drop in detected protein mass is clearly visible due to the co-elution of the small molecules, but no binding of the negative controls to chymotrypsin can be detected in the expected mass range. (d) Left panel: Flow injection of crude methanolic *Rivularia* sp. extract and simultaneous chymotrypsin infusion. Right panel: Incubation (30 min) of chymotrypsin with the crude extract prior to flow injection. XICs of detected chymotrypsin bound to rivulariapeptolide 1185 and molassamides.

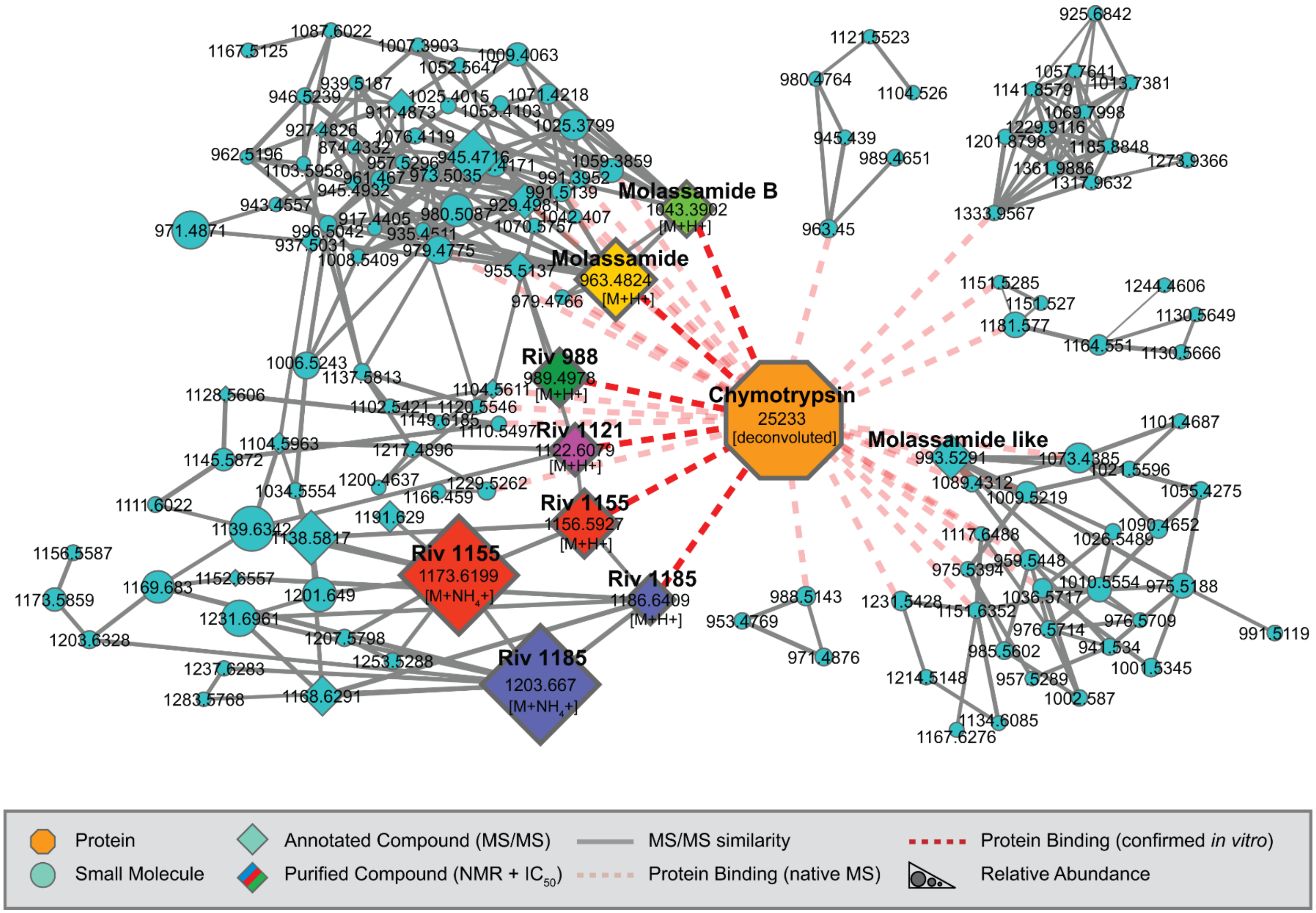

Supplementary Figure 4: Correlation molecular network of chymotrypsin (orange) and putative small molecule inhibitors binders (teal = unknown binders, green, yellow, purple, red, pink = in this study isolated and fully characterized small molecules) by native MS.

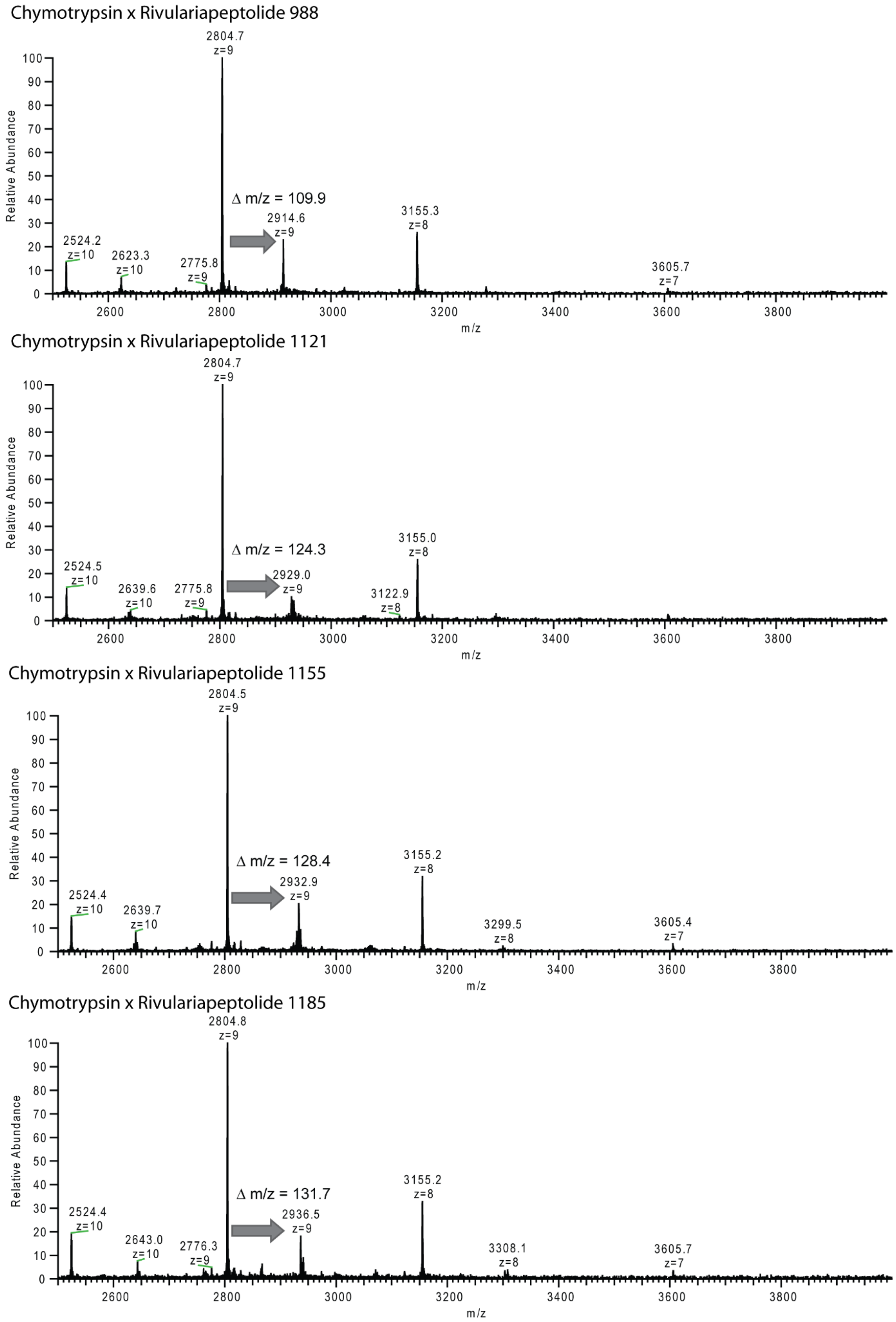

Supplementary Figure 5: Mass spectra from chymotrypsin-rivulariapeptolide complexes from native metabolomics runs.

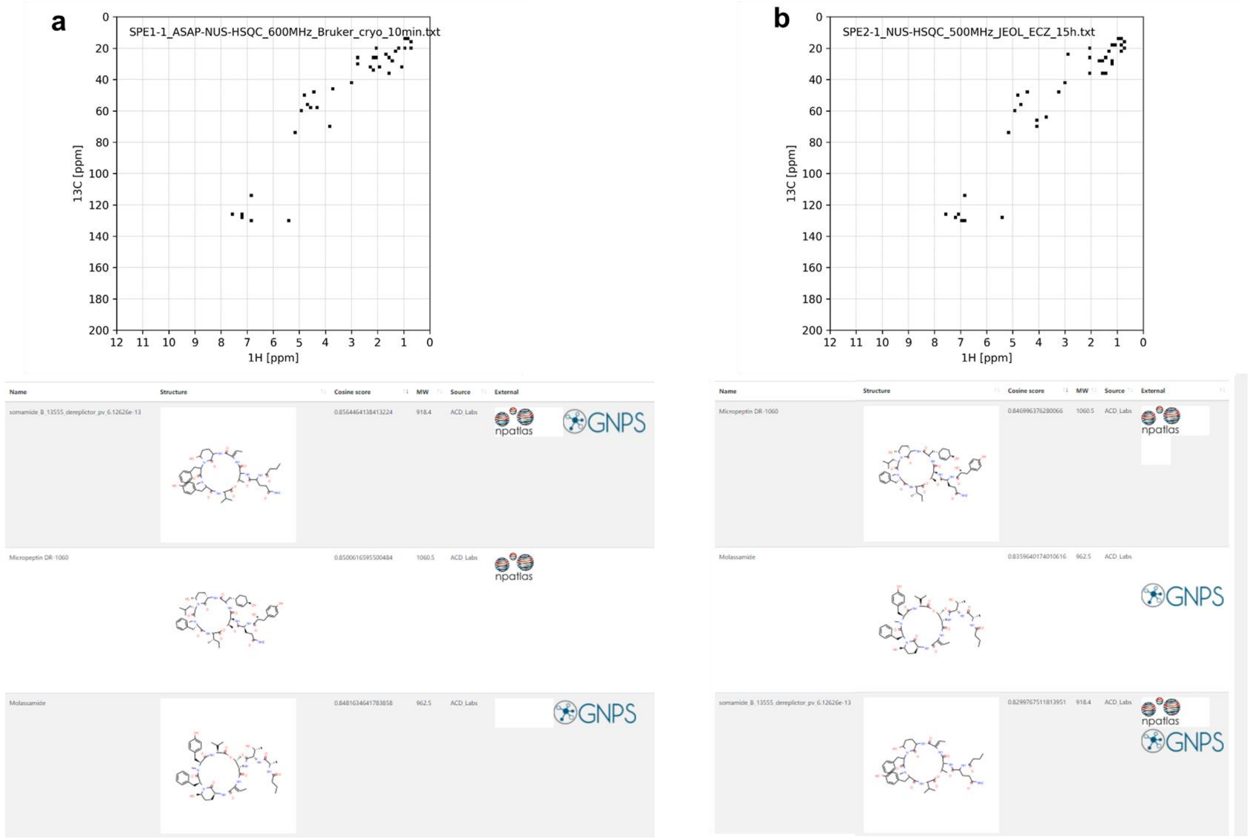

Supplementary Figure 6: SMART 2.1 reproducibility test and mixture analysis. The crude extract was divided into two identical halves. One half was separated by solid phase extraction into four fractions of decreasing polarity (1-1, 1-2, 1-3, 1-4). The second half of the crude extract was fractionated in an identical fashion as described for fraction 1-1, yielding the equivalent SPE fraction 2-1. (a) Top 3 SMART 2.1 predicted compounds for SPE fraction 1-1. NMR acquisition was performed on 1 mg material in a Bruker 1.7 mm TCI MicroCryoProbe (599.10 MHz) to obtain the correlations of the major components of that fraction in 13 minutes. (b) Top 3 SMART 2.1 predicted compounds from SPE fraction 1-1. NMR acquisition was performed on 1 mg of fraction 2-1 on a JEOL 500 MHz ECZ instrument in 15 hours. For complete results refer to https://smart.ucsd.edu/resultclassic?task=4bf3d8c3-3a96-419e-a5f5-416e30acc56b, (fraction 1-1, Bruker 1.7 mm TCI MicroCryoProbe (599.10 MHz) or https://smart.ucsd.edu/resultclassic?task=a44d9275-dc58-49dd-9320-5c620fb89d3e (fraction 2-1, JEOL 500MHz ECZ instrument), respectively.

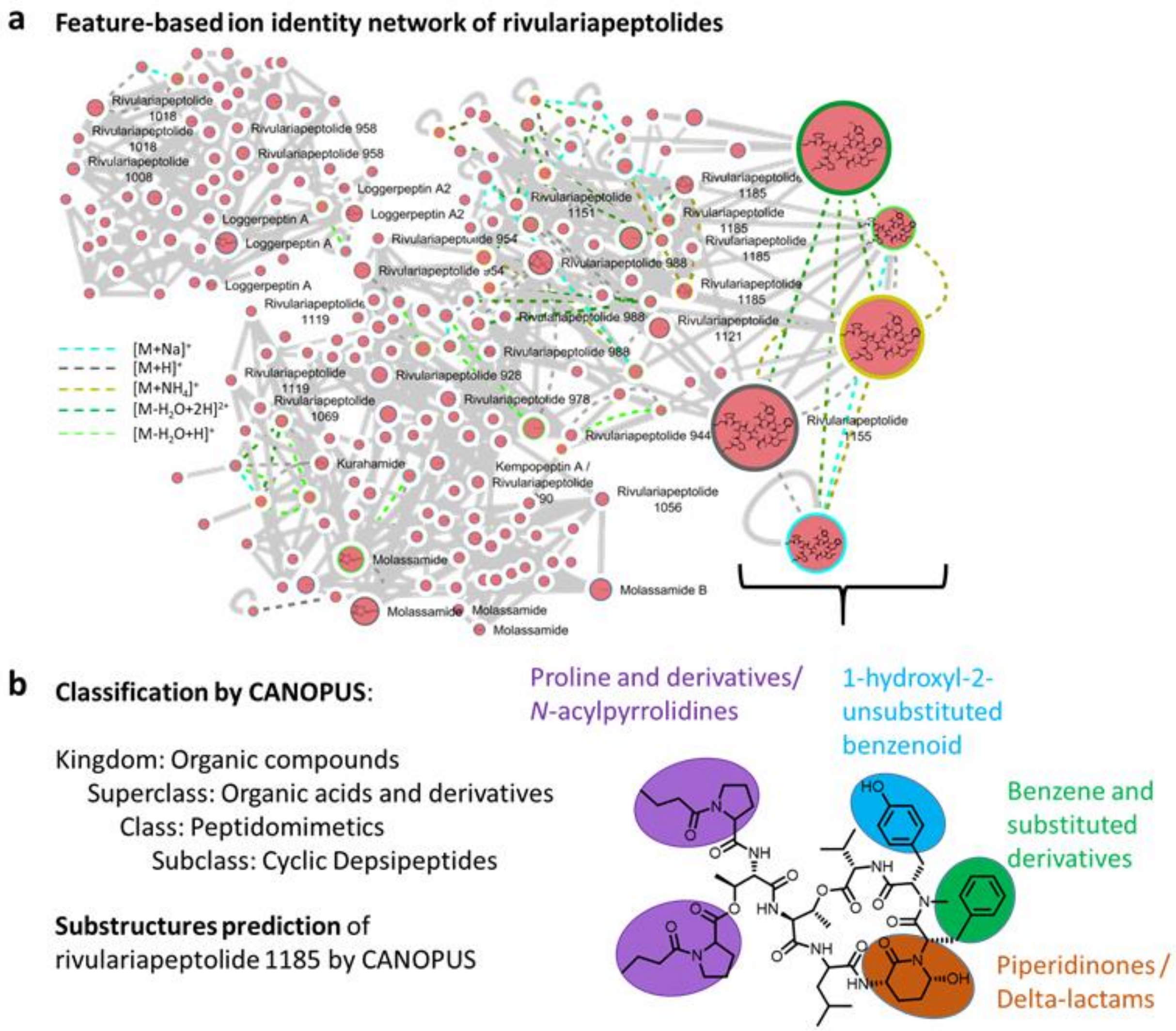

Supplementary Figure 7: Tandem MS analysis of rivulariapeptolides (a) Feature-based ion identity networking and (b) classification, substructure analysis of rivulariapeptolides and other Ahp-cyclodepsipeptides by CANOPUS.

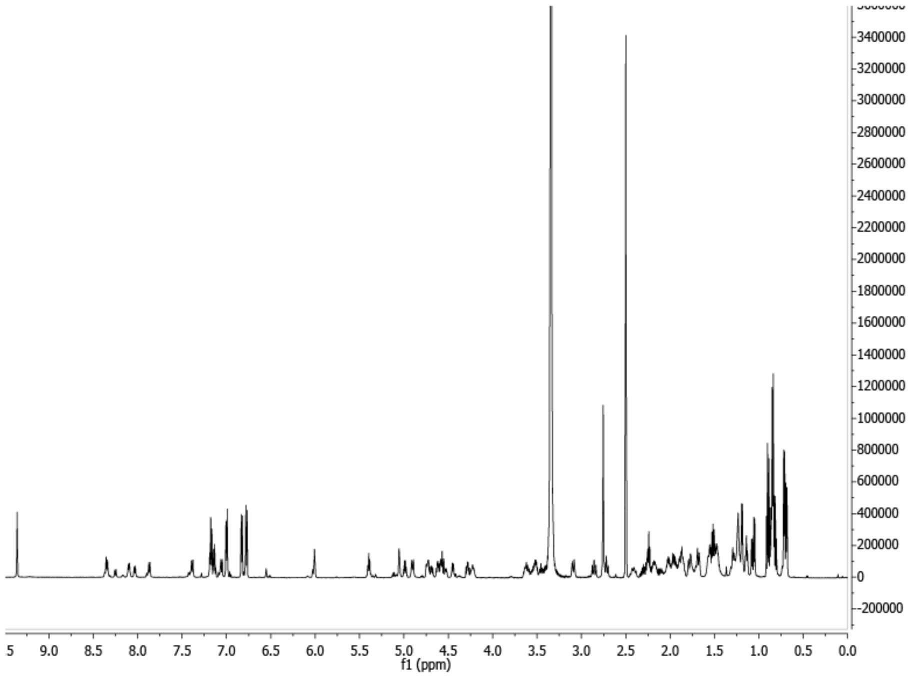

Supplementary Figure 8**:** ^{1}H NMR spectrum of rivulariapeptolide 1185 in DMSO-*d6*, 600 MHz.

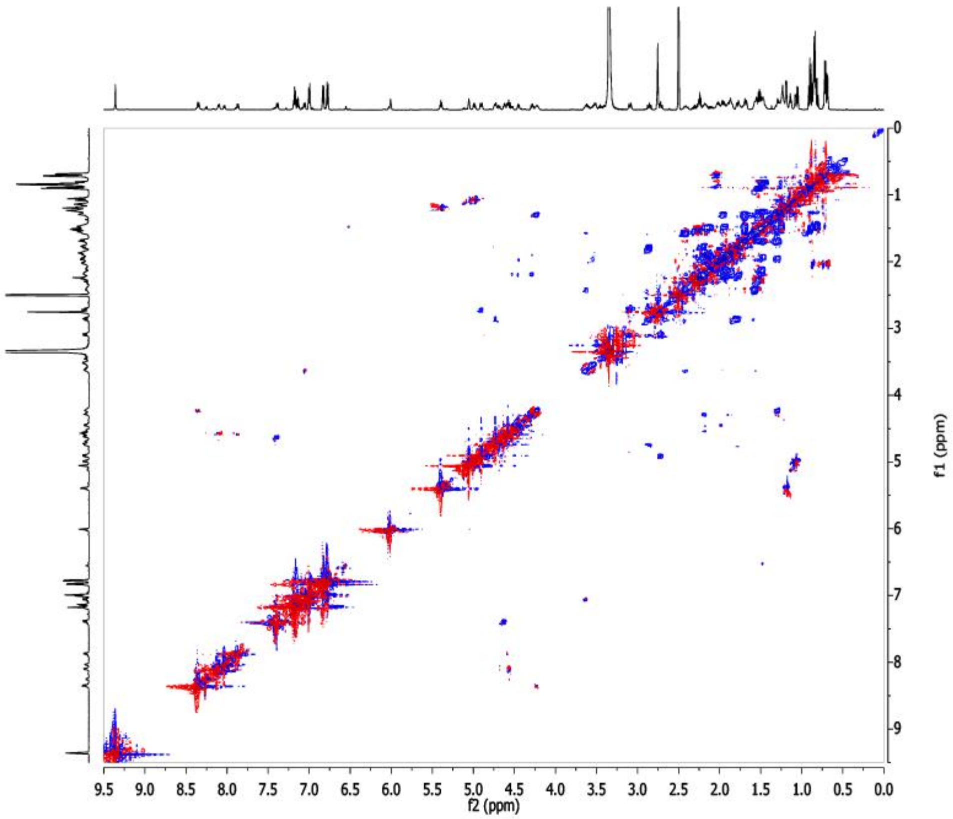

Supplementary Figure 9**:** ^{1}H-^{1}H COSY_CLIP spectrum of rivulariapeptolide 1185 in DMSO-*d6*, 600 MHz.

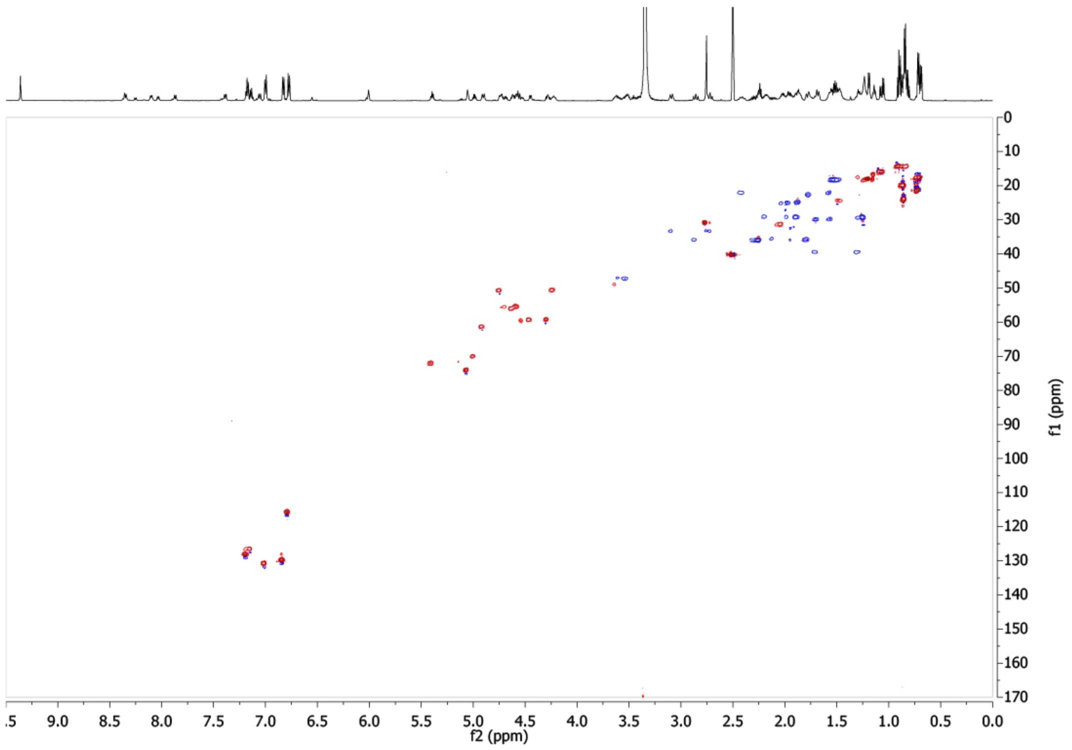

Supplementary Figure 10**:** ^{1}H-^{13}C HSQC spectrum of rivulariapeptolide 1185 in DMSO-*d6*, 600 MHz.

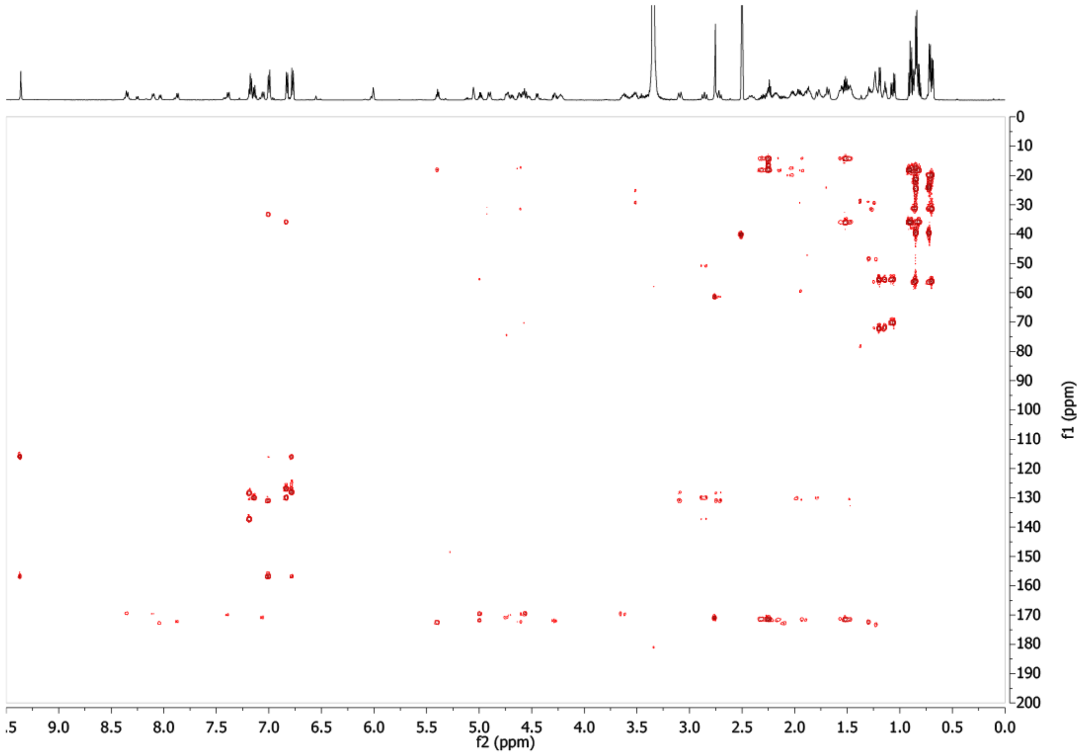

Supplementary Figure 11**:** ^{1}H-^{13}C HMBC spectrum of rivulariapeptolide 1185 in DMSO-*d6*, 600 MHz.

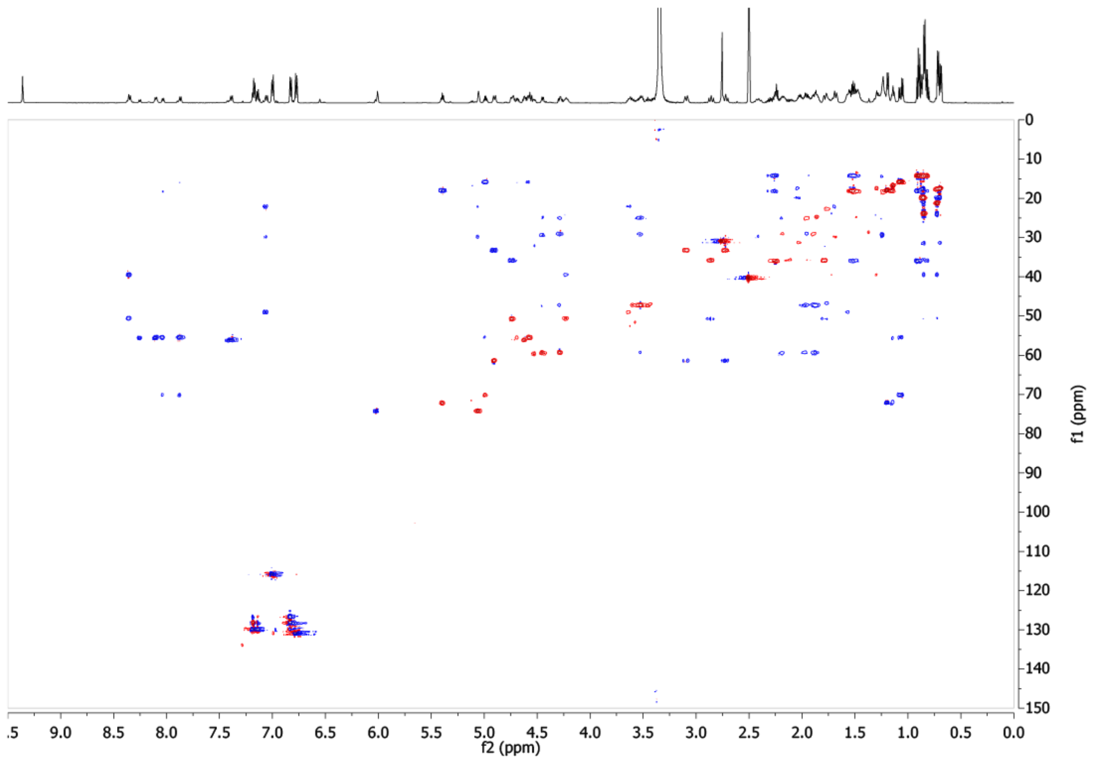

Supplementary Figure 12**:** ^{1}H-^{13}C HSQC-TOCSY spectrum of rivulariapeptolide 1185 in DMSO-*d6*, 600 MHz.

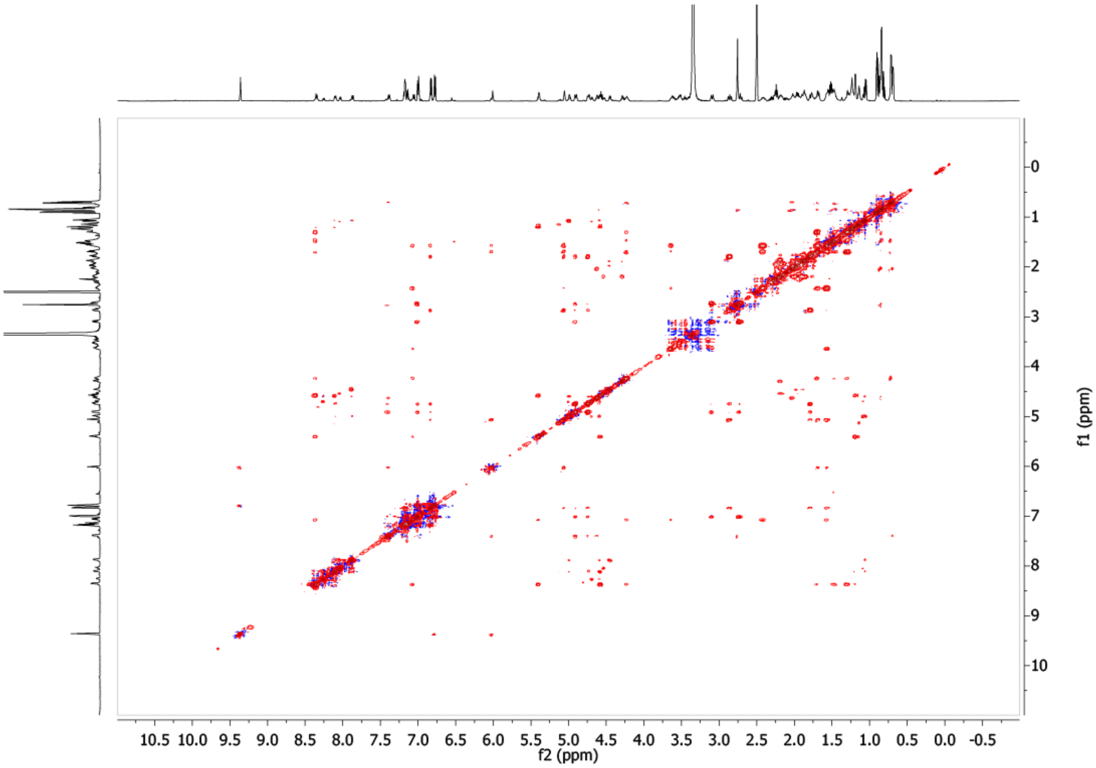

Supplementary Figure 13**:** ^{1}H-^{1}H NOESY spectrum of rivulariapeptolide 1185 in DMSO-*d6*, 600 MHz.

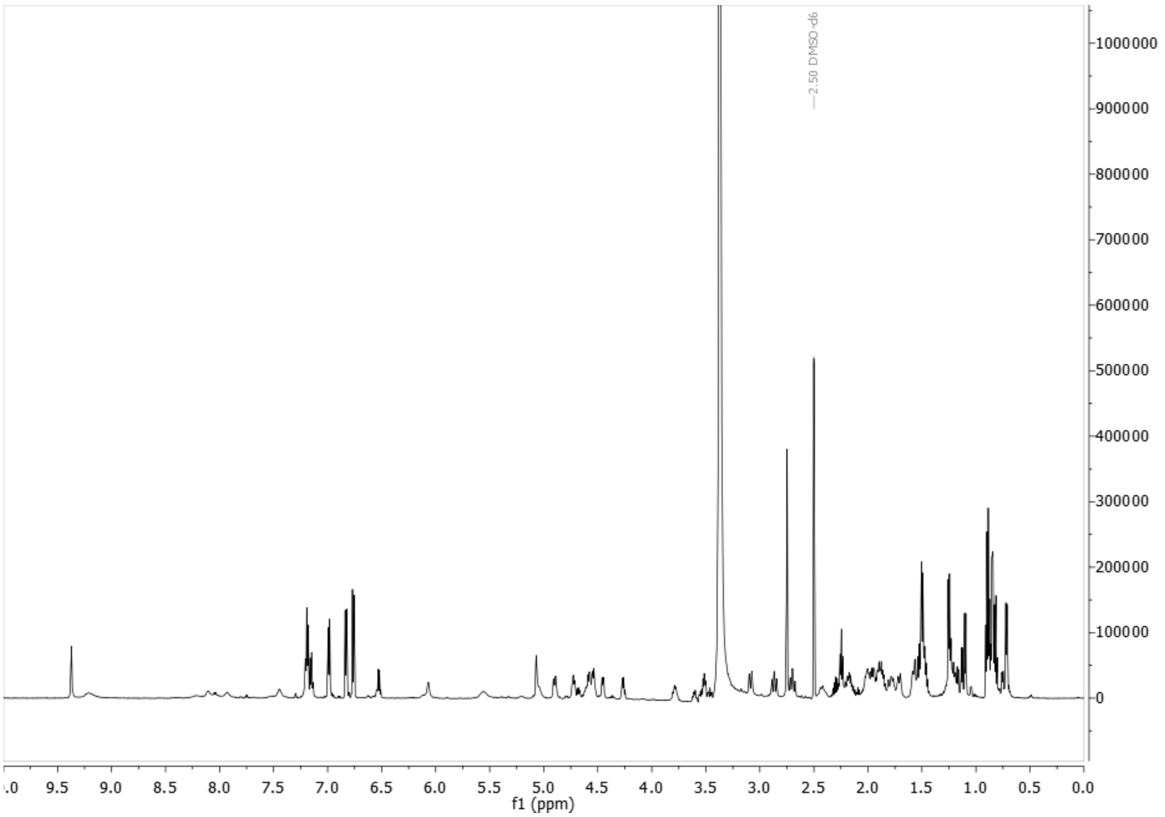

Supplementary Figure 14**:** ^{1}H NMR spectrum of rivulariapeptolide 1155 in DMSO-*d6*, 600 MHz.

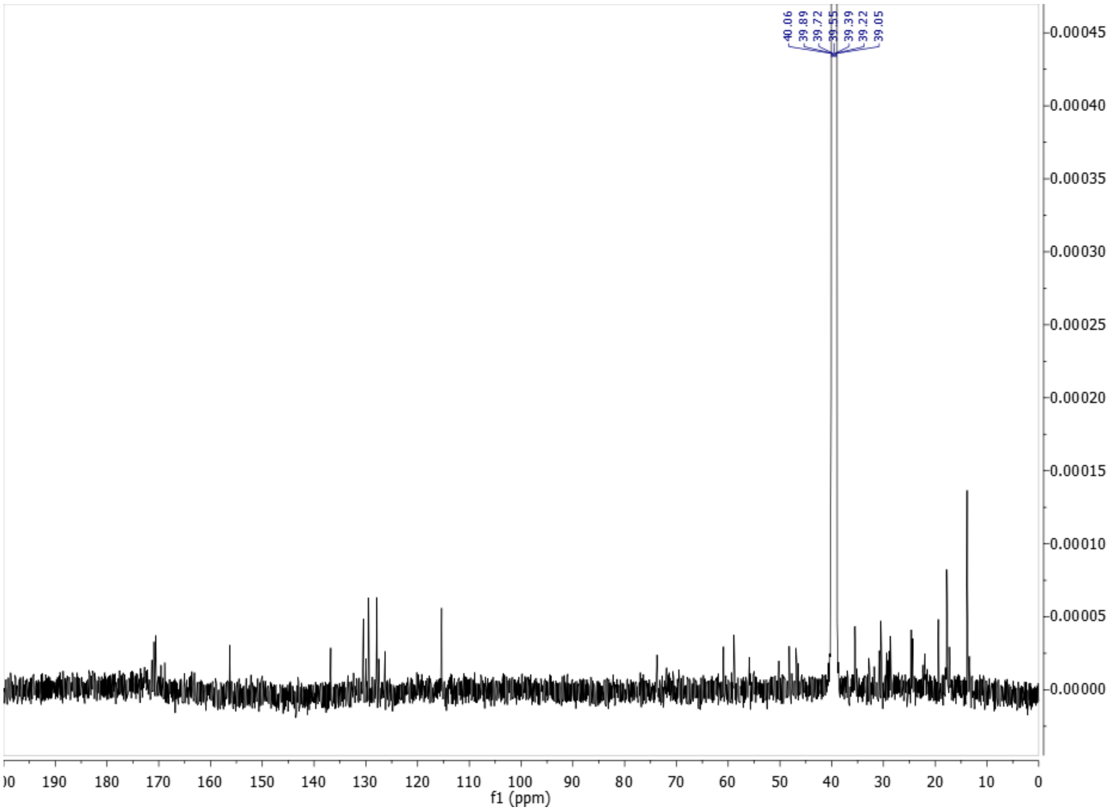

Supplementary Figure 15**:** ^{13}C NMR spectrum of rivulariapeptolide 1155 in DMSO-*d6*, 125 MHz.

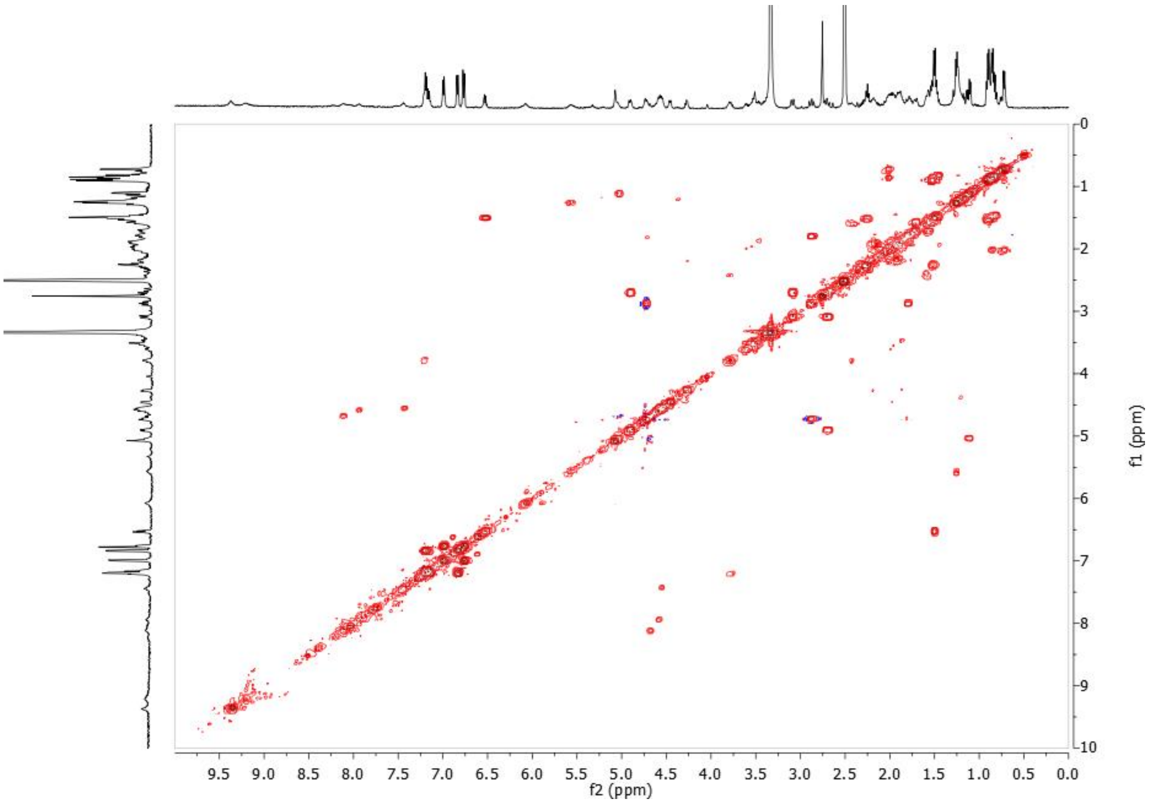

Supplementary Figure 16: ^{1}H-^{1}H COSY spectrum of rivulariapeptolide 1155 in DMSO-*d6*, 500 MHz.

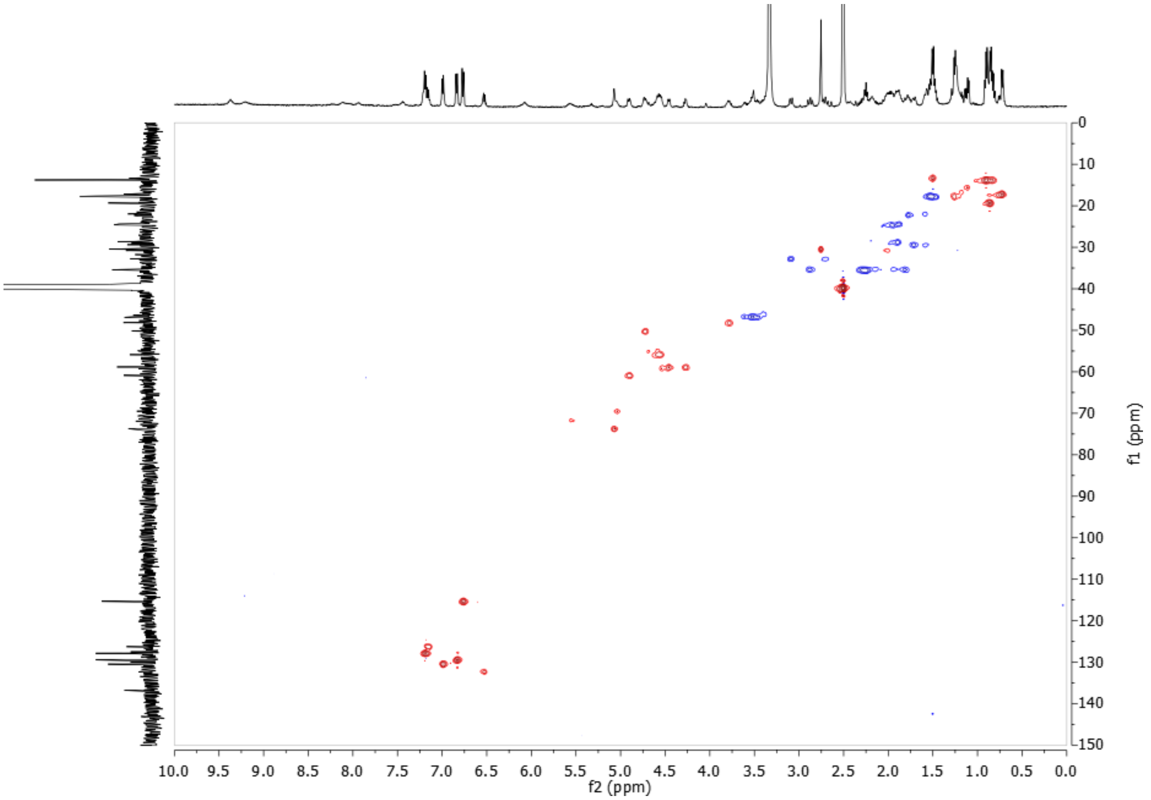

Supplementary Figure 17: ^{1}H-^{13}C HSQC spectrum of rivulariapeptolide 1155 in DMSO-*d6*, 500 MHz.

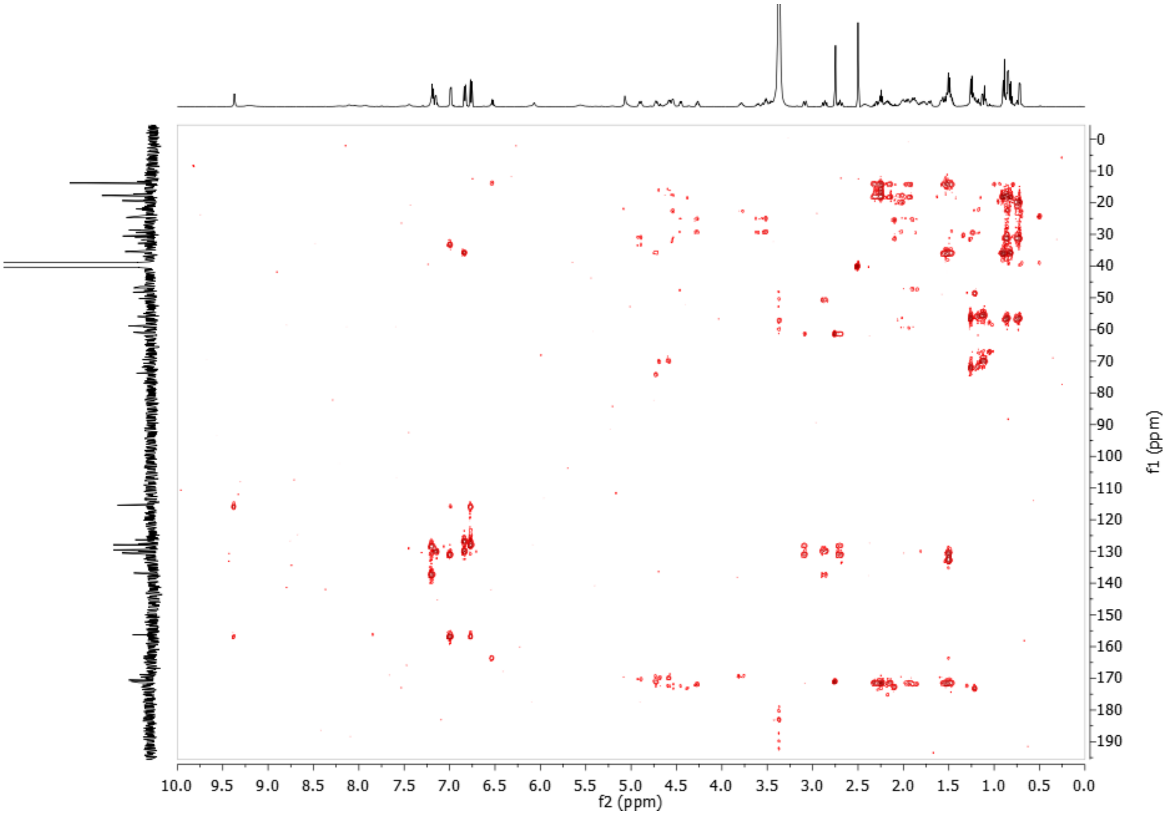

Supplementary Figure 18: ^{1}H-^{13}C HMBC spectrum of rivulariapeptolide 1155 in DMSO-*d6*, 600 MHz.

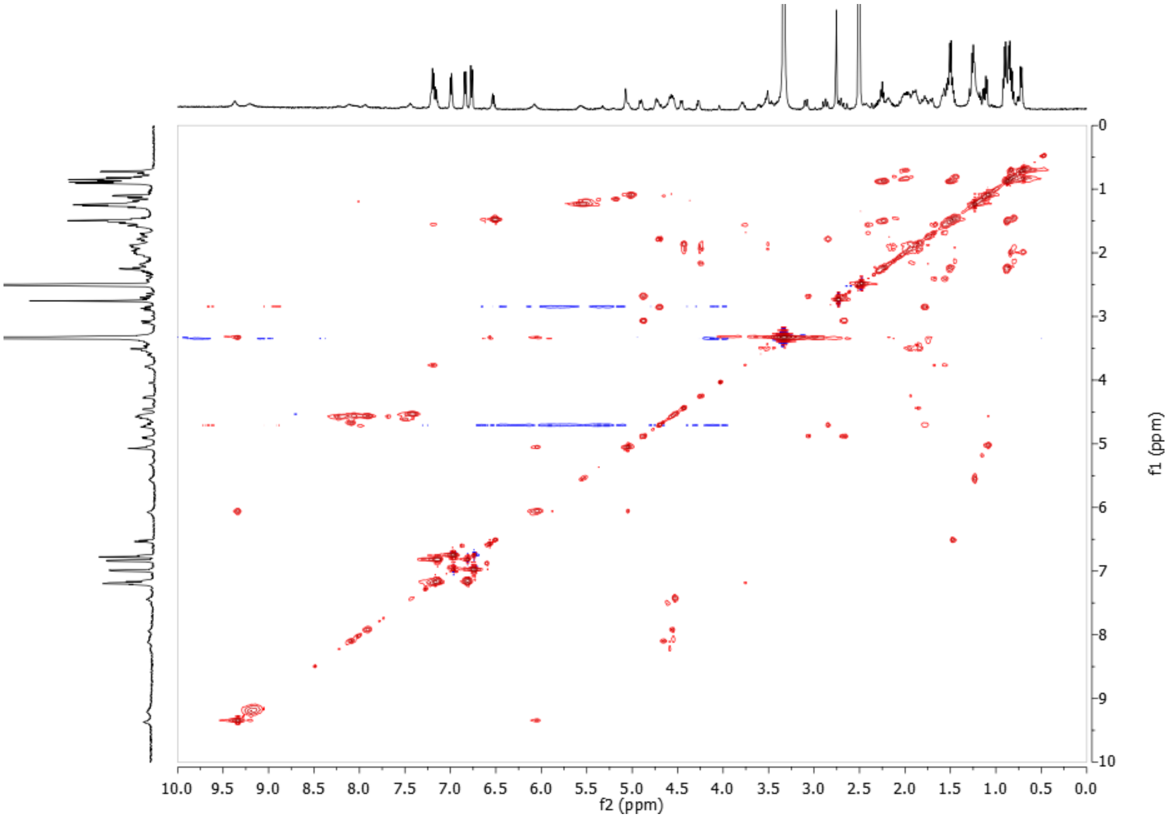

Supplementary Figure 19: ^{1}H-^{1}H TOCSY spectrum of rivulariapeptolide 1155 in DMSO-*d6*, 500 MHz.

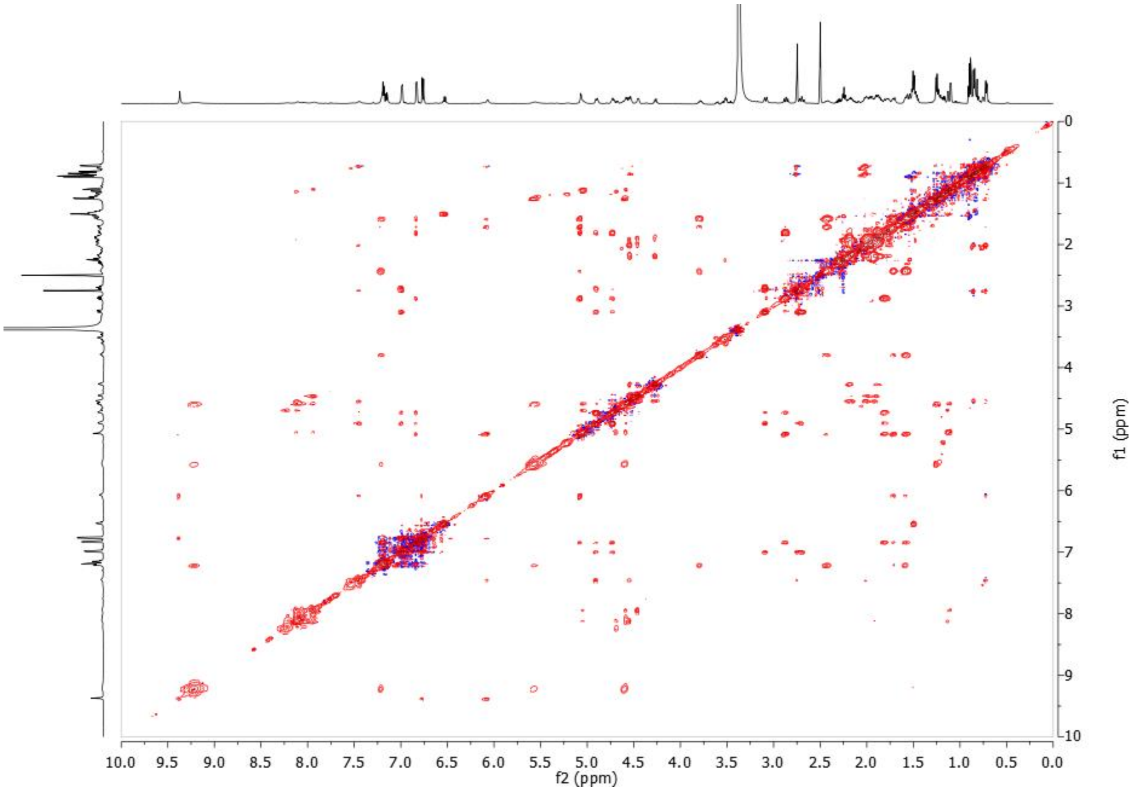

Supplementary Figure 20**:** ^{1}H-^{1}H NOESY spectrum of rivulariapeptolide 1155 in DMSO-*d6*, 600 MHz.

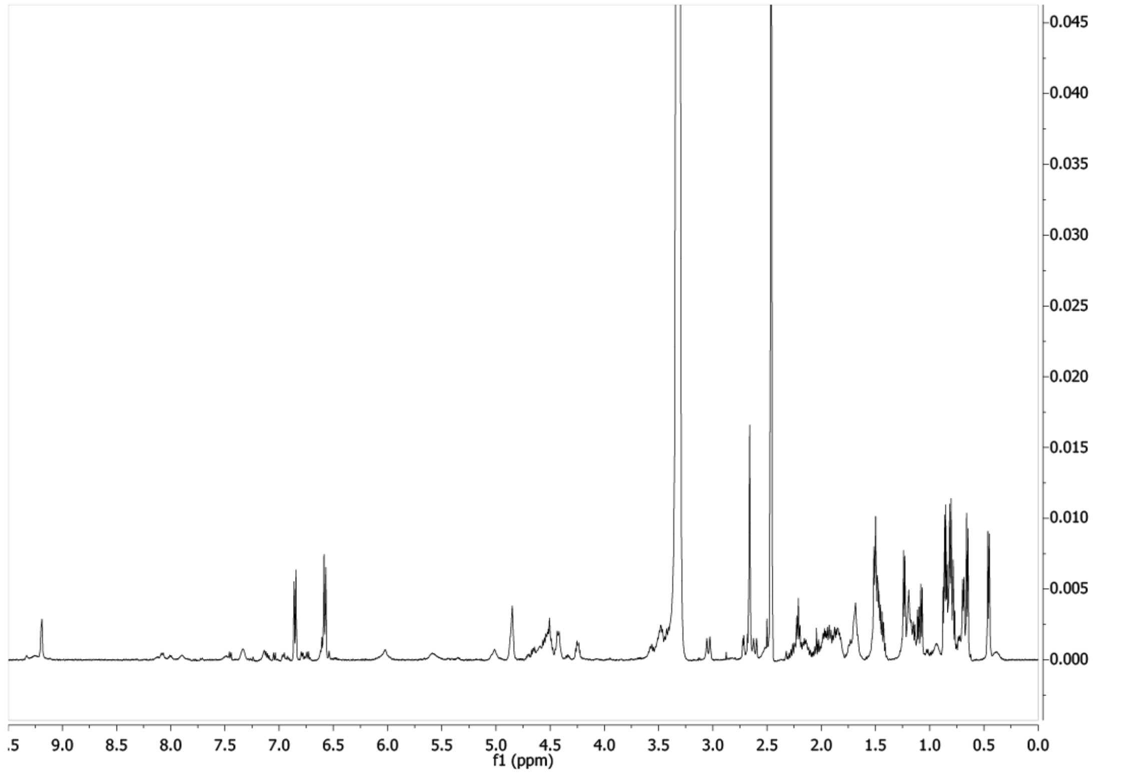

Supplementary Figure 21: ^{1}H NMR spectrum of rivulariapeptolide 1121 in DMSO-*d6*, 600 MHz.

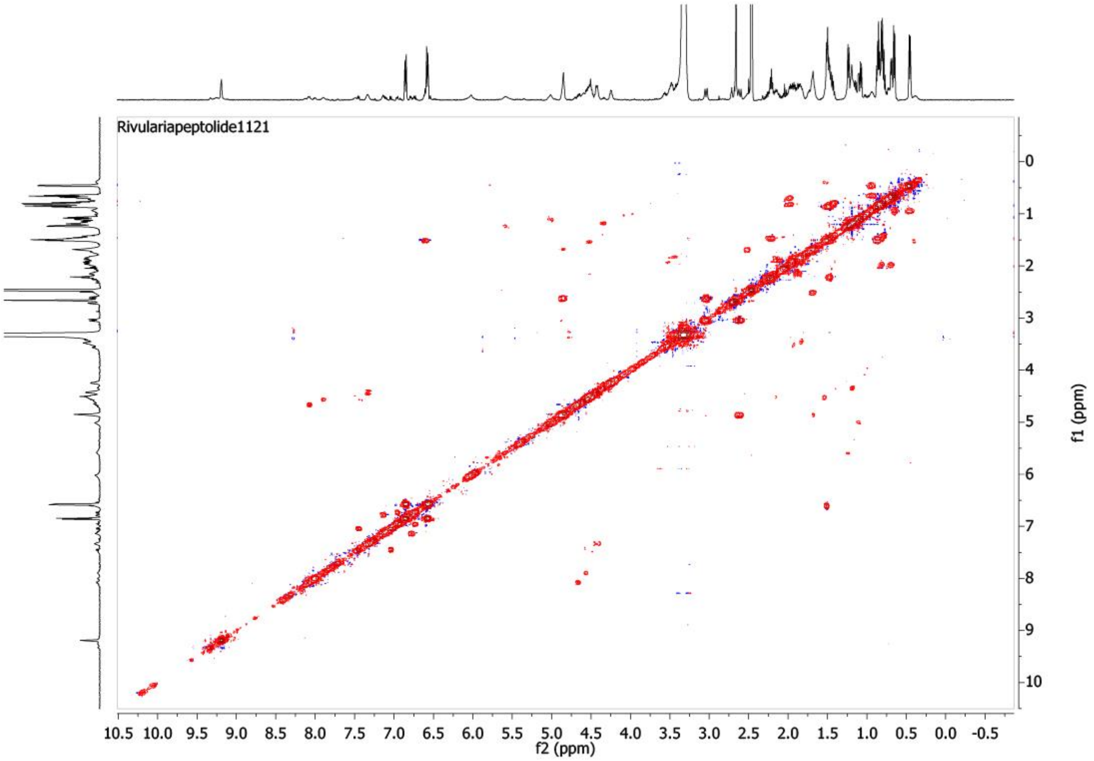

Supplementary Figure 22**:** ^{1}H-^{1}H COSY spectrum of rivulariapeptolide 1121 in DMSO-*d6*, 600 MHz.

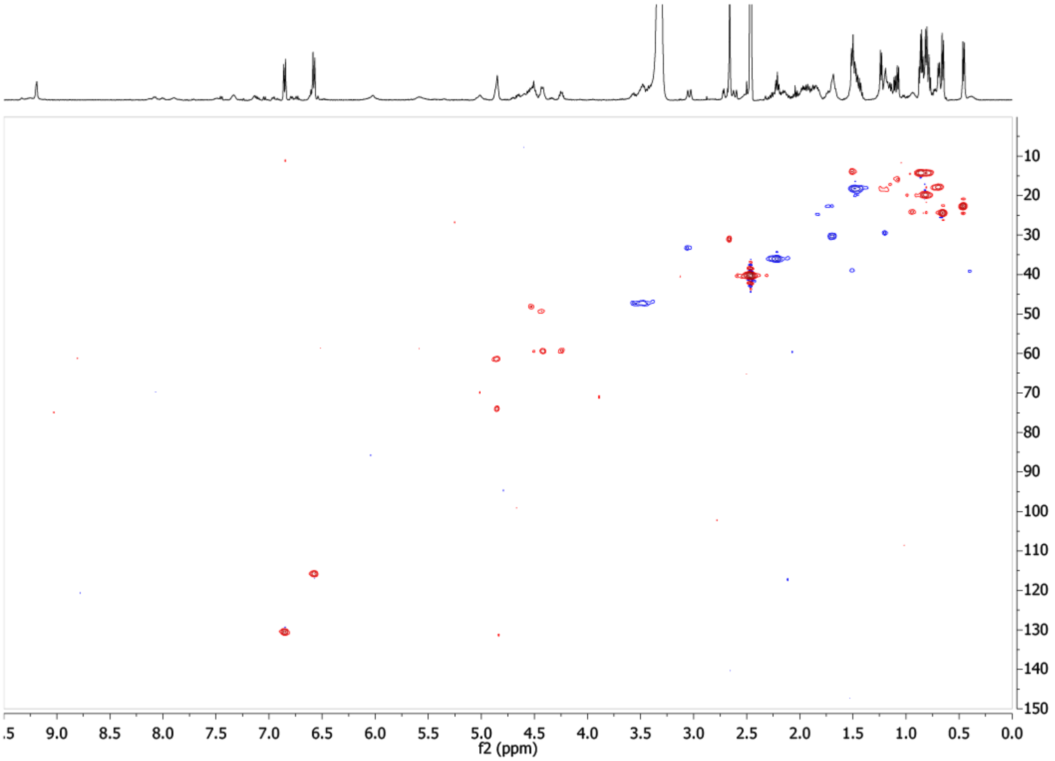

Supplementary Figure 23**:** ^{1}H-^{13}C HSQC spectrum of rivulariapeptolide 1121 in DMSO-*d6*, 600 MHz.

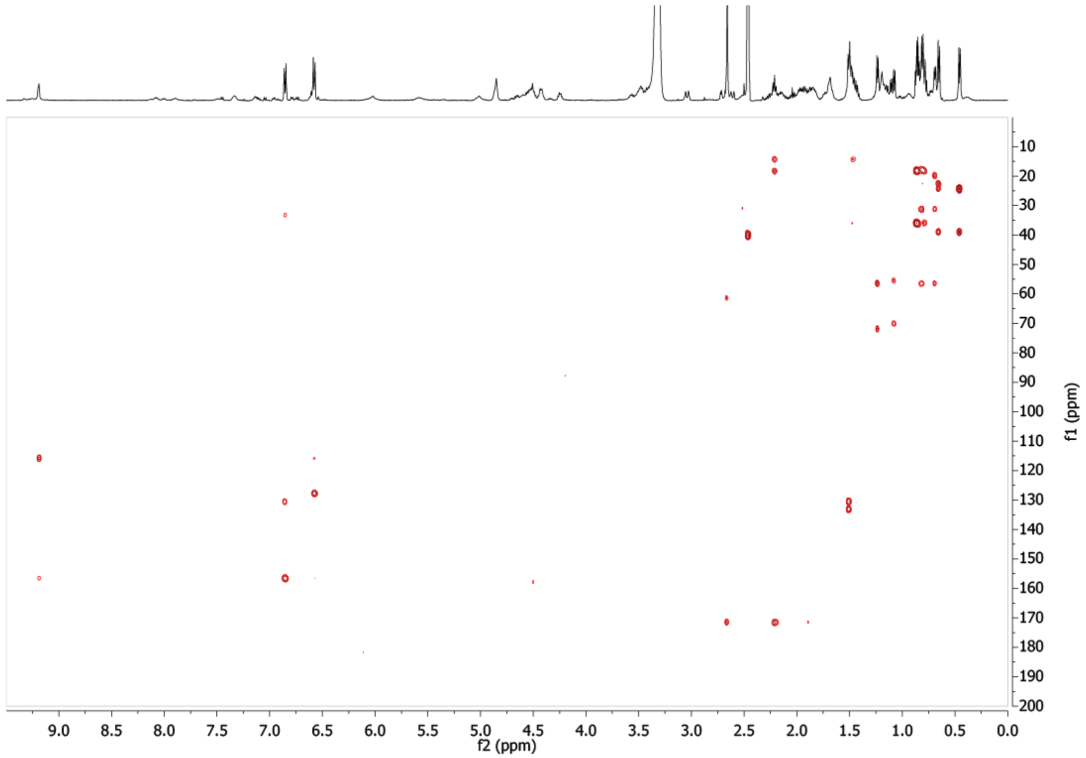

Supplementary Figure 24: ^{1}H-^{13}C HMBC spectrum of rivulariapeptolide 1121 in DMSO-*d6*, 600 MHz.

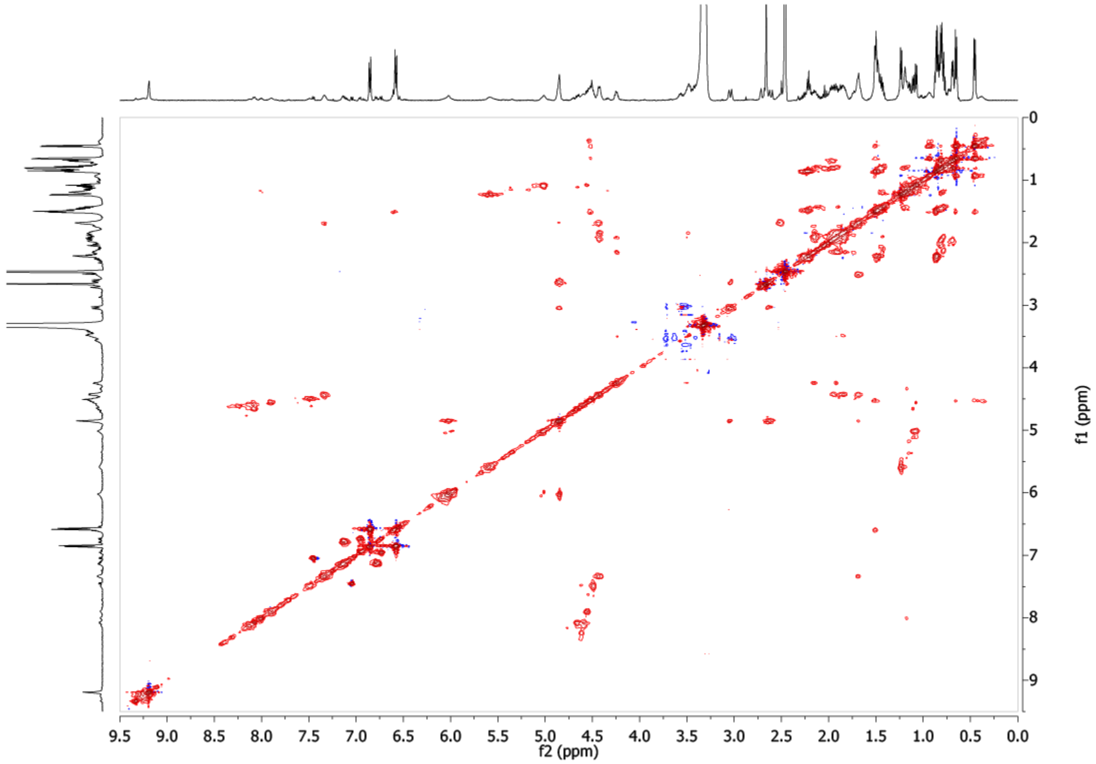

Supplementary Figure 25: ^{1}H-^{1}H TOCSY spectrum of rivulariapeptolide 1121 in DMSO-*d6*, 600 MHz.

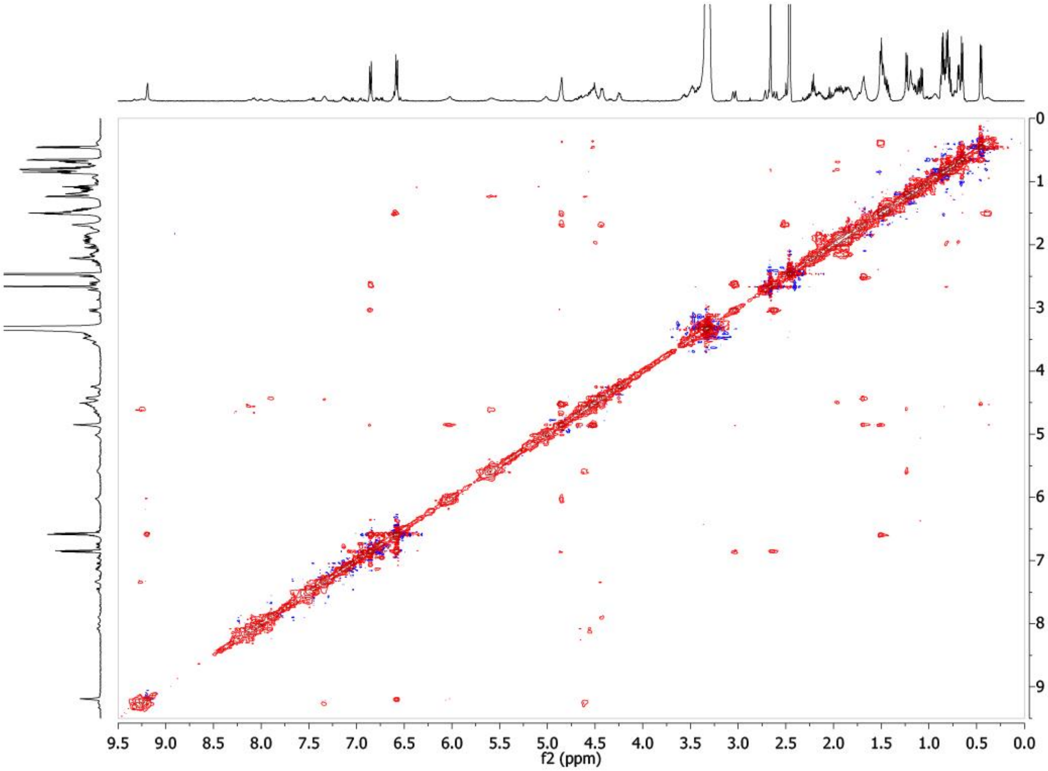

Supplementary Figure 26**:** ^{1}H-^{1}H NOESY spectrum of rivulariapeptolide 1121 in DMSO-*d6*, 600 MHz.

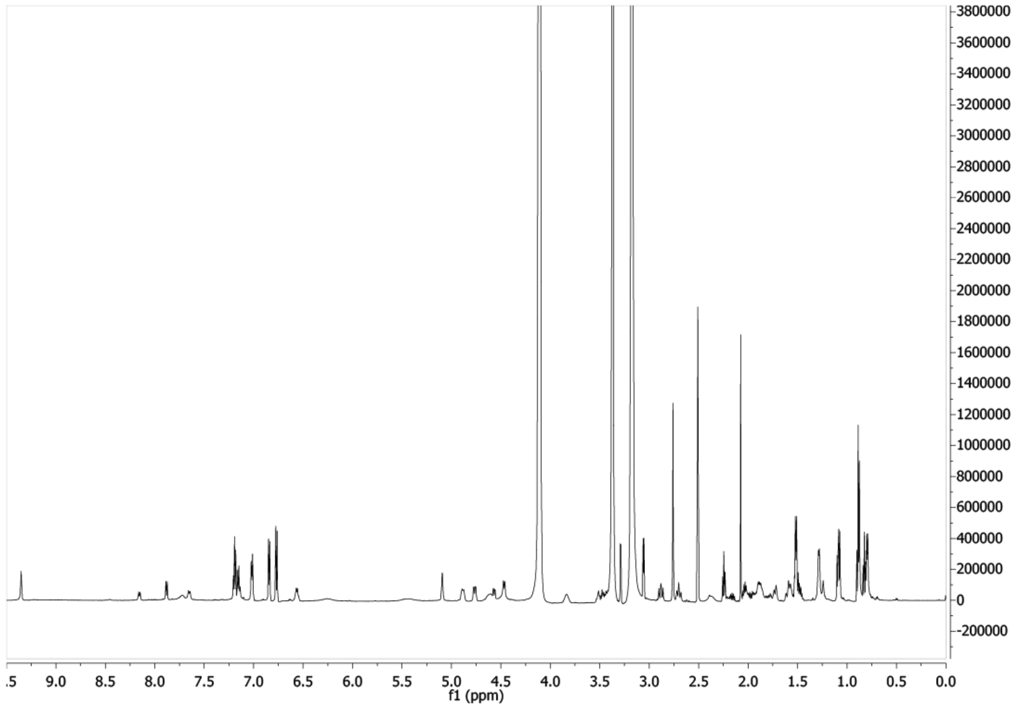

Supplementary Figure 27: ^{1}H NMR spectrum of rivulariapeptolide 988 in DMSO-*d6*, 600 MHz.

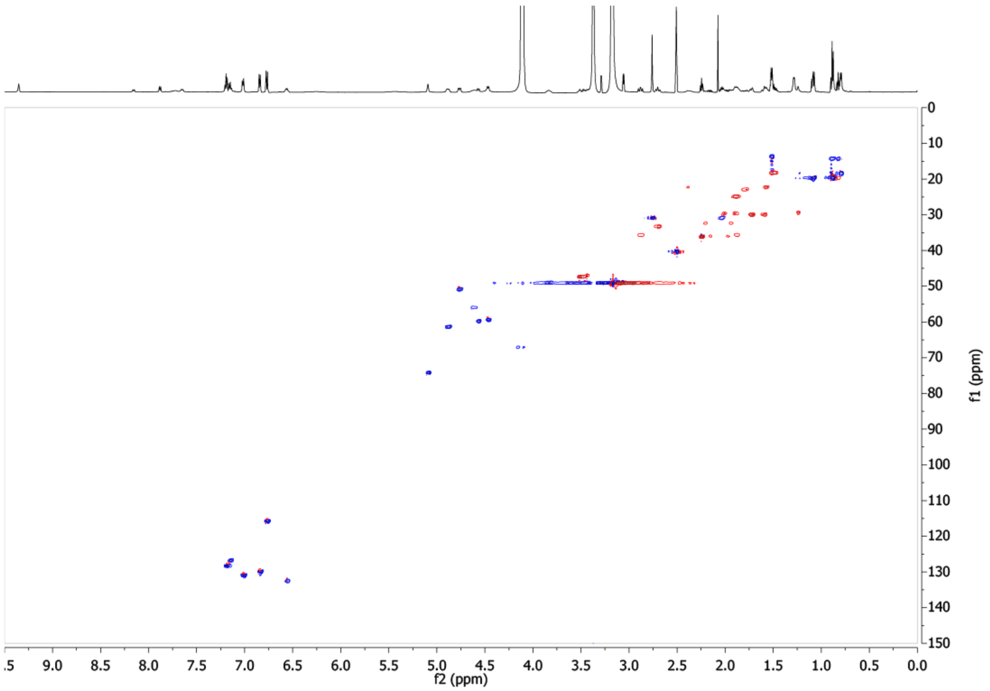

Supplementary Figure 28: ^{1}H-^{13}C HSQC spectrum of rivulariapeptolide 988 in DMSO-*d6*, 600 MHz.

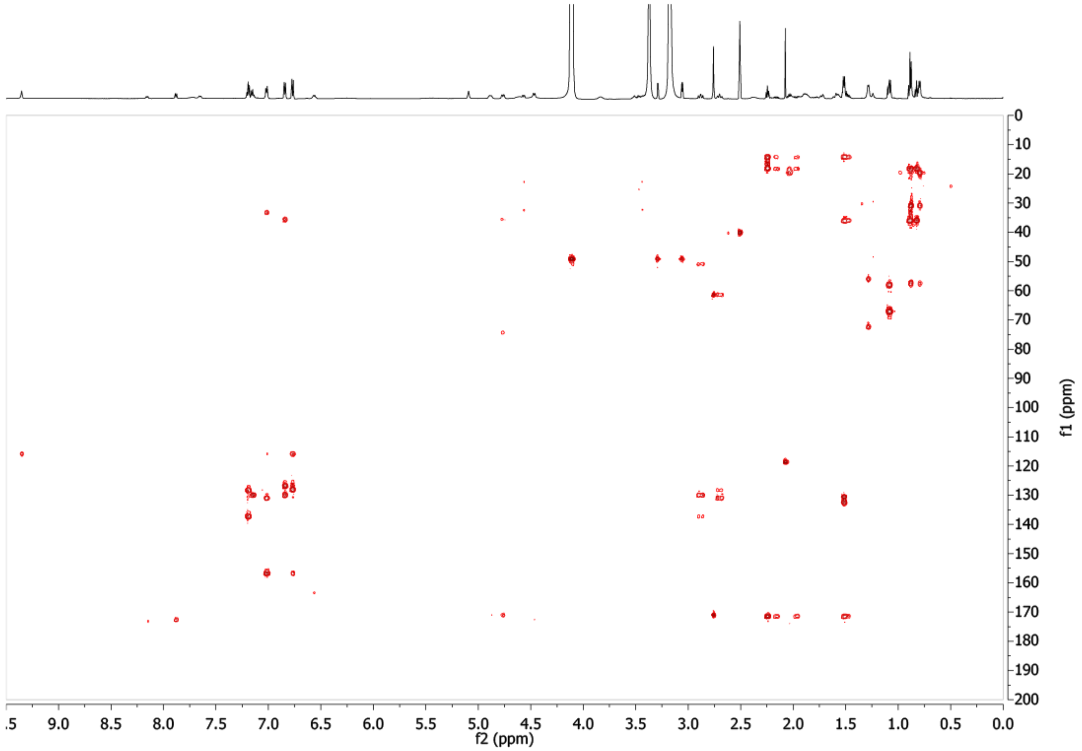

Supplementary Figure 29**:** ^{1}H-^{13}C HMBC spectrum of rivulariapeptolide 988 in DMSO-*d6*, 600 MHz.

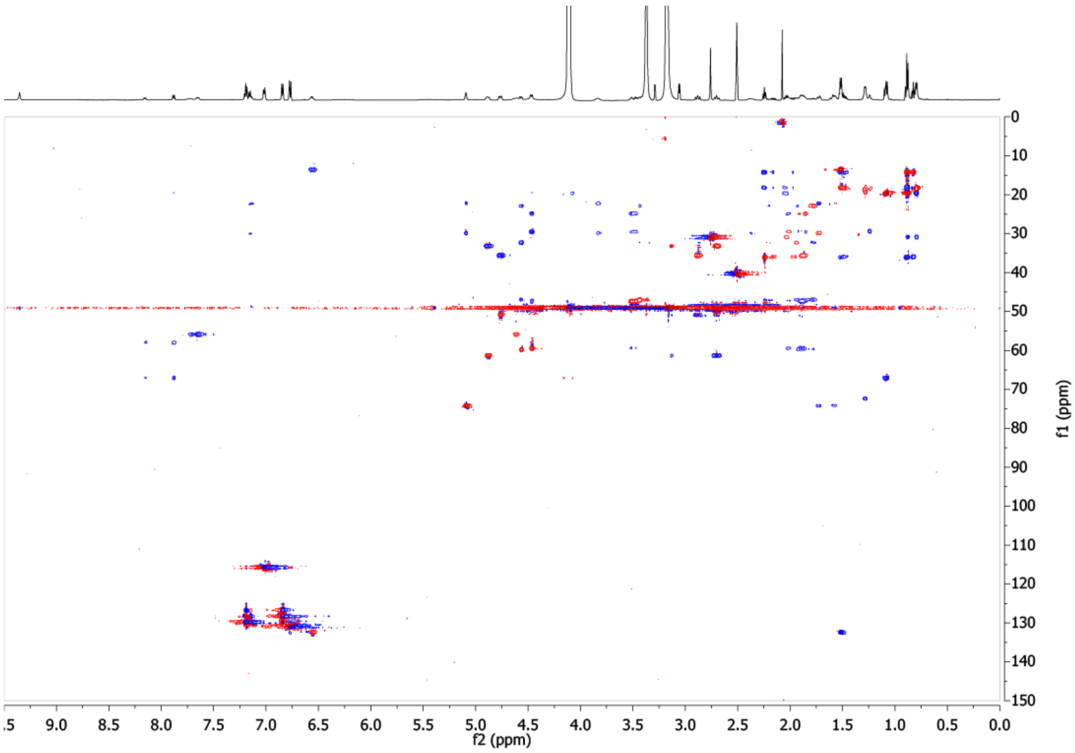

Supplementary Figure 30: ^{1}H-^{13}C HSQC-TOCSY spectrum of rivulariapeptolide 988 in DMSO-*d6*, 600 MHz.

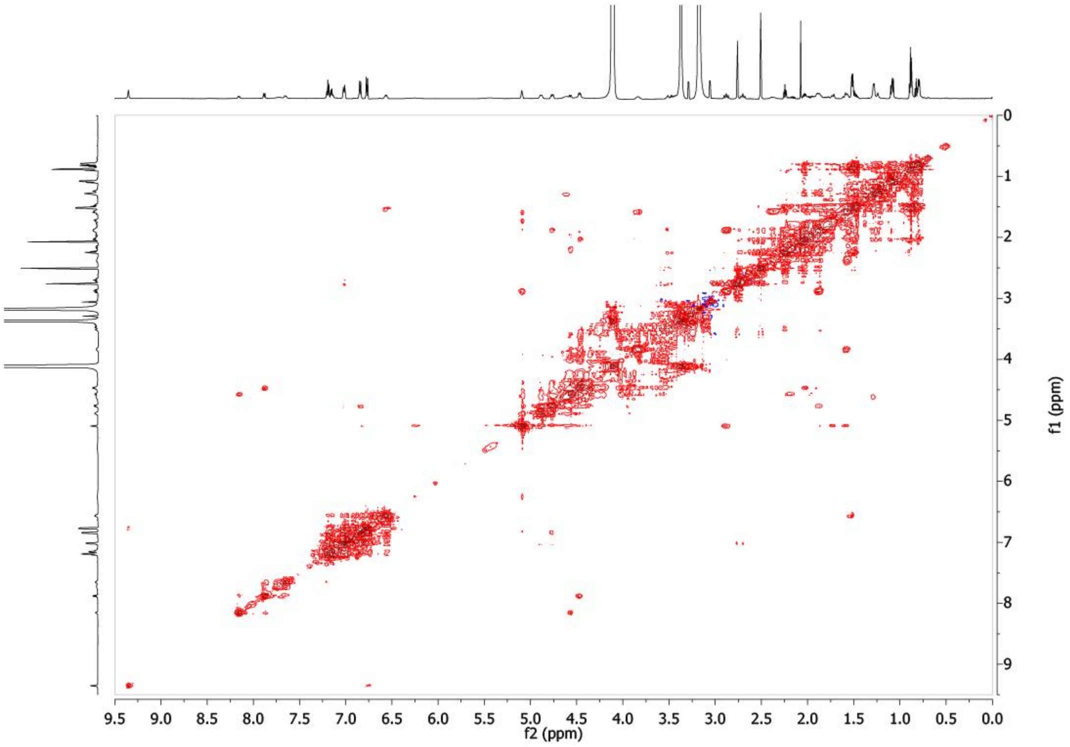

Supplementary Figure 31: ^{1}H-^{1}H ROESY spectrum of rivulariapeptolide 988 in DMSO-*d6*, 600 MHz.

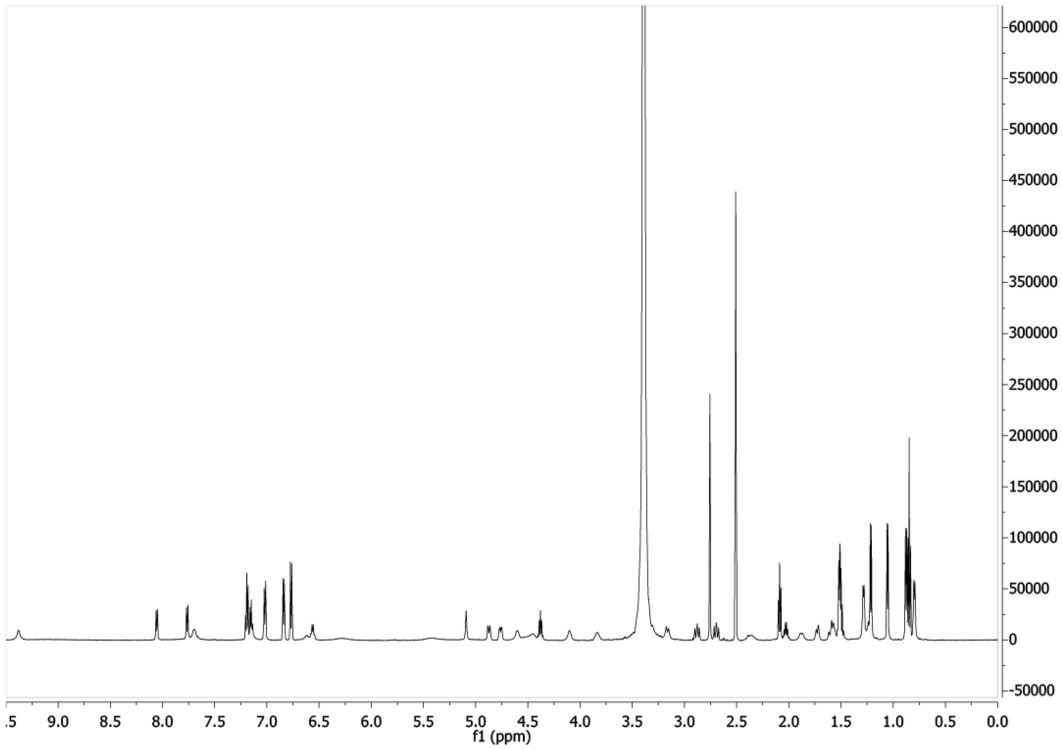

Supplementary Figure 32**:** ^{1}H NMR spectrum of molassamide (**5**) in DMSO-*d6*, 600 MHz.

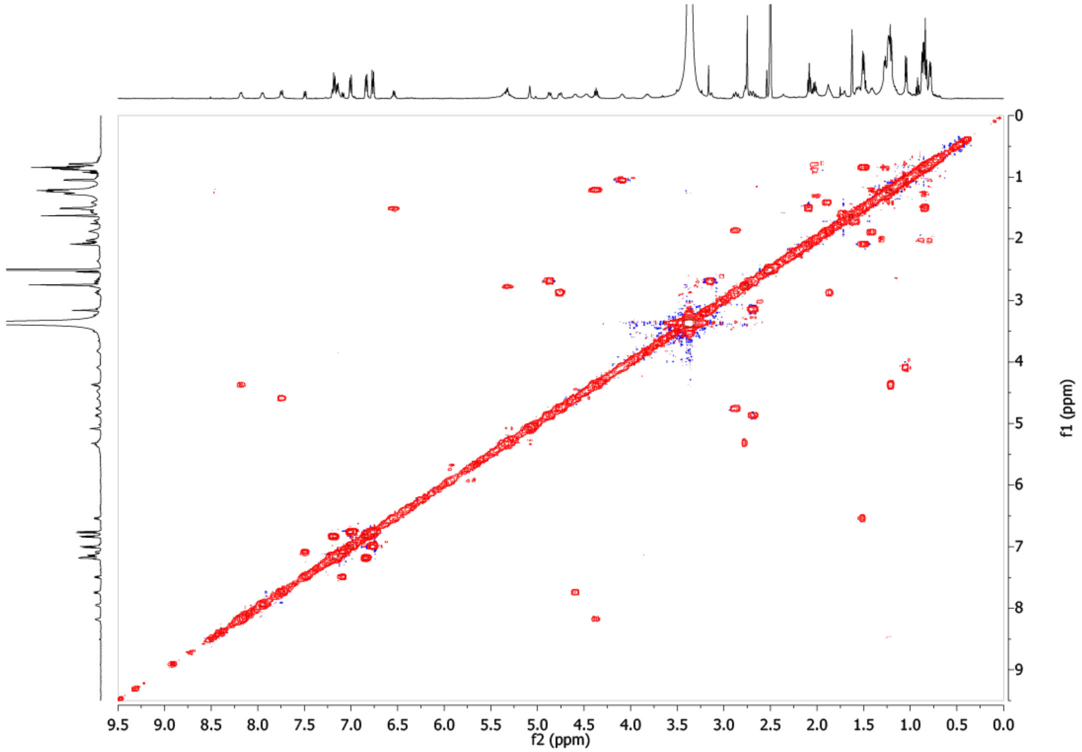

Supplementary Figure 33: ^{1}H-^{1}H COSY spectrum of molassamide in DMSO-*d6*, 600 MHz.

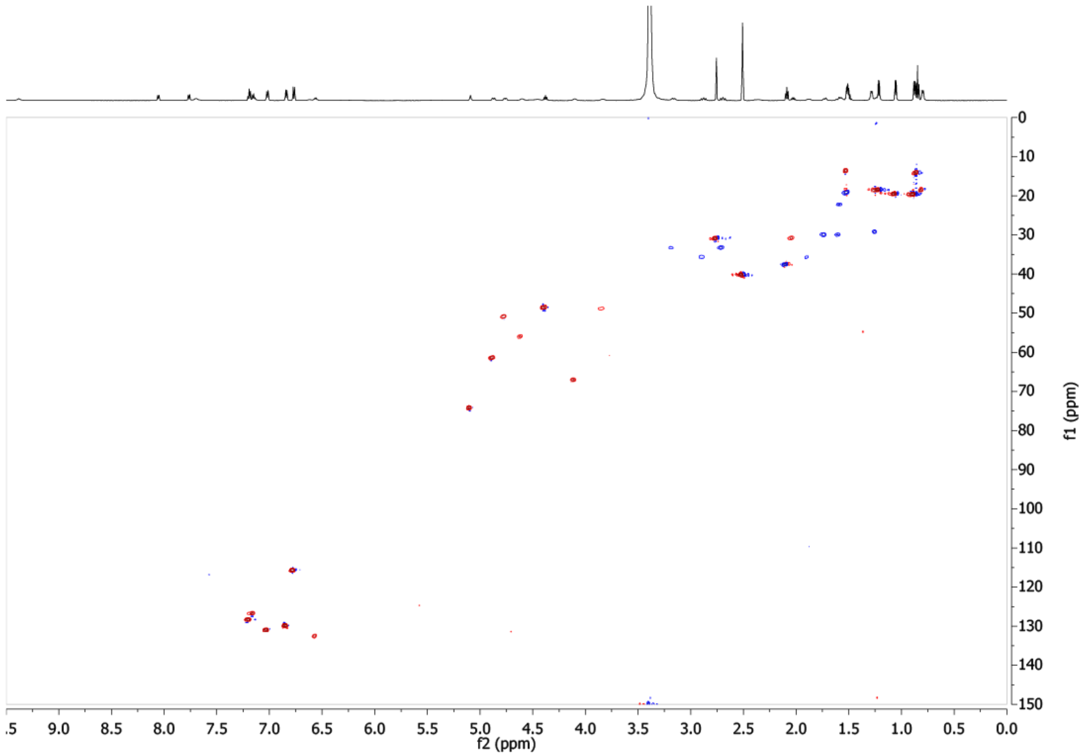

Supplementary Figure 34**:** ^{1}H-^{13}C HSQC spectrum of molassamide in DMSO-*d6*, 600 MHz.

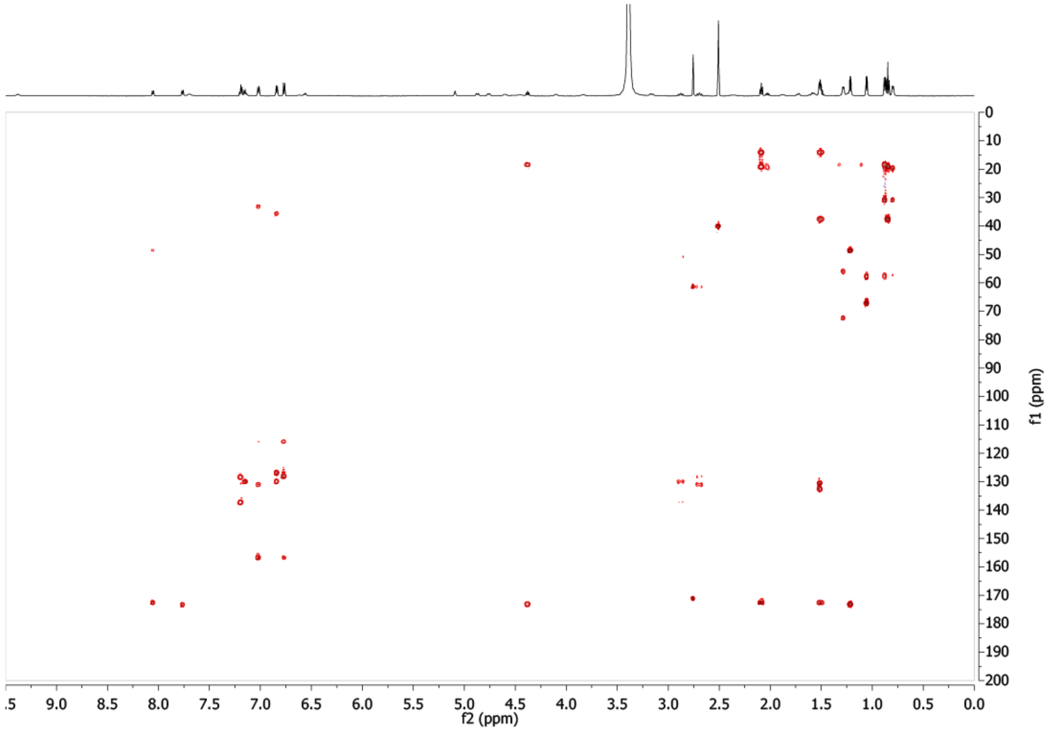

Supplementary Figure 35**:** ^{1}H-^{1}H HMBC spectrum of molassamide in DMSO-*d6*, 600 MHz.

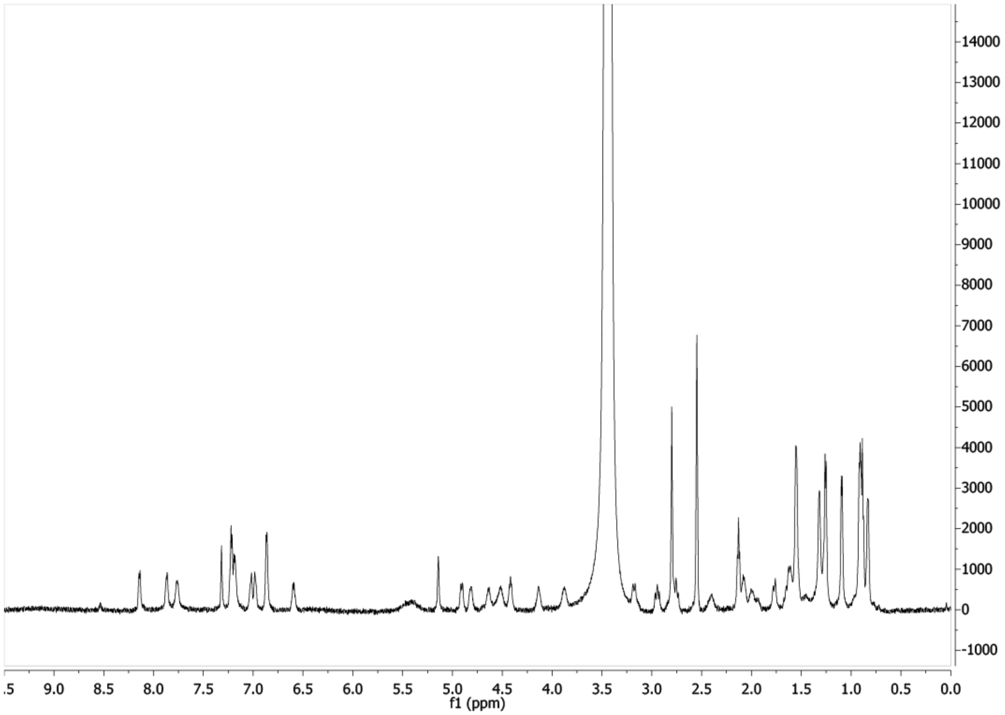

Supplementary Figure 36: ^{1}H NMR spectrum of molassamide B in DMSO-*d6*, 600 MHz.

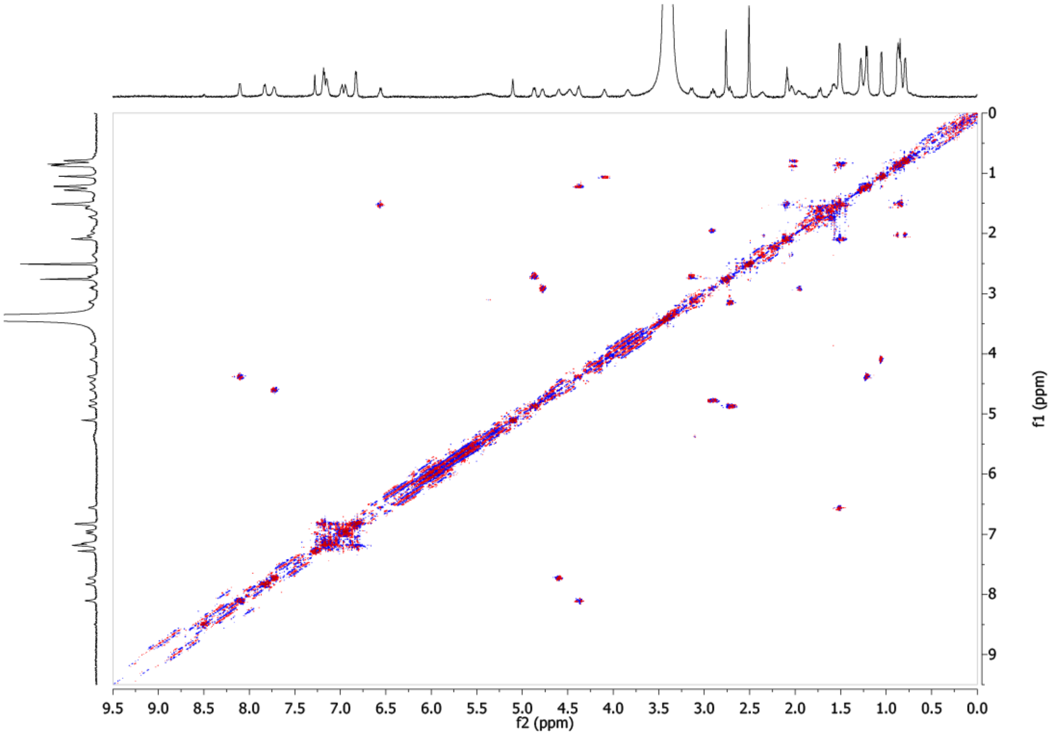

Supplementary Figure 37: ^{1}H-^{1}H COSY spectrum of molassamide B in DMSO-*d6*, 600 MHz.

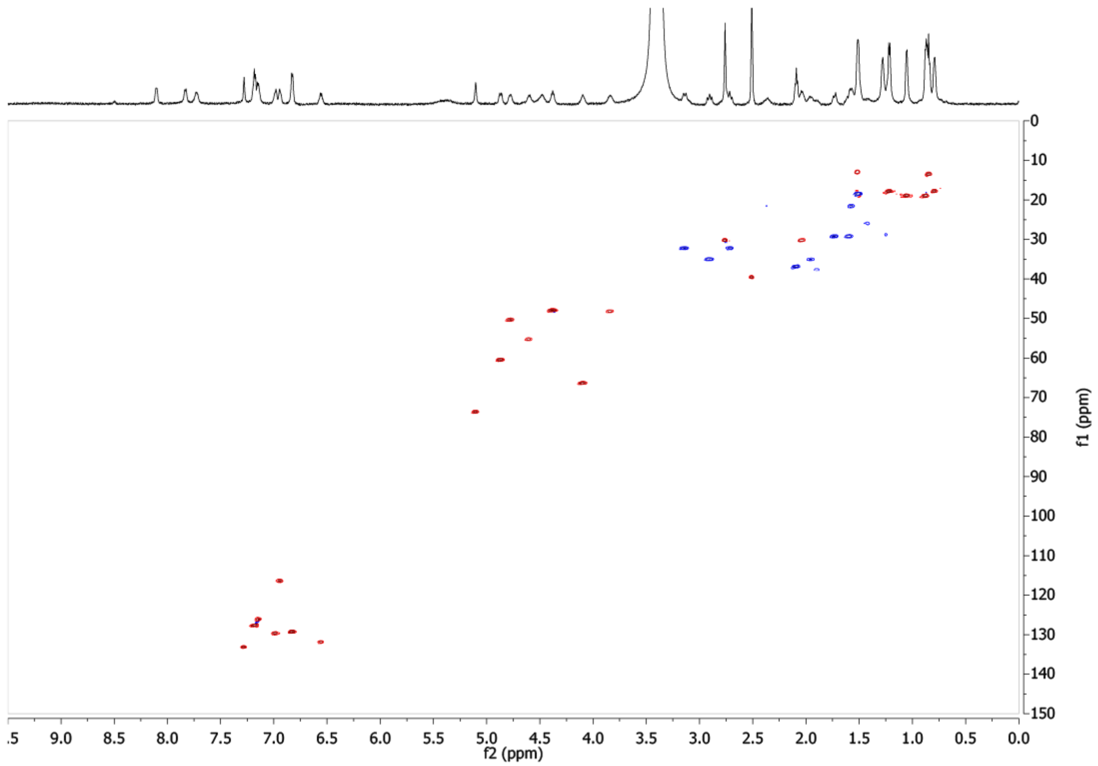

Supplementary Figure 38: ^{1}H-^{13}C HSQC spectrum of molassamide B in DMSO-*d6*, 600 MHz.

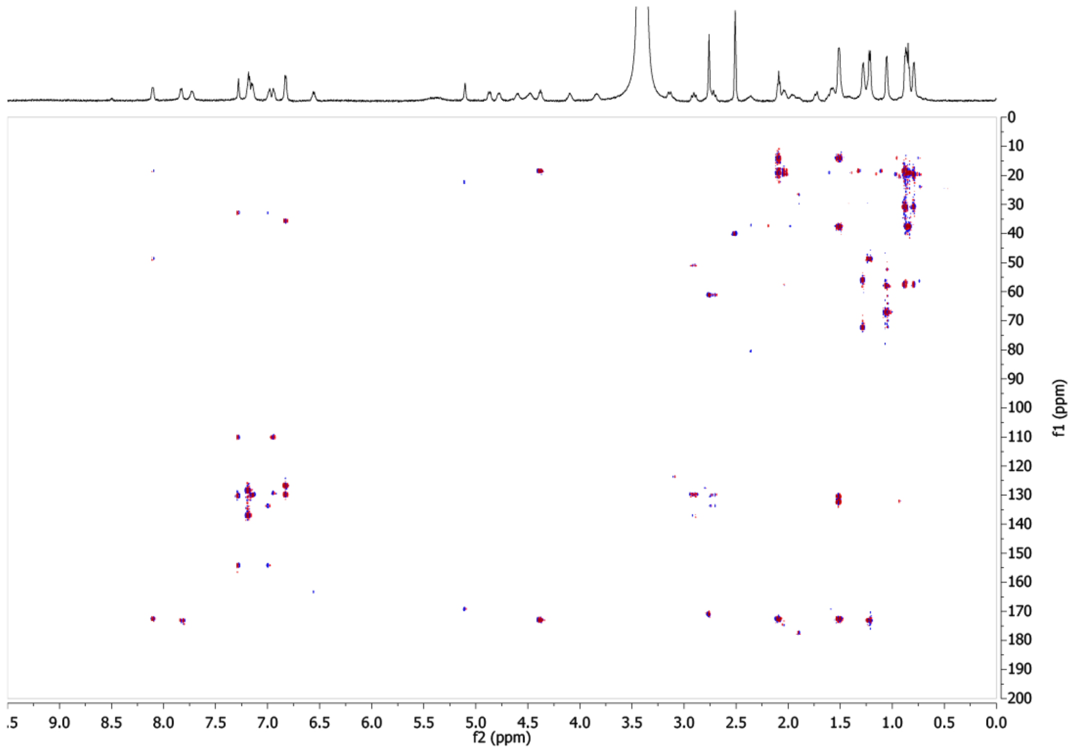

Supplementary Figure 39: ^{1}H-^{13}C HMBC spectrum of molassamide B in DMSO-*d6*, 600 MHz.

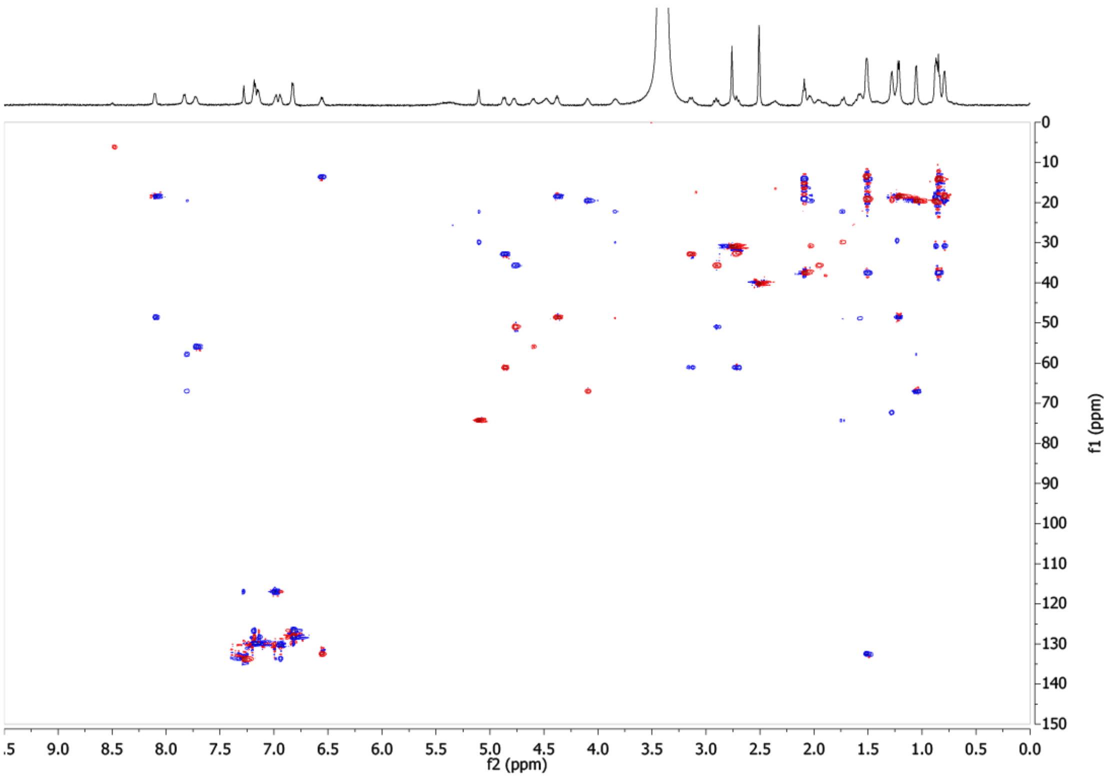

Supplementary Figure 40: ^{1}H-^{13}C HSQC-TOCSY spectrum of molassamide B in DMSO-*d6*, 600 MHz.

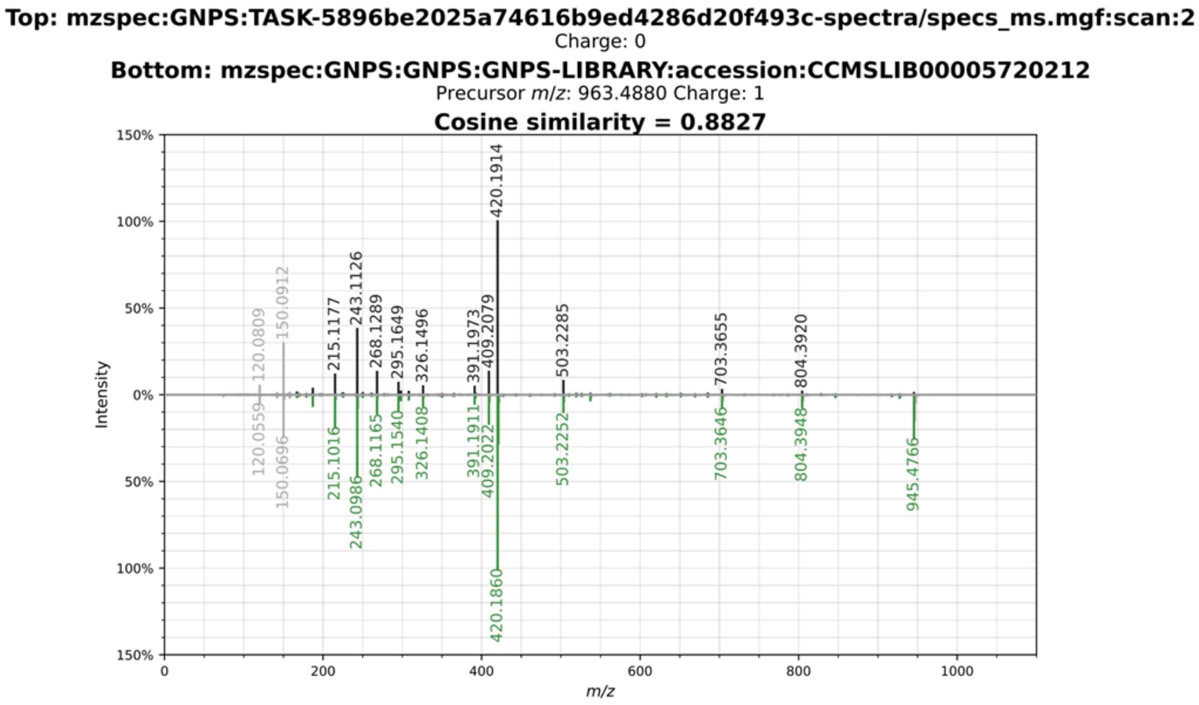

Supplementary Figure 41: Mirror MS^2 plot of molassamide from this study (black) and GNPS library spectrum (green).

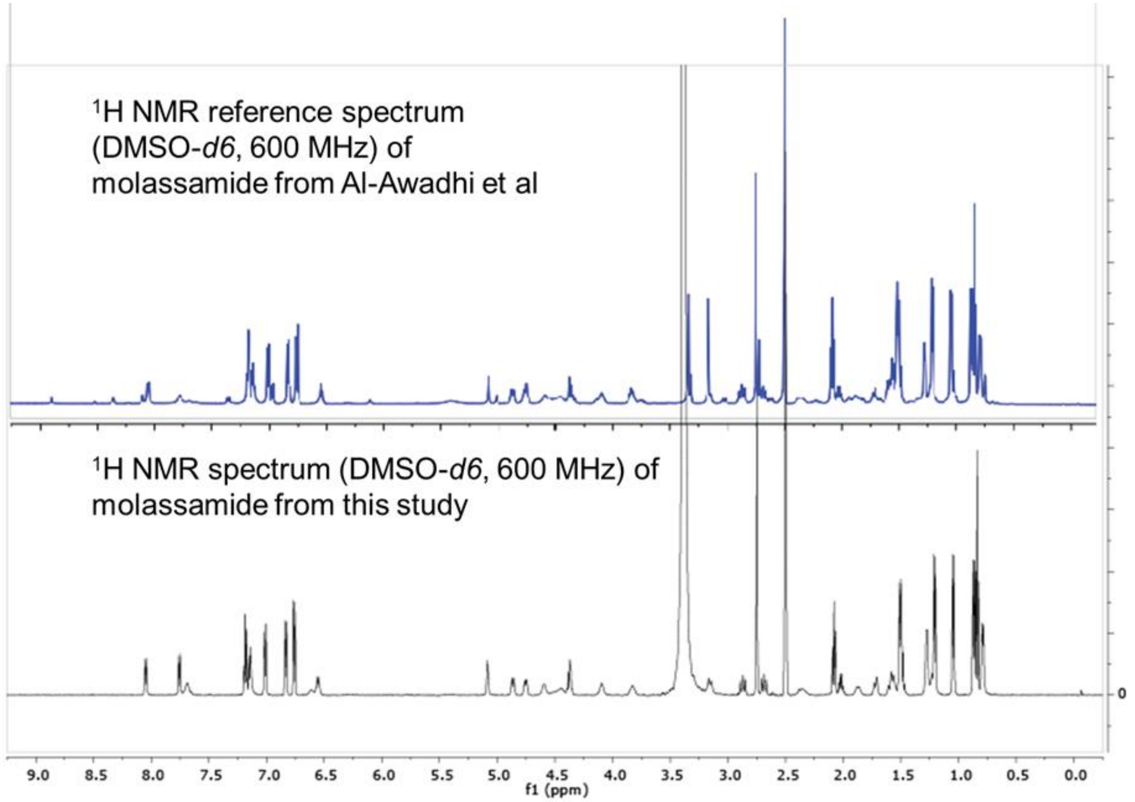

Supplementary Figure 42: Comparison of 1H spectra of molassamide from this study and molassamide isolated by Al-Awadhi et al.[10]

Rivulariapeptolide 1155

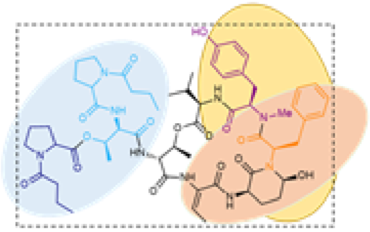

AA code	Immonium ion (calcd)	Immonium ion (obsvd)
Phe	120.0808	120.081
N-Me-Tyr	150.0913	150.0914
N-Bu-Pro	168.1019	168.1019
N-Bu-Pro-CO	140.1070	140.1070
Leu	86.0964	**n.d.**
Ahp-Phe+CO-OH	215.1179	215.1179
Thr-N-Bu-Pro	241.1547	241.1546
Ahp-Phe-OH	243.1128	243.1126
Ahp-OH-Phe-N-Me-Tyr	420.1918	420.1917
(N-Bu-Pro)$_2$-Thr	436.2442	436.2441
Abu-Ahp-OH-Phe	326.1499	326.15
N-Me-Tyr-Phe-Ahp-Abu	503.2289	503.23
Abu-Thr-OH-Thr-(N-Bu-Pro)2	602.3184	602.3167
Thr-Val-N-Me-Tyr-Phe-Ahp-Abu	703.345	703.3461
Abu-Thr-OH-Thr-((N-Bu-Pro)2)-Val	719.3974	719.3947

Rivulariapeptolide 1185

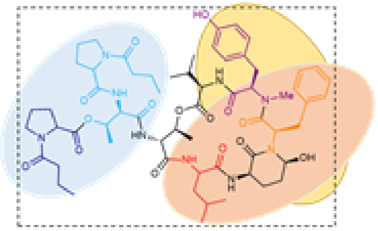

AA code	Immonium ion (calcd)	Immonium ion (obsvd)
Phe	120.0808	120.0811
N-Me-Tyr	150.0913	150.0914
N-Bu-Pro	168.1019	168.1019
N-Bu-Pro-CO	140.1070	140.1070
**Leu**	86.0964	**86.0971**
Ahp-Phe+CO-OH	215.1179	215.1174
Thr-N-Bu-Pro	241.1547	241.1547
Ahp-Phe-OH	243.1128	243.1128
Ahp-OH-Phe-N-Me-Tyr	420.1918	420.1924
(N-Bu-Pro)2-Thr	436.2442	436.244
**Leu**-Ahp-OH-Phe	356.1969	**356.1965**
**Leu**-Thr-OH-Thr-(N-Bu-Pro)2	632.3654	**632.3650**
Thr-Val-N-Me-Tyr-Phe-Ahp-**Leu**	733.3919	**733.3907**

Rivulariapeptolide 1121

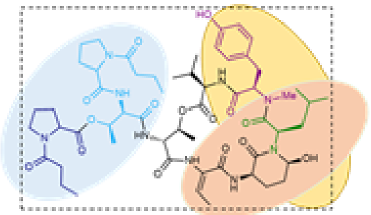

AA code	Immonium ion (calcd)	Immonium ion obsvd)
Phe	120.0808	**n.d.**
N-Me-Tyr	150.0913	150.0914
N-Bu-Pro	168.1019	168.1018
N-Bu-Pro-CO	140.1070	140.1070
**Leu**	86.0964	**86.0969**
Ahp+CO-OH-**Leu**	181.1335	**181.1335**
Thr-N-Bu-Pro	241.1547	241.1545
Ahp-OH-**Leu**	209.1285	**209.1284**
AHP-OH-**Leu**-N-Me-Tyr	386.2074	**386.2072**
(N-Bu-Pro)$_2$-Thr	436.2442	436.244
Abu-Ahp-OH-**Leu**	292.1656	**292.1647**
N-Me-Tyr-**Leu**-Ahp-Abu	469.2445	**469.2449**
Abu-Thr-OH-Thr-(N-Bu-Pro)2	602.3184	602.3181
Thr-Val-N-Me-Tyr-**Leu**-Ahp-Abu	669.3606	669.3585
Abu-Thr-OH-Thr-((N-Bu-Pro)2)-Val	719.3974	719.4006

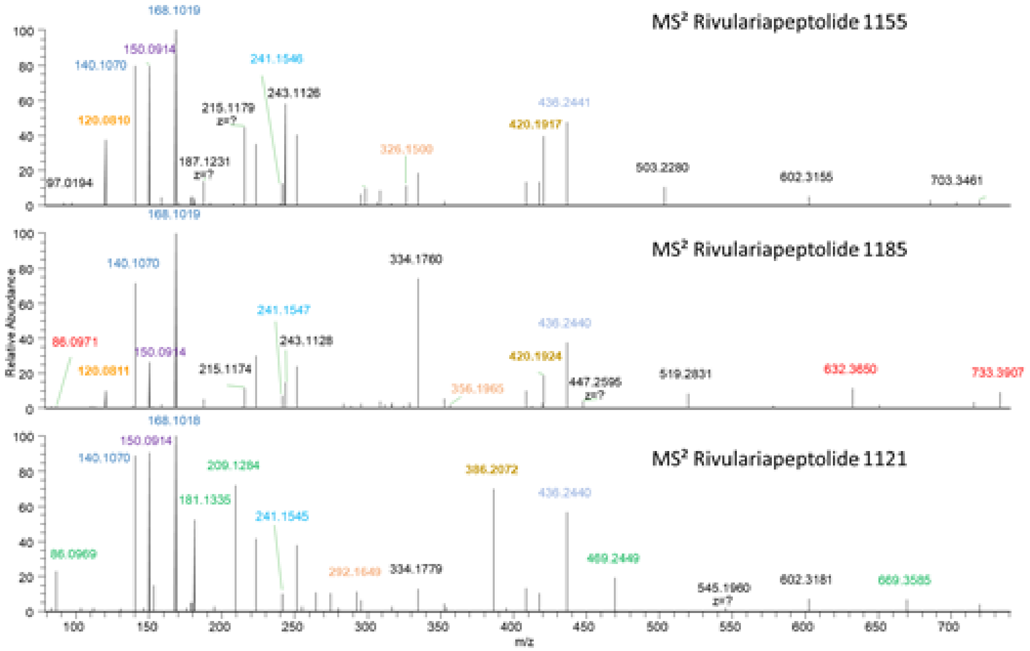

Supplementary Figure 43: MS/MS annotations for rivulariapeptolides 1155, 1185, and 1121. Ions that helped to elucidate the amino acid identity and position for the new rivulariapeptolides 1185, and 1121 are highlighted in red and green, respectively. Rivulariapeptolide 1185 contains Leu instead of Abu in position 5 and rivulariapeptolide 1121 contains Leu instead of Phe in position 3, compared with rivulariapeptolide 1155 n.d. = not detected.

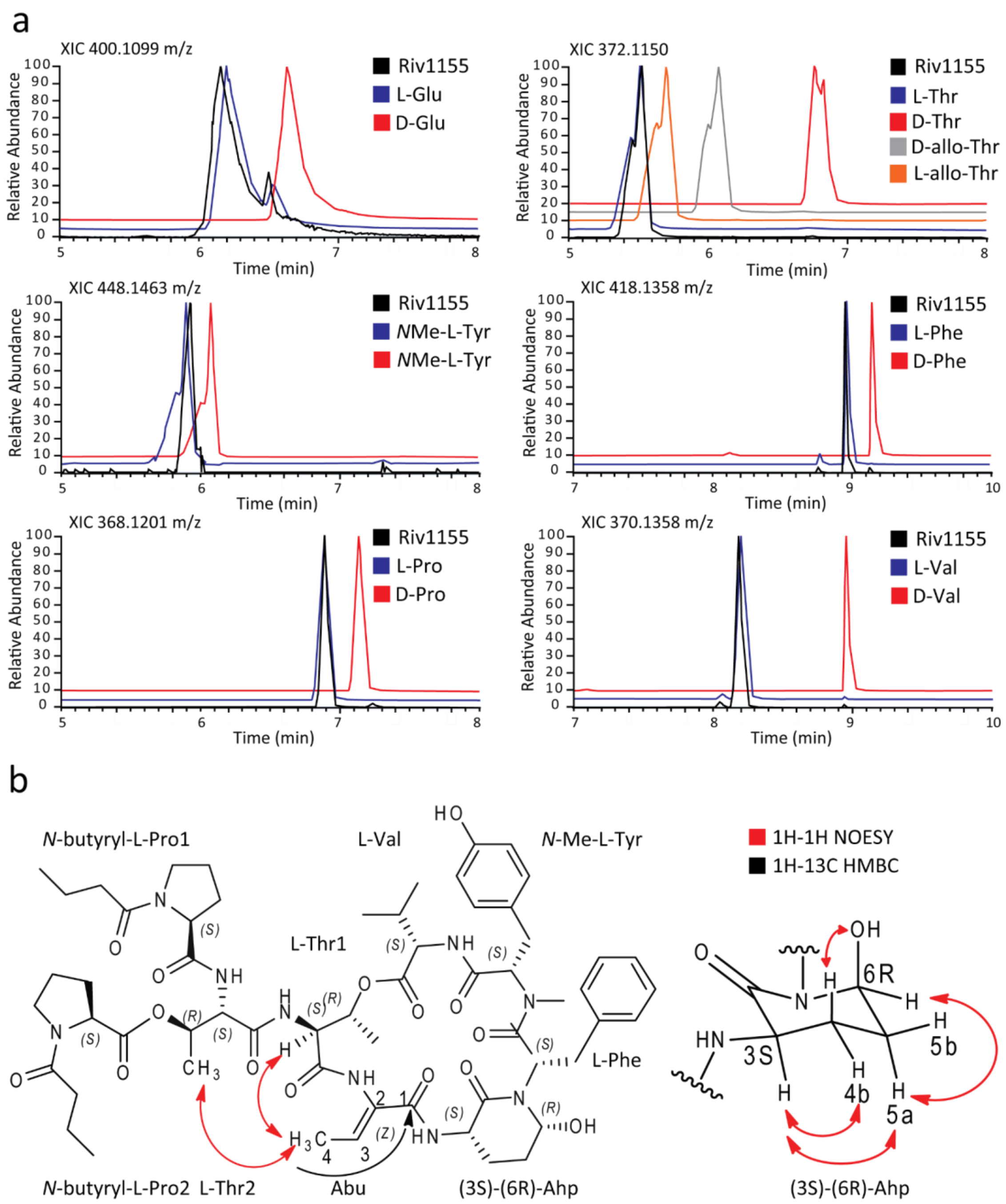

Supplementary Figure 44: Determination of relative and absolute stereochemistry of rivulariapeptolides. (a) XICs of (PDC oxidation), total hydrolysis, and Marfey's derivatization analysis of rivulariapeptolide 1155 compared with in the same way derivatized amino acid standards. (b) Structure of rivulariapeptolide 1155 with assigned stereo configurations, including selected 2D 1H-1H-NOESY and 1H-13C-HMBC NMR correlations used to determine the relative configuration of C-3 and C-6 from the Ahp unit and the geometry of the double bond from the Abu moiety. Abbreviations for amino acids: L-Val = L-valine, *N*-Me-L-Tyr = *N*-methyl-L-tyrosine, L-Phe = L-phenylalanine, L-Ahp = (3S)-amino-(6R)-hydroxy-2-piperidone, Abu = 2-aminobut-2-enoic acid, L-Thr1 = L-threonine1, L-Thr2 = threonine2, *N*-Bu-L-Pro1 = *N*-butyryl-L-proline1, *N*-Bu-L-Pro2 = *N*-butyryl-L-proline2.

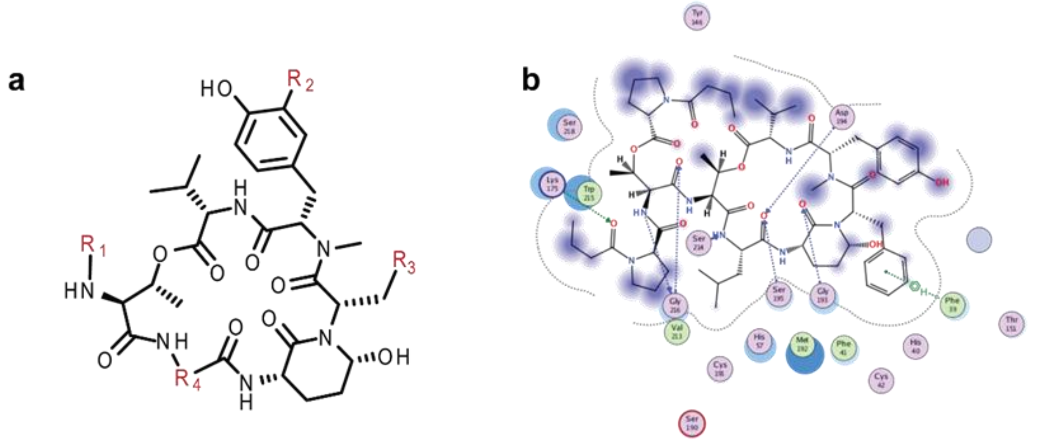

c

Compound	R1	R2	R3	R4
Rivulariapeptolide 1155	(*N*-Ba-Pro)$_2$-Thr-	H	Ph	-C=CH-CH$_3$
Rivulariapeptolide 1185	(*N*-Ba-Pro)$_2$-Thr-	H	Ph	-CH-CH$_2$-iPr
Rivulariapeptolide 1121	(*N*-Ba-Pro)$_2$-Thr-	H	iPr	-C=CH-CH$_3$
Rivulariapeptolide 988	*N*-Ba-Pro-Thr-	H	Ph	-C=CH-CH$_3$
Molassamide	*N*-Ba-Ala-Thr-	H	Ph	-C=CH-CH$_3$
Molassamide B	*N*-Ba-Ala-Thr-	Br	Ph	-C=CH-CH$_3$

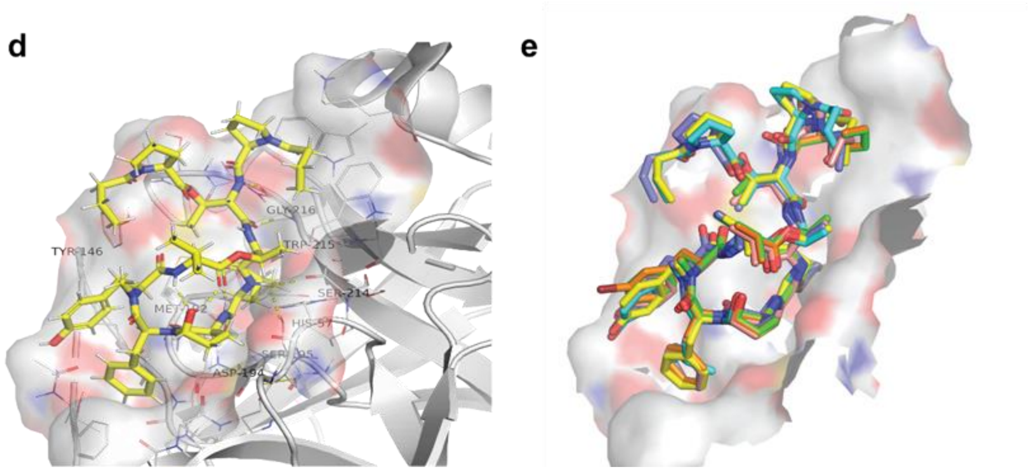

Supplementary Figure 45: Docking studies of rivulariapeptolides. (a) Structures of the isolated rivulariapeptolides 1155 (**2**), 1185 (**1**), 1121 (**3**), 988 (**4**), molassamide (**5**), and molassamide B (**6**). (b) Proposed binding sites of **1** in the active site of alpha-chymotrypsin (pdb id 4Q2K) using MOE software. (c) Potency of isolated compounds for selected serine proteases following 40 min pre-incubations. Data are presented as the mean ± SD, n = 3. (d) Induced-fit docking of rivulariapeptolide 1185 inside the binding pocket of alpha-chymotrypsin (pdb id 4Q2K using MOE). The ligand is shown in sticks, the binding pocket

as a surface, and the residues of the pocket are shown as lines and labeled. (d, e) All the isolated compounds compared to one another docked onto the surface of alpha-chymotrypsin (pdb id 4Q2K).

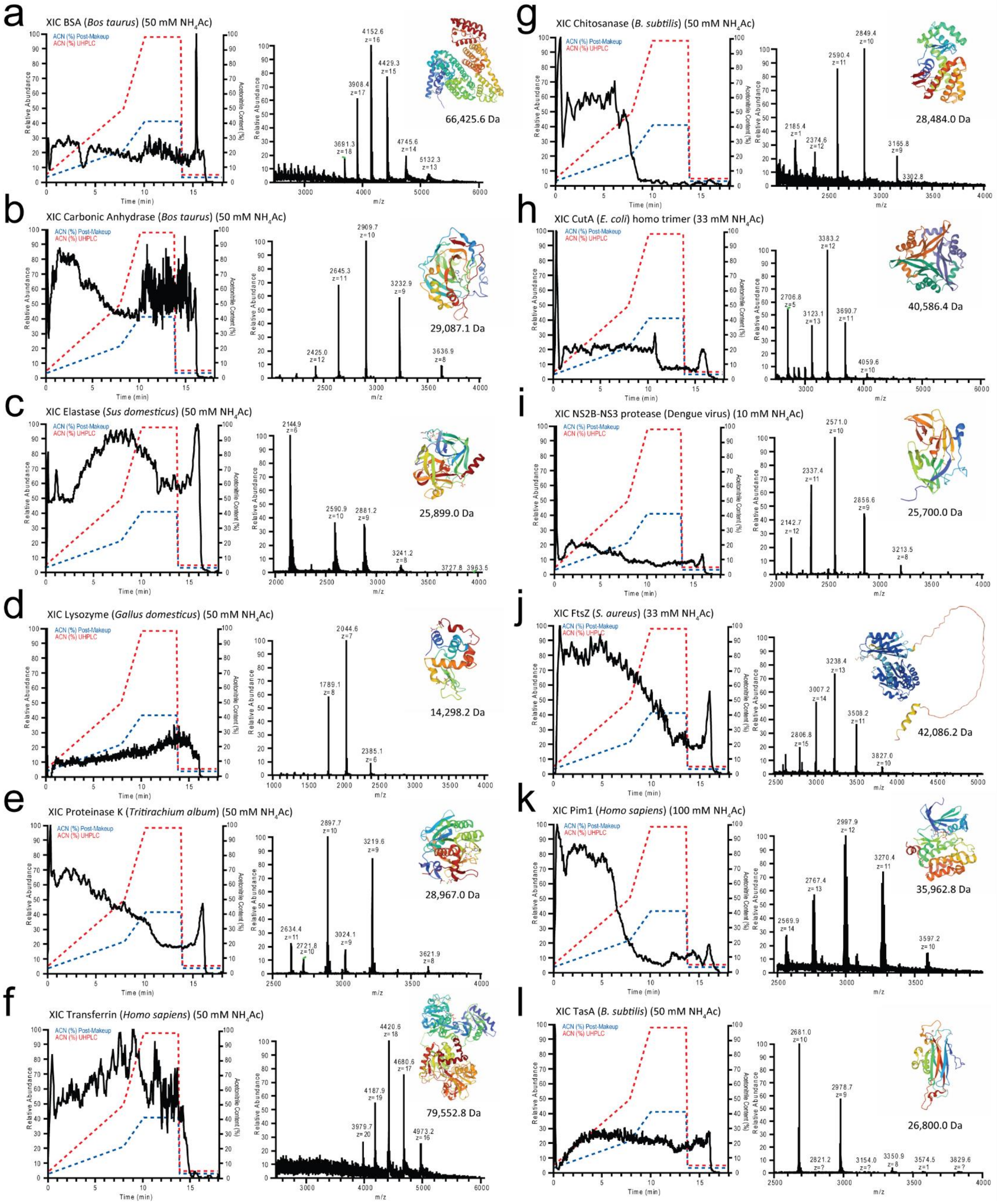

Supplementary Figure 46: Assessment of accessibility of different protein targets for native metabolomics. Extracted ion chromatograms and Full mass spectrum of target proteins under native MS conditions. Acetonitrile (ACN) concentration on column and post-column (including make-up) are shown as dashed

lines. The ACN concentrations are given at pump for a given time and a ~ 2 min delay time between pump and column has to be taken into account. Mobile phase NH_4Ac concentrations are indicated in the headings for each protein. (a) BSA (bovine serum albumin, *Bos taurus,* pdb id 4F5S). (b) Carbonic anhydrase (*Bos taurus,* pdb ID 1V9E). (c) Elastase (*Sus domesticus,* pdb id 1INC). (d) Lysozyme (*Gallus domesticus,* pdb id 5YIN). (e) Proteinase K (*Tritirachium album*, pdb id 2PRK). (f) Transferrin *Homo sapiens* pdb id 6CTC). (g) Chitosanase (*Bacillus subtilis,* pdb id 7C6C). (h) CutA (Divalent-cation tolerance protein CutA. *Escherichia coli*) pdb id 3X3U, (i) NS2B-NS3 protease (Dengue NS2B-NS3 protease, *Dengue virus,* pdb id 4M9I). (j) FtsZ (Cell division protein FtsZ, *Staphylococcus aureus*, pdb id).(k) Pim1 (Serine/threonine-protein kinase pim-1, *Homo sapiens,* pdb id 1YXT). (l) TasA (TasA protein, *Bacillus subtilis,* pdb id 5OF1).

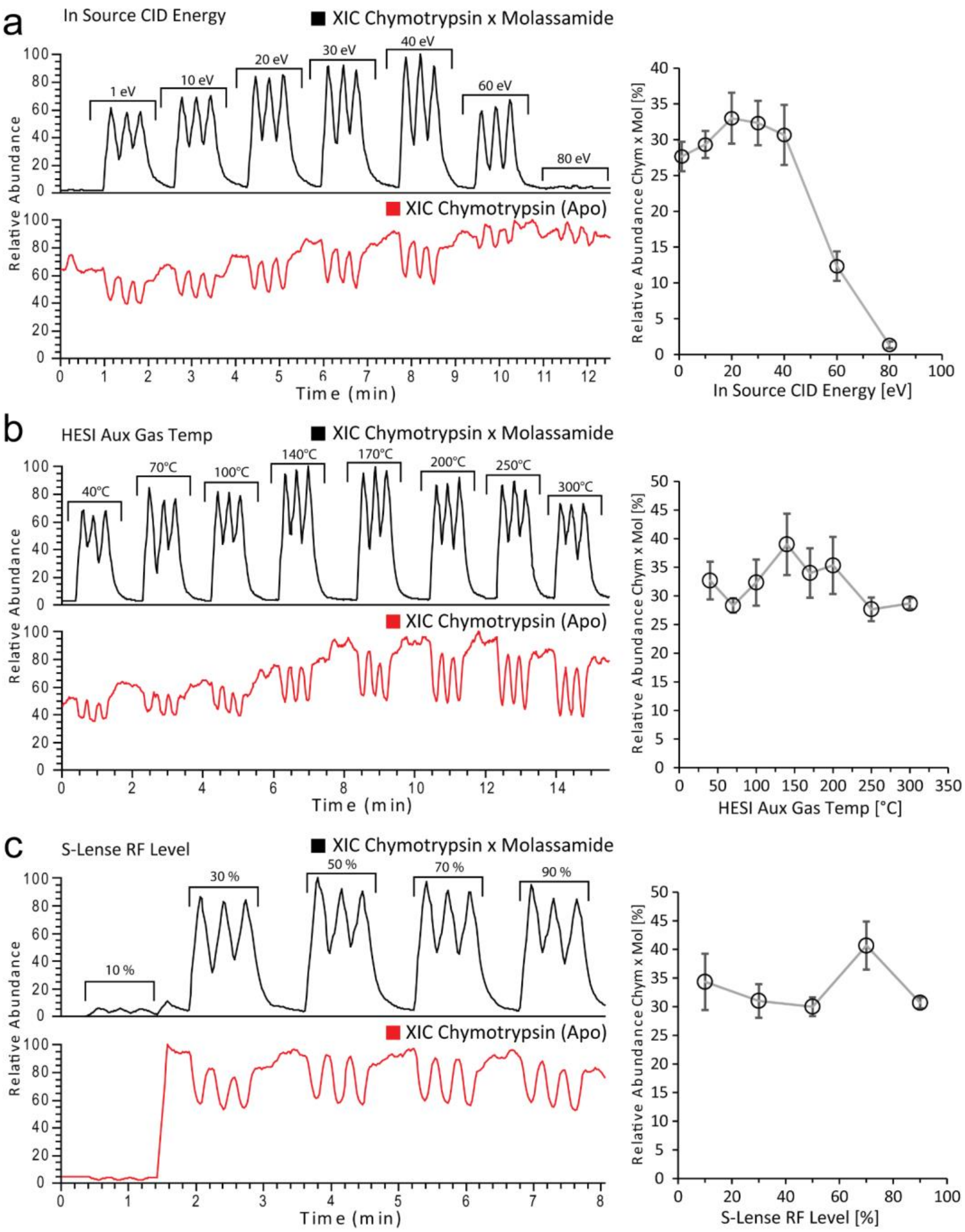

Supplementary Figure 47**:** Assessment of native electrospray and in-source dissociation conditions for chymotrypsin and molassamide. Flow injections of molassamide were performed in technical triplicates (Error bars are Data are presented as mean values +/- SD as appropriate, n = 3) for each condition. a) shows the XICs of the chymotrypsin x molassamide complex (top left) and apo chymotrypsin (bottom left) over stepwise ramped in source CID energy (1-80 eV). The scatter-plot on the right shows the relative peak height (apo vs. holo) over the different CID energy. b) displays the XICs of the chymotrypsin x molassamide complex (top left) and apo chymotrypsin (bottom left) over stepwise increased HESI AUX temperatures (40-300 °C). The scatter-plot on the right shows the relative peak height (apo vs. holo) over the different temperature steps. c) displays the XICs of the chymotrypsin x molassamide complex (top left) and apo chymotrypsin (bottom left) over stepwise increased relative S-lense radiofrequency (RF) level (10-90%). The scatter-plot on the right shows the relative peak height (apo vs. holo) over the different RF levels. Source data are provided as a Source Data file.

Supplementary Table 1: NMR table for rivulariapeptolide 1185 (**1**) at 600 MHz (^{1}H), 150 MHz (^{13}C) in DMSO-*d6* and in MeOH-d4 at 500 MHz (^{1}H), 125 MHz (^{13}C), respectively.

Unit	No.	δ_H (*J* in Hz)[a]	δ_C[a]	TOCSY	HMBC	NOESY
**Val**	1		172.0		3 (Thr-1)	
	2	4.53, m	58.8	NH	4,5	3,4,5, NH
	3	2.03, m	30.6	4,5	2,3,5	2,4,5, NH
	4	0.85, d (6.7)	19.2	3	2,3,4	2, 3 NMe (Tyr)
	5	0.69, d (6.9)	16.9	3		2, 3 NMe (Tyr), NH
	NH	8.03, d (7.9)			1	2,3,4,5, NMe (Tyr), 2 (Tyr)
***N*-Me-Tyr**	1		169.4			
	2	4.91, d (12.7)	60.6	3b, 3a	1,3b,N-Me	1 (Phe), 3a,b (Phe), H(Val)
	3a	3.08, dd (14.0, 2.1)	32.6	2, 3b	2, 4, 5/9	2, 3b, 5/9
	3b	2.71, dd (13.8, 11.8)	32.6	2, 3a	2, 4, 5/9	2,3a,5/9
	4		127.5			
	5/9	7.00, d (8.1)	130.0	6/8	7, 3	6/8, N-Me (weak), 3b
	6/8	6.78, d (8.4)	115.0	5/9	4, 7 (weak)	5/9, 7-OH
	7		156.3			
	7-OH	9.36, s		6,7,8	6,7,8 (Tyr)	6 (AHP), 6/8 (Tyr)
	N-Me	2.76	30.1		2, 1(Phe)	5/9, 4,5,NH (Val)
**Phe**	1		170.5			
	2	4.74, dd (11.9, 4.0	50.0	3a, (3b)	3a,b, 1,6 (Ahp)	3b, 3a, 5/9 2, 5/9 (NMe-Tyr)
	3a	2.86, dd (-13.6, 12.1)	35.3	2, 3b	2, 4, 5/9	2,3b, 5/9, 6 (AHP)
	3b	1.78, m	35.3	2, 3a	2, 4, 5/9	2,3b, 5/9, 6 (AHP)
	4		136.8			
	5/9	6.83, d (7.2)	129.1	6/8, 7	3,5/9,7	2,3a,3b,6/8 (Phe) 5a,5b,6(Ahp)
	6/8	7.18, t (7.2)	127.4	5/9, 7	4, 6/8	5/9 (Phe)3,4, 5a/b (Ahp)
	7	7.14, t (7.2	125.8	5/9, 6/8	5/9	5/9
**Ahp**	2		169.0			
	3	3.63, m	48.2	4a,4b, 5a,b NH	2, 4a,4b	4a,4b,5a, NH
	4a	2.41, m	21.1	3, 4b, 5b		3, 4b,5a,5b NH
	4b	1.56, m	21.1	3, 4a, 5a		3, 4a5a,5b NH
	5a	1.68, m	29.2	5b, 6		4a,4b, 5b6, NH
	5b	1.56, m	29.2	5a,6		5a,6, NH, 5/9 (Phe)
	6	5.05, br s	73.3	5a,5b,6-OH	2	5a,5b,6-OH (AhP) 3a,b,5/9 (Phe)
	6-OH	6.01, br s		6		
	NH	7.06		3	4,5	3, 4a/4b, 2 (Leu)

**Leu**	1		171.8			
	2	4.23, m	50.0	3a, 3b, 4		3a, 3b, 5, NH (AHP), NH (Thr-1)
	3a	1.30	38.8	2, 4, NH	1,4	4, NH
	3b	1.68	38.8	2, 4, NH		
	4	1.47	23.6			
	5	0.84	23.2	2, 3a, 3b, 5	1,2,3	3, NH
	6	0.72, d (6.5)	20.4			
	NH	8.35		2, 3a, 3b		2,3 (Thr-1), NH (Ahp)
**Thr-1**	1		169.5		NH	
	2	4.62, m	55.4	NH		3,4, NH (Thr-1), 3,4, NH(Thr-2), NH (Abu)
	3	5.40, m	71.4	4		2,4, NH (Ahp), NH (Abu)
	4	1.19, d (6.6)	17.3	3	2,3	2,3, NH (Abu), NH (Thr-2)
	NH	7.38		2	1	2 (Pro-2), 3 (Thr-2), 4 (Pro-1), 4 (Pro-2)
**Thr-2**	1		169.1			
	2	4.58, m	54.7	3,4,NH		3,4, NH
	3	4.99, m	69.3	2,4, NH		2,4, 2 (Val), NH, NH (Thr-1)
	4	1.06, d (6.5)	15.2	2,3	2,3	3, NH, NH(Thr-1)
	NH	7.87		2,3		2, 3, 4 (Thr-2), 2 (Pro-1), 3b (Pro-2)
**Pro-1**	1		171.7			
	2	4.45, m	58.5	3a,3b, 4, 5a, 5b		
	3a	1.97, m	28.6	2,4		
	3b	1.89, m	28.6	2,4		
	4	1.88, m	24.1	5a		
	5a	3.52, m	46.5	3,4	1	2,34
	5b	3.46, m	46.5	4	1,2,4	
**Ba-1**	1		171.6			
	2	2.26, t (7.2)	35.6		1,3,4	5a (Pro-1)
	3	1.52, m	17.5		1,2,4	
	4	0.91, m	13.8		2,3	
**Pro-2**	1		170.6			
	2	4.28, m	58.5	3a,3b, 4, 5af, 5b	1(Ba-2)	
	3a	2.20, m	28.2	2,4		
	3b	2.18, m	28.2	2,4		
	4	1.97, m	24.2	5a		
	5a	3.60, m	46.4	4	2,3,4	
	5b	2.43, m	46.4	4	2,3,4	
**Ba-2**	1		171.5			
	2	2.25, m	35.1		1,3,4	5a (Pro-2)
	3	1.49, m	17.5		1,2,4	
	4	0.84, m	13.7		2,3	

[a] Assignments are based on extensive 1D and 2D NMR measurements (^{1}H, HMBC, HSQC, COSY, HSQC-TOCSY, NOESY). See also Figures S6-11.

Supplementary Table 2*:* NMR table for rivulariapeptolides 1155 (**2**), 1121 (**3**), and 988 (**4**) at 500 MHz and 600 MHz (^{1}H), respectively, 125 MHz and 150 MHz for (^{13}C) in DMSO-*d6*.

Unit	No.	2 δ_H [a]	2 δ_C[a]	3 δ_H [b]	3 δ_C[b]	4 δ_H [b]	4 δ_H [b]
Val	1		172.4		172.4		172.4
	2	4.54, m	55.9	4.53	55.9	4.54	55.9
	3	2.00, m	30.8	2.03	30.4	2.03	30.1
	4	0.86, d	19.4	0.85	19.2	0.88	18.8
	5	0.73, d	17.3	0.73	17.2	0.79	17.6

	NH	7.43, br s		7.51		7.43	
N-Me-Tyr	1		169.7		169.7		170.4
	2	4.90, d (12.7)	60.9	4.89	60.7	4.88	60.7
	3a	3.09, dd (14.0, 2.1)	32.8	3.10	32.7	3.12	32.6
	3b	2.70, dd (13.8, 11.8)	32.8	2.66	32.7	2.70	32.6
	4		127.5		127.1		127.5
	5/9	6.99, d (8.1)	130.5	6.89	129.9	7.01	130.3
	6/8	6.76, d (8.4)	115.4	6.61	115.2	6.76	115.1
	7		156.3		155.9		156.3
	7-OH	9.36, s		9.23		9.34	
	N-Me	2.76	30.5	2.70	30.4	2.75	30.5
Phe / Leu	1		170.6		173.6		170.8
	2	4.73, m	50.3	4.47	48.7	4.76	50.1
	3a	2.88, dd (-13.6, 12.1)	35.4	1.55	38.5	2.87	34.9
	3b	1.81, m	35.4	0.44	38.5	1.87	34.9
	4		136.8	0.98	23.6		136.7
	5/9	6.83, d (7.1)	129.5	0.69	23.8	6.84	129.2
	6/8	7.19, t (7.2)	127.9	0.50	22.1	7.19	127.6
	7	7.15, t (7.2	126.3			7.15	126.1
				NH, 6.59			
Ahp	2		168.5		168.5		168.5
	3	3.78, m	47.3	4.57	47.6	3.83	48.3
	4a	2.42, m	22.0	1.72	22.1	2.38	21.5
	4b	1.58, m	22.0	1.78	22.1	1.58	22.3
	5a	1.71, m	29.4	1.73	29.7	1.71	29.8
	5b	1.58, m	29.4	1.71, m	29.7	1.58	29.8
	6	5.07, br s	73.8	4.89	73.3	5.08	73.5
	6-OH	6.07, br s		6.06, br s		6.24	
	NH	7.21		7.21		7.21	
Abu	1		163.6		163.6		163.7
	2		130.0		129.8		130.2
	3	6.53, q (7.2)	132.3	6.63	132.4	6.56	131.8
	4	1.50, d (7.3)	13.4	1.51	13.3	1.52	13.0
	NH	9.36		9.36		9.35	
Thr-1	1		169.5		169.5		169.5
	2	4.59, m	55.9	4.63	55.9	4.61	55.2
	3	5.53, br s	71.8	5.62	71.3	5.44	71.7
	4	1.26, d (6.5)	17.8	1.29	17.6	1.26	18.1
	NH	7.93		7.93		7.88	
Thr-2	1		169.4		169.4		173.1
	2	4.69, m	55.0	4.70	55.1	4.69	57.7
	3	5.04, br s	69.6	5.05, br s	69.2	5.04	66.4
	4	1.11, dd (8.2, 6.4)	15.6	1.11	15.3	1.09	19.0
	NH	8.11		8.11		8.16	
Pro-1	1		171.9		171.9		172.6
	2	4.46, m	59.0	4.46, m	58.8	4.46	58.7
	3a	1.97, m	28.8	2.01	28.9	2.00	28.8
	3b	1.89, m	28.8	1.90	28.9	1.90	28.8
	4	1.88, m	24.3	1.95	24.2	1.87	24.1
	5a	3.52, m	46.9	3.51	46.6	3.51	46.7
	5b	3.45, m	46.9	3.42	46.6	3.47	46.7
Ba-1	1		171.0		170.7		171.1
	2	2.26, t (7.4)	35.6	2.25	35.4	2.24	35.4
	3	1.52, m	17.8	1.52	17.6	1.51	17.4
	4	0.91, m	13.8	0.90	13.6	0.88	13.5
Pro-2	1		170.6		170.8		
	2	4.27, m	58.9	4.28, m	58.7		
	3a	2.20, m	28.6	2.21	28.4		

	3b	2.18, m	28.6	2.19	28.4		
	4	1.97, m	24.6	1.9	24.2		
	5a	3.62, m	46.7	3.60	46.7		
	5b	2.43, m	46.7	3.37	46.7		
Ba-2	1		171.4		171.4		
	2	2.25, m	35.5	2.25	35.4		
	3	1.49, m	17.7	1.49	17.5		
	4	0.84, m	13.8	0.85	13.6		

[a] For assignments of compound **2**, see Dührkop et al[5] **[**b] Assignments are based on extensive 1D and 2D NMR measurements (^{1}H, ^{13}C, HMBC, HSQC, COSY, TOCSY, HSQC-TOCSY). See also Figures S12-S22.

Supplementary Table 3: NMR table for Molassamide (**5**) and Molassamide B (**6**) at 500 MHz and 600 MHz (^{1}H), respectively, 125 MHz and 150 MHz (^{13}C) in DMSO-*d6.*

Unit	No.	Molassamide δ_H	Molassamide δ_C[a]	Molassamide B δ_H	Molassamide B δ_C[a]
Val	1		172.9		173.0
	2	4.48, br	57.1	4.48	57.3
	3	2.02, m	30.2	2.04	30.1
	4	0.79, d (6.6)	17.9	0.79	17.7
	5	0.87, d (6.9)	19.1	0.88	18.9
	NH	7.75, d (8.2)		n.d.	
N-Me-(Br)-Tyr	1		169.7		169.9
	2	4.87, d (11.3)	60.8	4.87	60.6
	3a	3.16, d (11.5)	32.6	3.14	32.2
	3b	2.69, dd ()	32.6	2.71	32.2
	4		127.5		133.6
	5	7.01, d (8.4)	130.4	6.99	129.6
	6	6.76, d (8.4)	115.2	6.94	116.3
	7		156.3		154.0
	7-OH	4.45, br		4.47	
	8	6.76, d (8.4)	115.2	-	110.0
	9	7.01, d (8.4)	130.4	7.28	133.1
	N-Me	2.75	30.2	2.76	30.2
Phe	1		170.6		170.5
	2	4.76, dd (11.2, 4.0)	50.3	4.78	50.3
	3a	2.87, dd (14.0, 11.8)	35.0	2.90	34.9
	3b	1.87, d (11.5)	35.0	1.95	34.9
	4		136.8		136.7
	5/9	6.83, d (7.3)	129.3	6.83	129.2
	6/8	7.18, t (7.4)	127.7	7.18	127.7
	7	7.15, t (7.1)	126.1	7.15	126.1
Ahp	2		168.5		168.7
	3	3.83, br	48.2	3.84	48.1
	4a	2.37, m	21.6	2.37	21.6
	4b	1.56, m	21.6	1.57	21.6
	5a	1.72, m	29.3	1.73	29.3
	5b	1.58, m	29.3	1.60	29.3
	6	5.08, br s	73.7	5.10	73.4
	6-OH	6.28, br s			
	NH	7.13		n.d.	
Abu	1		163.6		163.4
	2		130.0		130.2
	3	6.55, q (7.1)	131.9	6.55	131.8
	4	1.51, d	13.0	1.51	13.0
	NH	9.37, br s			
Thr-1	1		169.5		

	2	4.60, br	55.4	4.60	55.2
	3	5.42, br s	71.8	5.41	71.8
	4	1.27, d (6.1)	17.8	1.27	18.2
	NH	7.69, br d (6.1)		7.72	
Thr-2	1		169.4		
	2	4.39, br	57.7	4.39	57.6
	3	4.09, br	66.5	4.10	66.4
	4	1.05, d (6.4)	18.9	1.05	18.8
	NH	7.81, br		7.83	
Ala	1		173.0		172.5
	2	4.37, m	47.9	4.38	47.9
	3	1.21, d (7.1)	17.9	1.22	17.8
	NH	8.05, d (7.5)		8.10	
Ba	1		172.5		172.0
	2	2.08, t (7.2)	37.0	2.08	36.8
	3	1.51, m	18.5	1.51	18.5
	4	0.84, t (7.3)	13.5	0.85	13.4

[a] Assignments are based on extensive 1D and 2D NMR measurements (^{1}H, HMBC, HSQC, COSY, HSQC-TOCSY). See also Figures S23-S31, n.d. = not detected.

Supplementary Table 4: Calculated mass, experimentally determined mass, and mass error for compounds **1-6** $[M+H]^+$.

**Compound name**	**Molecular formula $[M+H]^+$**	**calculated mass [Da]**	**experimentally determined mass [Da]**	**error [ppm]**
Rivulariapeptolide 1185 (**1**)	$C_{61}H_{88}N_9O_{15}^+$	1186.6394	1186.6400	0.51
Rivulariapeptolide 1155 (**2**)	$C_{59}H_{82}N_9O_{15}^+$	1156.5925	1156.5923	-0.17
Rivulariapeptolide 1121 (**3**)	$C_{56}H_{84}N_9O_{15}^+$	1122.6081	1122.6080	-0.09
Rivulariapeptolide 988 (**4**)	$C_{50}H_{69}N_8O_{13}^+$	989.4979	989.4978	-0.10
Molassamide (**5**)	$C_{48}H_{67}N_8O_{13}^+$	963.4822	963.4818	-0.41
Molassamide B (**6**)	$C_{48}H_{66}BrN_8O_{13}^+$	1041.3927	1041.3924	-0.29

Supplementary Table 5: Top 50 Chymotrypsin-Inhibitors among Ahp-cyclodepsipeptides.

Rank	NAME(S)	AA1	AA2	AA3	AA4	AA5	AA6	AA7	AA8	AA 9	Chymotrypsin IC_{50} [μM]
1	Micropeptin T-20			Hpg	Thr	Phe	Ahp	Phe	MeTyr	Ile	0.0025
2	Rivulariapeptolide 1185	Ba-Pro	Ba-Pro	Thr	Thr	Leu	Ahp	Phe	MeTyr	Val	0.0132
3	Molassamide B	Ba	Ala	Thr	Thr	Dhb	Ahp	Phe	MeTyr	Val	0.0247
4	Rivulariapeptolide 1121	Ba-Pro	Ba-Pro	Thr	Thr	Dhb	Ahp	Leu	MeTyr	Val	0.0355
5	Rivulariapeptolide 1155	Ba-Pro	Ba-Pro	Thr	Thr	Dhb	Ahp	Phe	MeTyr	Val	0.0418
6	Rivulariapeptolide 988		Ba-Pro	Thr	Thr	Dhb	Ahp	Phe	MeTyr	Val	0.0955
7	Crocapeptin A1		Pa	Gln	Thr	Leu	Ahp	Phe	MeTyr	Val	0.1
8	Crocapeptin A2		iBa	Gln	Thr	Leu	Ahp	Phe	MeTyr	Val	0.1
9	Crocapeptin A3		Pea	Gln	Thr	Leu	Ahp	Phe	MeTyr	Val	0.1
10	Bouillomide A	Ba	Ala	Val	Thr	Dhb	Ahp	Phe	MeTyr	Val	0.17
11	Crocapeptin B		iBa	Gln	Thr	Leu	Ahp	Phe	MeTyr	Ile	0.2
12	Micropeptin KB1046		Hpla	Gln	Thr	ThTyr	Ahp	Val	MePhe	Ile	0.22
13	Symplostatin 10		Msg	Ile	Thr	Dhb	Ahp	Phe	MeTyr	Ile	0.222
14	Molassamide (others)	Ba	Ala	Thr	Thr	Dhb	Ahp	Phe	MeTyr	Val	0.234
15	Loggerpeptin A	Ba	Ala	Thr	Thr	Leu	Ahp	Phe	Dmy	Val	0.24
16	Cyanopeptolin CP1018		Oa	Asp	Thr	Arg	Ahp	Phe	MePhe	Val	0.24
17	Cyanopeptolin CP992		Ba	Asp	Thr	Arg	Ahp	Phe	MeHty	Val	0.24
18	Cyanopeptolin CP1027		Ha	Asp	Thr	Tyr	Ahp	Phe	MeHty	Val	0.26
19	Cyanopeptolin CP978		Ba	Asp	Thr	Arg	Ahp	Phe	MeTyr	Val	0.26
20	Micropeptin KT1042		Hpla	Gln	Thr	Tyr	Ahp	Ile	MePhe	Val	0.26
21	Symplostatin 8		Msg	Val	Thr	Dhb	Ahp	Phe	MeTyr	Ile	0.268
22	Lyngbyastatin 4	Hsg	Ala	Hty	Thr	Dhb	Ahp	Phe	MeTyr	Val	0.3
23	Symplostatin 6		Msg	Val	Thr	Dhb	Ahp	Phe	MePhe	Val	0.322
24	Symplostatin 9		Msg	Val	Thr	Dhb	Ahp	Phe	MeTyr	Val	0.324
25	Symplocamide A		Ba	Gln	Thr	Cit	Ahp	Ile	Dmy(3-Br)	Val	0.38

26	Micropeptin 88-A			Glu	Thr	ThTyr	Ahp	Val	MePhe	Ile	0.4
27	Symplostatin 7		Msg	Ile	Thr	Dhb	Ahp	Phe	MePhe	Ile	0.515
28	Tutuilamide C		MeCmb	Val	Thr	Dhb	Ahp	Phe	MeTyr	Val	0.542
29	Tutuilamide B		Cmb	Val	Thr	Dhb	Ahp	Phe	MeTyr	Val	0.577
30	Micropeptin KB1048		Ha	Asp(Me)	Thr	Arg	Amp	Ile	MeTyr(3-Cl)	Ile	0.63
31	Microcystin 996		Ba	Gln	Thr	Hty	Ahp	Phe	MePhe	Val	0.64
32	Planktopeptin BL1125	Gla	Hty	Gln	Thr	Leu	Ahp	Thr	Dmy	Ile	0.8
33	Molassamide (this study)	Ba	Ala	Thr	Thr	Dhb	Ahp	Phe	MeTyr	Val	0.862
34	Micropeptin KB991		Hpla	Gln	Thr	Leu	Amp	Ile	MeTyr(3-Cl)	Val	0.87
35	Cyanopeptolin 963A		Ha	Asp	Thr	Tyr	Ahp	Leu	MePhe	Val	0.9
36	Largamide D oxazolidine	Gla-Ahppa	Ala	Val	Thr	Leu	Ahp[a]	allo-Thr	MeTyr(3-Br)	Val	0.928
37	Tutuilamide A		Cmb	Ile	Thr	Dhb	Ahp	Phe	MeTyr	Val	1
38	Micropeptin 103	Ha	Gly	Thr	Thr	Gln	Ahp	Phe	MeTrp	Val	1
39	Micropeptin E		Ha	Glu	Thr	Tyr	Ahp	Leu	MeTyr	Val	1
40	Dinghupepeptin B		Mba	Gln	Thr	NHeGln	Amp	Phe	MeTyr	Ala	1.1
41	Micropeptin C		Ha	Glu	Thr	Tyr	Ahp	Phe	MeTyr	Val	1.1
42	Micropeptin LH1021		Ha	Thr	Thr	Gln	Ahp	Phe	MeTyr	Val	1.1
43	Micropeptin D		Ha	Glu	Thr	Tyr	Ahp	Leu	MeTyr	Val	1.2
44	Micropeptin 88-Y	Ac	Tyr	Glue	Thr	Tyr	Ahp	Val	MePhe	Ile	1.3
45	Micropeptin MM836			Gla	Thr	Leu	Ahp	Phe	MePhe	Ile	1.4
46	Jizanpeptin C		Msg	Val	Thr	Lys	Ahp	allo-Ile	Dmy(3-Br)	Ile	1.4
47	Micropeptin F		Oa	Glu	Thr	Tyr	Ahp	Leu	MeTyr	Val	1.5
48	Nostopeptin B		Ac	Gln	2S,3R,4 R-Hmp	Leu	Ahp	Ile	MeTyr	Ile	1.6
49	Micropeptin DR1056		Hpla	Gln	Thr	Tyr	Ahp	Leu	MePhe	Ile	1.6
50	Micropeptin MM850			Gla	Thr	Leu	Amp	Phe	MePhe	Ile	1.7

Abbreviations for amino acids/residues (AA) found in Ahp-cyclodepsipetides: Ac: acetic acid; Ahppa: 2-amino-5-(4'-methoxyphenyl)pentanoic acid; Ba: butyric acid; Dmy: *N,O*-dimethyltyrosine; Gla: Glyceric acid; Ha: hexanoic acid; Hpla: 2-Hydroxy-3-(4-hydroxyphenyl)propanoic acid; Hpg: 3'-*O*-phosphate (R)-glyceric acid; Hsg: 3-O-sulfated (R)-glyceric acid; Hty: Homotyrosine; iBa: iso-Butyric acid; Mba: methyl-2-butenoic acid; MePhe:(3',4'-OH): 3',4'-Hydroxy-*N*-methylphenylalanine; Msg: 2-*O*-methyl-3-*O*-sulfate-(R)-glyceric acid; Oa: Octanoic acid; Pa: Propionic acid; Pea:2-pentanic acid; ThTyr: Tetrahydrotyrosine**.**
[a]OH-Group is linked to the Thr-subsite (AA 7) and forms an oxazolidine.

Supplementary References

1. Reher, R. *et al.* A Convolutional Neural Network-Based Approach for the Rapid Annotation of Molecularly Diverse Natural Products. *J. Am. Chem. Soc.* **142**, 4114–4120 (2020).
2. Palmer, M. R. *et al.* Sensitivity of Nonuniform Sampling NMR. *The Journal of Physical Chemistry B* **119**, 6502–6515 (2015).
3. Ndukwe, I. E., Shchukina, A., Kazimierczuk, K. & Butts, C. P. Rapid and safe ASAP acquisition with EXACT NMR. *Chemical Communications* **52**, 12769–12772 (2016).
4. Kim, H. W. *et al.* NPClassifier: A Deep Neural Network-Based Structural Classification Tool for Natural Products. *J. Nat. Prod.* **84**, 2795–2807 (2021).
5. Dührkop, K. *et al.* Systematic classification of unknown metabolites using high-resolution fragmentation mass spectra. *Nat Biotechnol* **39**, 462–471 (2021).
6. Wang, M. *et al.* Sharing and community curation of mass spectrometry data with Global Natural Products Social Molecular Networking. *Nature Biotechnology* **34**, 828–837 (2016).
7. Mehner, C. *et al.* New Peptolides from the Cyanobacterium Nostoc insulare as Selective and Potent Inhibitors of Human Leukocyte Elastase. *ChemBioChem* **9**, 2692–2703 (2008).
8. Keller, L. *et al.* Tutuilamides A–C: Vinyl-Chloride-Containing Cyclodepsipeptides from Marine Cyanobacteria with Potent Elastase Inhibitory Properties. *ACS Chem. Biol.* **15**, 751–757 (2020).

9. Gunasekera, S. P., Miller, M. W., Kwan, J. C., Luesch, H. & Paul, V. J. Molassamide, a Depsipeptide Serine Protease Inhibitor from the Marine Cyanobacterium Dichothrix utahensis. *J. Nat. Prod.* **73**, 459–462 (2010).

10. Araya-Maturana, R., Pessoa-Mahana, H. & Weiss-López, B. Very Long-Range Correlations ($^{n}J_{C,H}$ n > 3) in HMBC Spectra. *Natural Product Communications* **3**, 1934578X0800300 (2008).

11. Salvador, L. A. *et al.* Potent Elastase Inhibitors from Cyanobacteria: Structural Basis and Mechanisms Mediating Cytoprotective and Anti-Inflammatory Effects in Bronchial Epithelial Cells. *J. Med. Chem.* **56**, 1276–1290 (2013).

12. Linington, R. G. *et al.* Symplocamide A, a Potent Cytotoxin and Chymotrypsin Inhibitor from the Marine Cyanobacterium Symploca sp. *J. Nat. Prod.* **71**, 22–27 (2008).

13. Köcher, S. *et al.* From dolastatin 13 to cyanopeptolins, micropeptins, and lyngbyastatins: the chemical biology of Ahp-cyclodepsipeptides. *Nat. Prod. Rep.* (2019) doi:10.1039/C9NP00033J.

14. Al-Awadhi, F. H., Paul, V. J. & Luesch, H. Structural Diversity and Anticancer Activity of Marine-Derived Elastase Inhibitors: Key Features and Mechanisms Mediating the Antimetastatic Effects in Invasive Breast Cancer. *ChemBioChem* **19**, 815–825 (2018).

15. Silber, N. *et al.* Cell Division Protein FtsZ Is Unfolded for N-Terminal Degradation by Antibiotic-Activated ClpP. *mBio* **11**, e01006-20 (2020).
