# Supplementary material for: Native metabolomics identifies the rivulariapeptolide family of protease inhibitors
Source: Nat Commun. 2022 Aug 8;13:4619. doi: 10.1038/s41467-022-32016-6 (PMC9358669; doi:10.1038/s41467-022-32016-6)
Supplement: Supplementary file 3 — Reporting Summary [file 41467_2022_32016_MOESM3_ESM.pdf]

nature research

Corresponding author(s): William H. Gerwick
Daniel Petras

Last updated by author(s): Jul 1, 2022

# Reporting Summary

Nature Research wishes to improve the reproducibility of the work that we publish. This form provides structure for consistency and transparency in reporting. For further information on Nature Research policies, see our Editorial Policies and the Editorial Policy Checklist.

## Statistics

For all statistical analyses, confirm that the following items are present in the figure legend, table legend, main text, or Methods section.

| n/a | Confirmed | |
|---|---|---|
| ☐ | ☒ | The exact sample size (*n*) for each experimental group/condition, given as a discrete number and unit of measurement |
| ☐ | ☒ | A statement on whether measurements were taken from distinct samples or whether the same sample was measured repeatedly |
| ☐ | ☒ | The statistical test(s) used AND whether they are one- or two-sided<br>*Only common tests should be described solely by name; describe more complex techniques in the Methods section.* |
| ☒ | ☐ | A description of all covariates tested |
| ☒ | ☐ | A description of any assumptions or corrections, such as tests of normality and adjustment for multiple comparisons |
| ☐ | ☒ | A full description of the statistical parameters including central tendency (e.g. means) or other basic estimates (e.g. regression coefficient) AND variation (e.g. standard deviation) or associated estimates of uncertainty (e.g. confidence intervals) |
| ☒ | ☐ | For null hypothesis testing, the test statistic (e.g. *F*, *t*, *r*) with confidence intervals, effect sizes, degrees of freedom and *P* value noted<br>*Give P values as exact values whenever suitable.* |
| ☒ | ☐ | For Bayesian analysis, information on the choice of priors and Markov chain Monte Carlo settings |
| ☒ | ☐ | For hierarchical and complex designs, identification of the appropriate level for tests and full reporting of outcomes |
| ☒ | ☐ | Estimates of effect sizes (e.g. Cohen's *d*, Pearson's *r*), indicating how they were calculated |

*Our web collection on statistics for biologists contains articles on many of the points above.*

## Software and code

Policy information about availability of computer code

| | |
|---|---|
| Data collection | Mass Spectrometry data was collected with Xcalibur (Thermo-Fisher). NMR data was collected with TopSpin (Bruker Biospin). Fluorescence data was acquired with Gen5 (BioTek). |
| Data analysis | Mass Spectrometry data was processed and analyzed with MSconvert (proteowizard), Qualbrowser (Thermo-Fisher), MZmine2, GNPS, SIRIUS, and Python (code is shared on github and zenodo). NMR data was analyzed with MestReNova (Mnova), and SMART. Data was plotted using GraphPad Prism and Microsoft Excel. Figures were prepared in Illustrator (Adobe) and Microsoft PowerPoint. |

For manuscripts utilizing custom algorithms or software that are central to the research but not yet described in published literature, software must be made available to editors and reviewers. We strongly encourage code deposition in a community repository (e.g. GitHub). See the Nature Research guidelines for submitting code & software for further information.

## Data

Policy information about availability of data

All manuscripts must include a data availability statement. This statement should provide the following information, where applicable:

- Accession codes, unique identifiers, or web links for publicly available datasets
- A list of figures that have associated raw data
- A description of any restrictions on data availability

All raw (.raw), deconvoluted (xtract.raw) and centroided (.mzXML or .mzML) mass spectrometry data as well as processed data feature table (.csv) and MS/MS spectra (.mgf) are available through the MassIVE repository (massive.ucsd.edu) with the identifier MSV000087964, MSV000088586 and MSV000088578. The MS/MS spectra of the new discovered derivatives, including tags as protease inhibitors, have been added to the GNPS library (gnps.ucsd.edu) with the following IDs: rivulariapeptolide 1185 (1): CCMSLIB00005723387; rivulariapeptolide 1155 (2): CCMSLIB00005723986, CCMSLIB00005720236; rivulariapeptolide 1121 (3): CCMSLIB00005723398; rivulariapeptolide 988 (4): CCMSLIB00005723393; molassamide (5): CCMSLIB00005723404; molassamide B (6): CCMSLIB00006710020. Raw

NMR data for compounds 1 -6 has been deposited to Zenodo (zenodo.org) and can be accessed under the following link: https://sandbox.zenodo.org/record/905199.

# Field-specific reporting

Please select the one below that is the best fit for your research. If you are not sure, read the appropriate sections before making your selection.

☒ Life sciences ☐ Behavioural & social sciences ☐ Ecological, evolutionary & environmental sciences

For a reference copy of the document with all sections, see nature.com/documents/nr-reporting-summary-flat.pdf

# Life sciences study design

All studies must disclose on these points even when the disclosure is negative.

| | |
|---|---|
| Sample size | The study was performed on crude extract and purified fractions. No statistical methods where used to determine sample size. Validation experiments where performed in technical triplicates (Mass Spectrometry) and in triplicates of distinct samples (Protease Inhibition Assays). |
| Data exclusions | No data was excluded. |
| Replication | Validation experiments where performed in technical triplicates (Mass Spectrometry) and in triplicates of distinct samples (protease inhibition assays). |
| Randomization | Due to the small sample size and distinct LC-MS settings, no randomization was performed. |
| Blinding | Due to the small sample size and distinct LC-MS settings, no blinding was performed. |

# Reporting for specific materials, systems and methods

We require information from authors about some types of materials, experimental systems and methods used in many studies. Here, indicate whether each material, system or method listed is relevant to your study. If you are not sure if a list item applies to your research, read the appropriate section before selecting a response.

## Materials & experimental systems

| n/a | Involved in the study | |
|---|---|---|
| ☒ | ☐ | Antibodies |
| ☒ | ☐ | Eukaryotic cell lines |
| ☒ | ☐ | Palaeontology and archaeology |
| ☒ | ☐ | Animals and other organisms |
| ☒ | ☐ | Human research participants |
| ☒ | ☐ | Clinical data |
| ☒ | ☐ | Dual use research of concern |

## Methods

| n/a | Involved in the study | |
|---|---|---|
| ☒ | ☐ | ChIP-seq |
| ☒ | ☐ | Flow cytometry |
| ☒ | ☐ | MRI-based neuroimaging |
